# Supplementary material for: Enantioselective decarboxylative chlorination of β-ketocarboxylic acids
Source: Nat Commun. 2017 Jun 5;8:15600. doi: 10.1038/ncomms15600 (PMC5465354; doi:10.1038/ncomms15600)
Supplement: Supplementary Information — Supplementary figures, supplementary tables, supplementary methods and supplementary references. [file ncomms15600-s1.pdf]

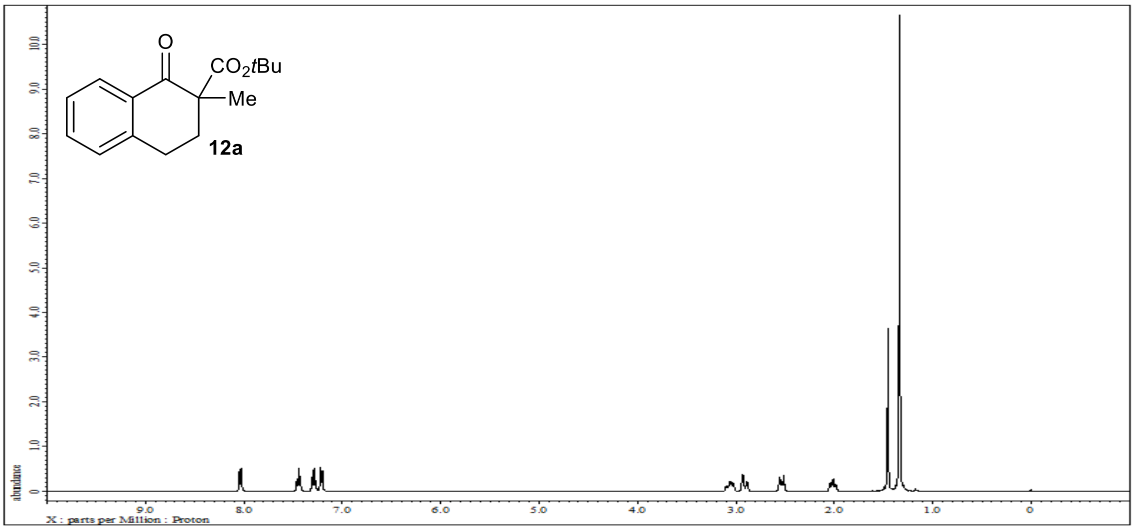

Supplementary Figure 1. ^{1}H NMR spectrum for β-ketoester **12a**.

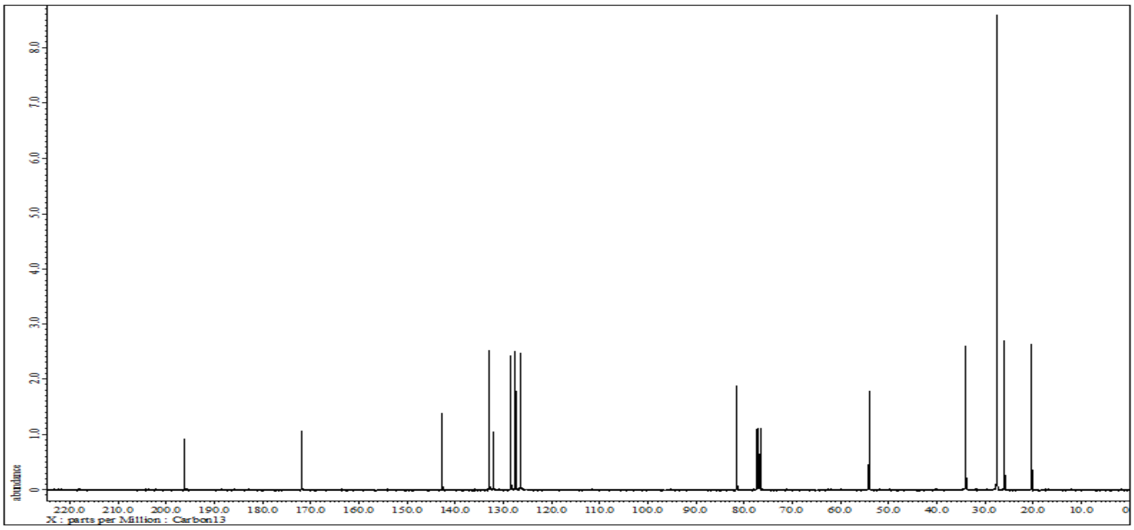

Supplementary Figure 2. ^{13}C NMR spectrum for β-ketoester **12a**.

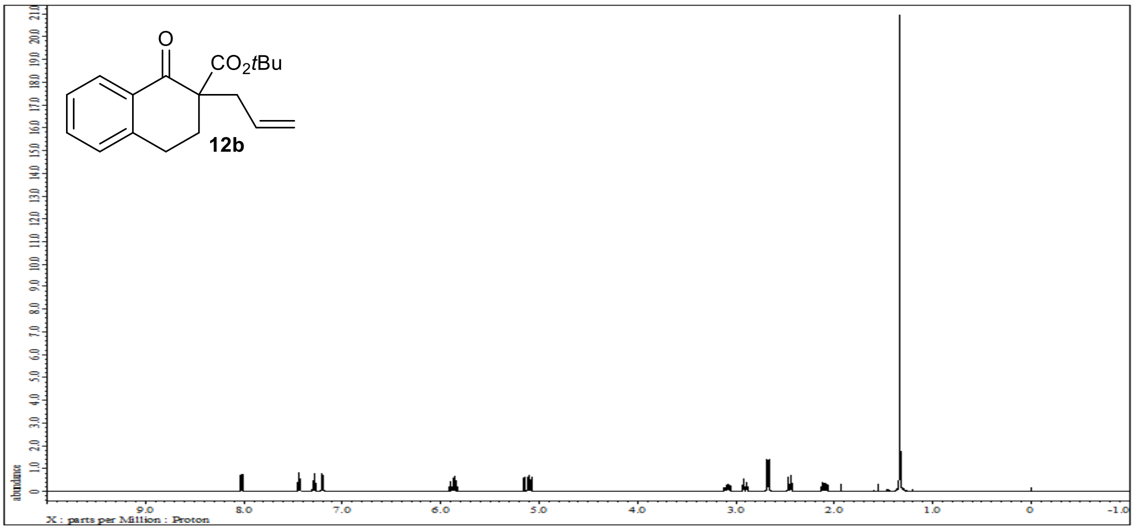

Supplementary Figure 3. ^{1}H NMR spectrum for β-ketoester **12b**.

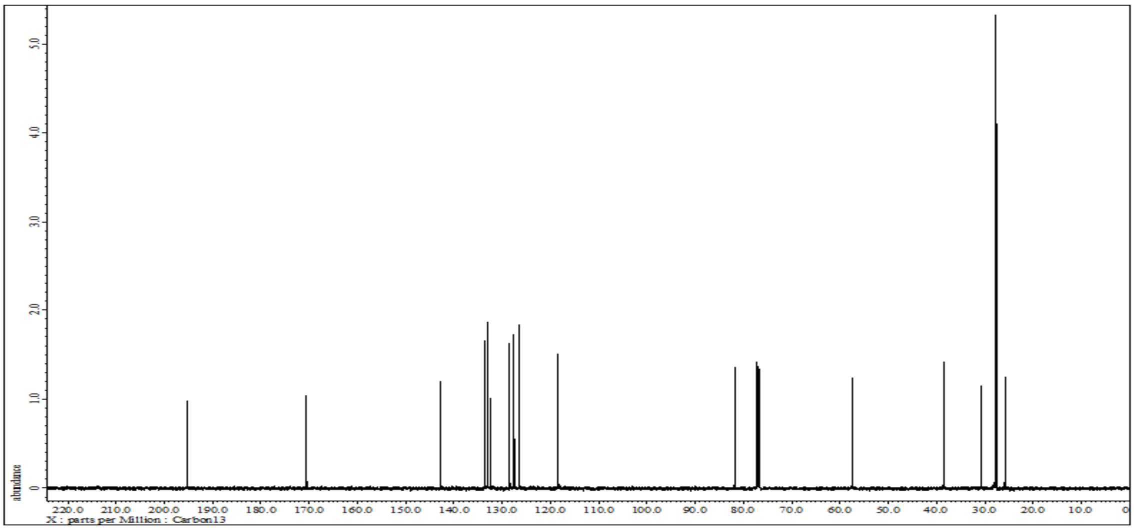

Supplementary Figure 4. ^{13}C NMR spectrum for β-ketoester **12b**.

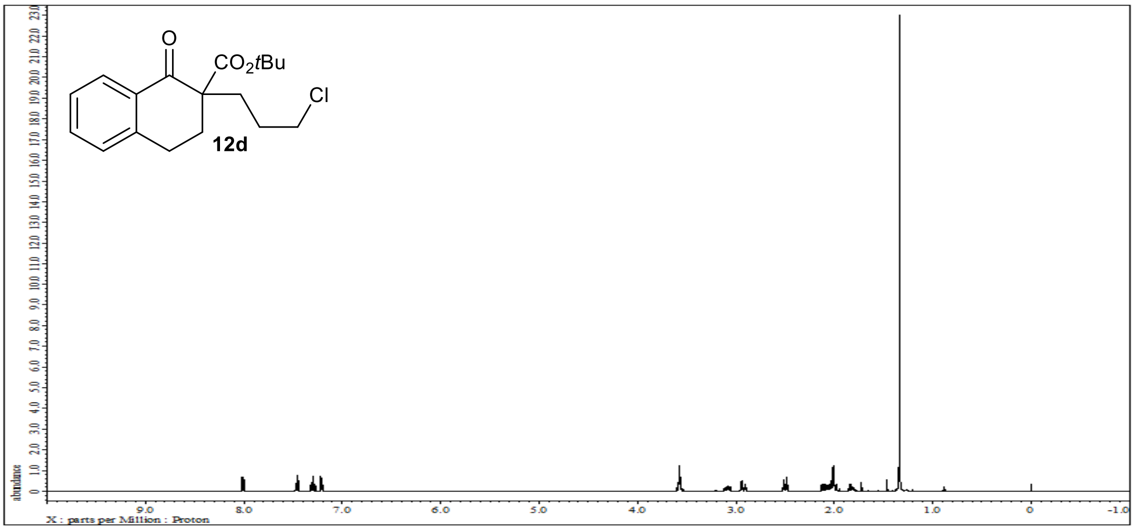

Supplementary Figure 5. ^{1}H NMR spectrum for β-ketoester **12d**.

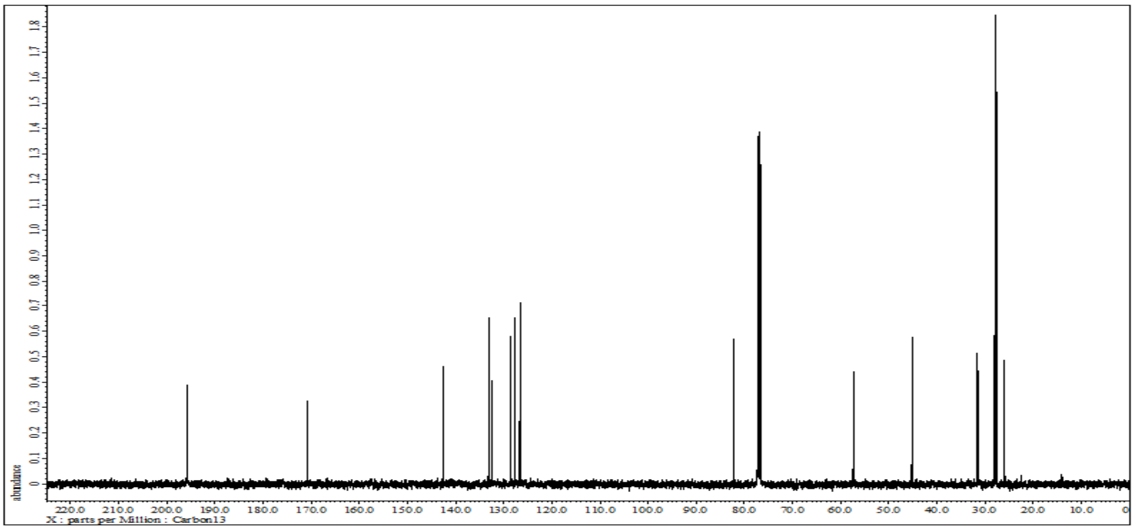

Supplementary Figure 6. ^{13}C NMR spectrum for β-ketoester **12d**.

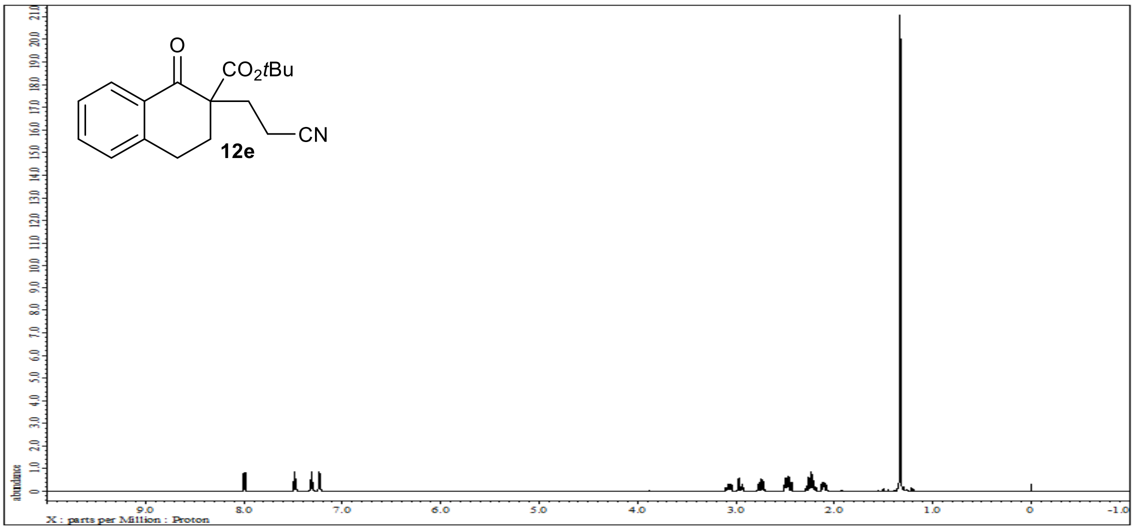

Supplementary Figure 7. ^{1}H NMR spectrum for β-ketoester **12e**.

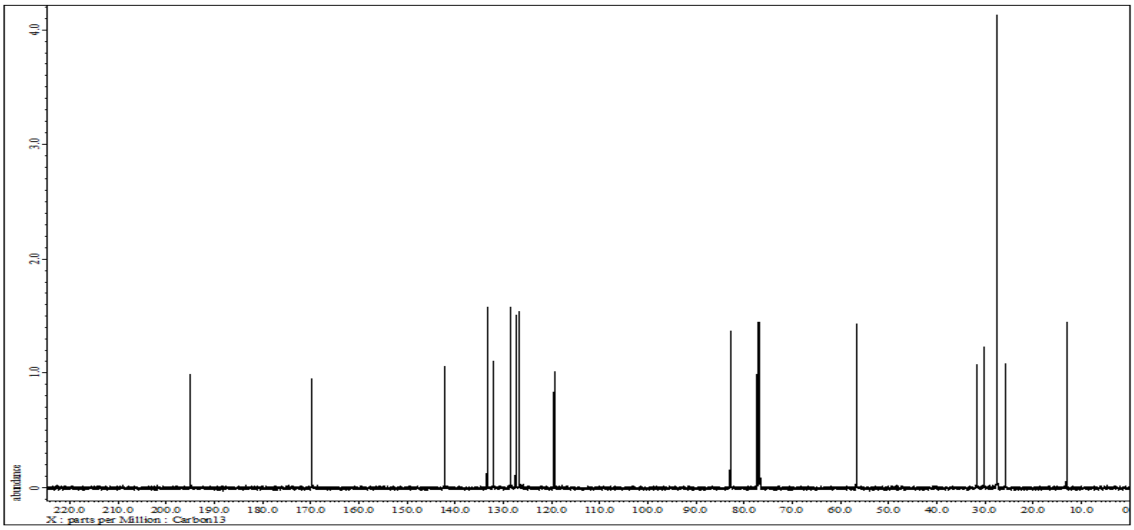

Supplementary Figure 8. ^{13}C NMR spectrum for β-ketoester **12e**.

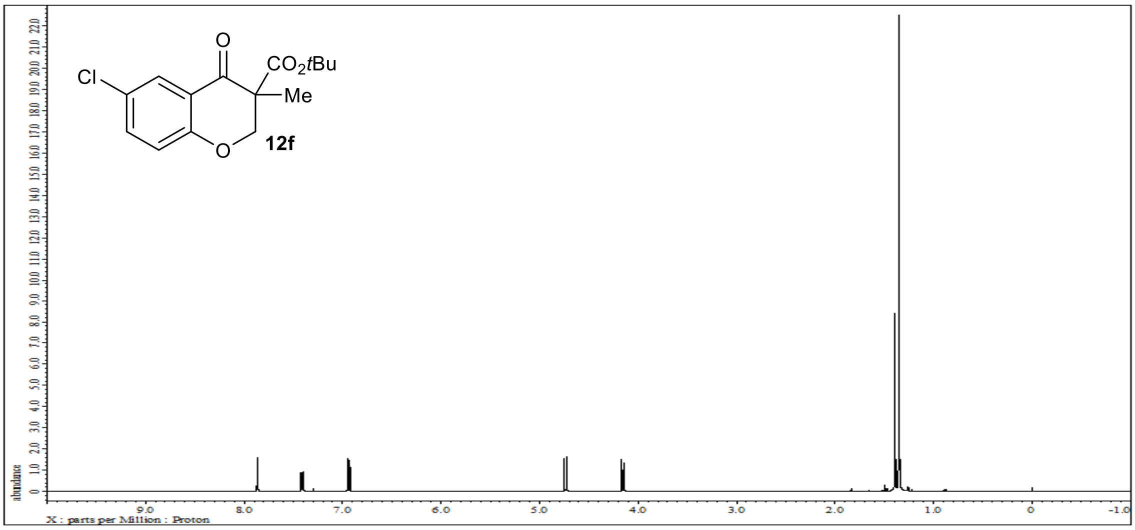

Supplementary Figure 9. ^{1}H NMR spectrum for β-ketoester **12f**.

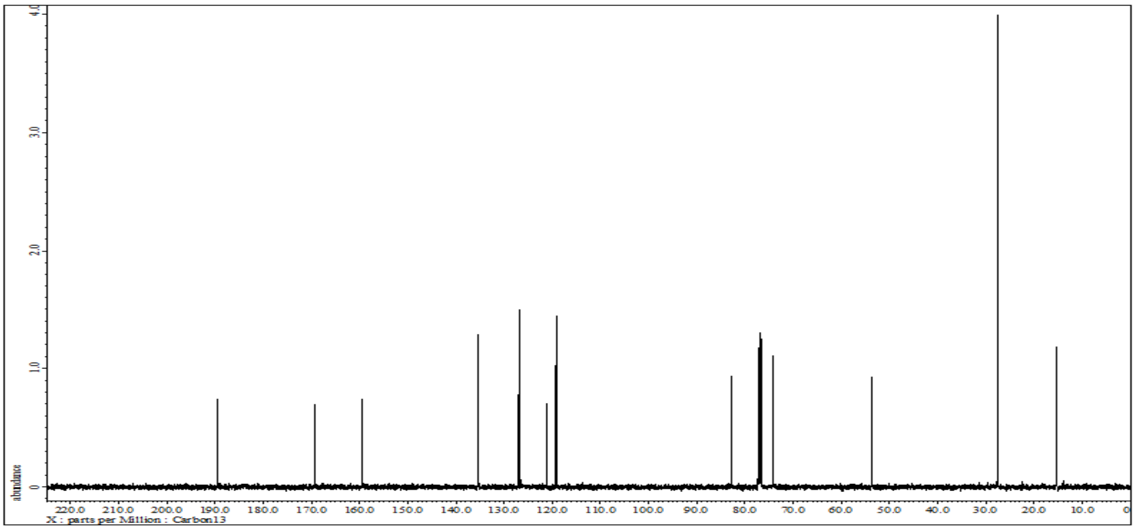

Supplementary Figure 10. ^{13}C NMR spectrum for β-ketoester **12f**.

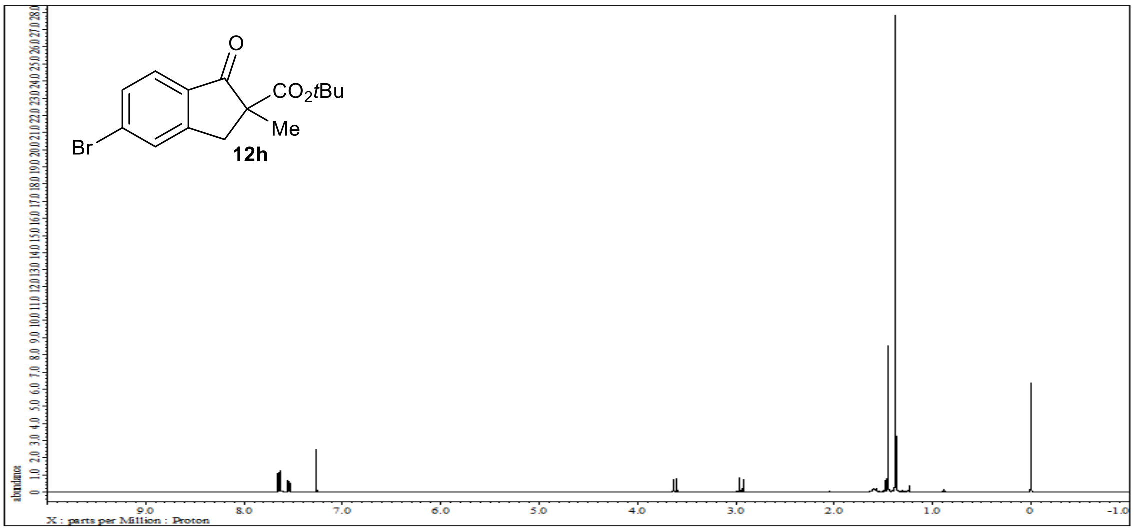

Supplementary Figure 11. ^{1}H NMR spectrum for β-ketoester **12h**.

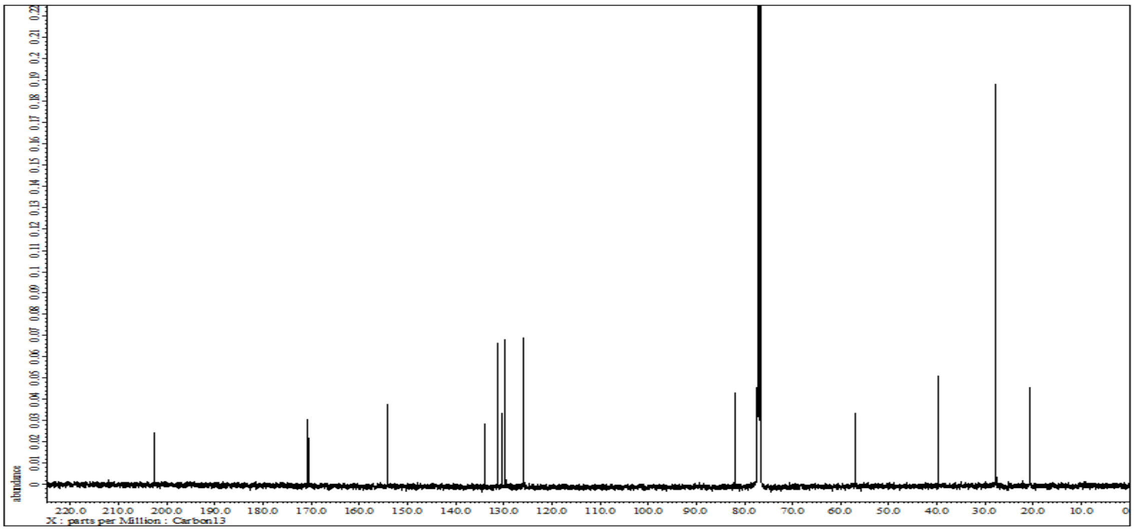

Supplementary Figure 12. ^{13}C NMR spectrum for β-ketoester **12h**.

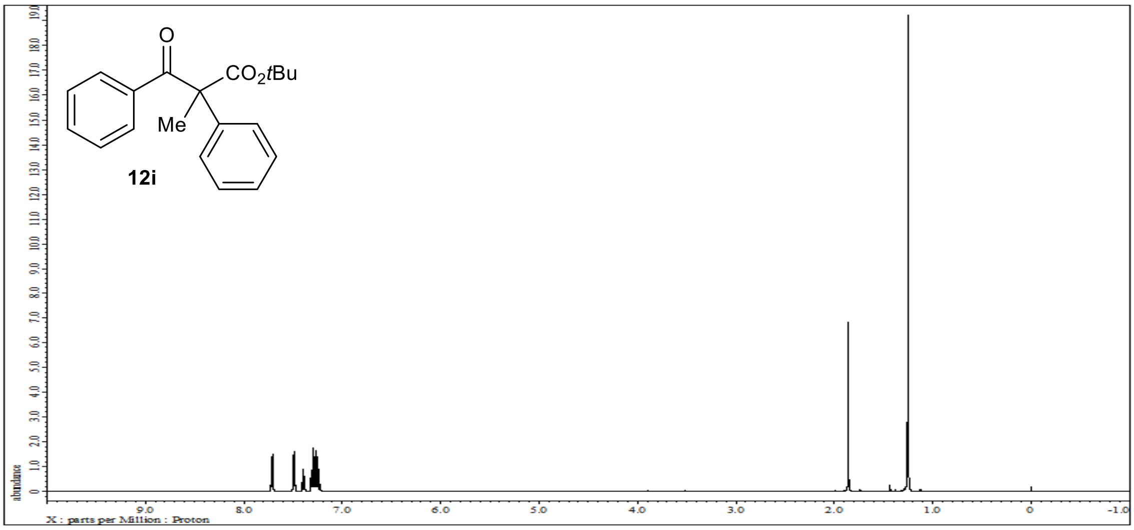

Supplementary Figure 13. ^{1}H NMR spectrum for β-ketoester **12i**.

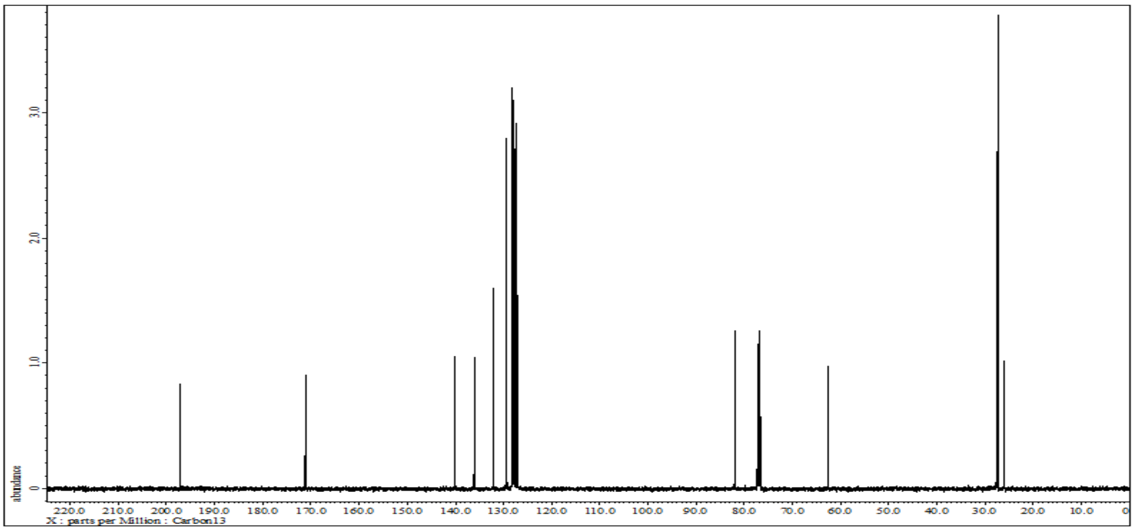

Supplementary Figure 14. ^{13}C NMR spectrum for β-ketoester **12i**.

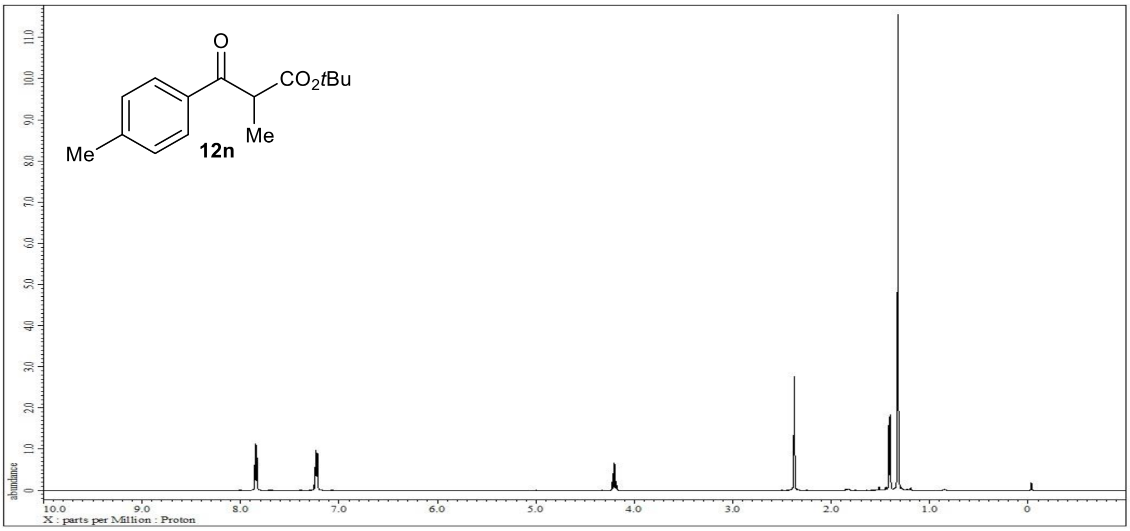

Supplementary Figure 15. ^{1}H NMR spectrum for β-ketoester **12n**.

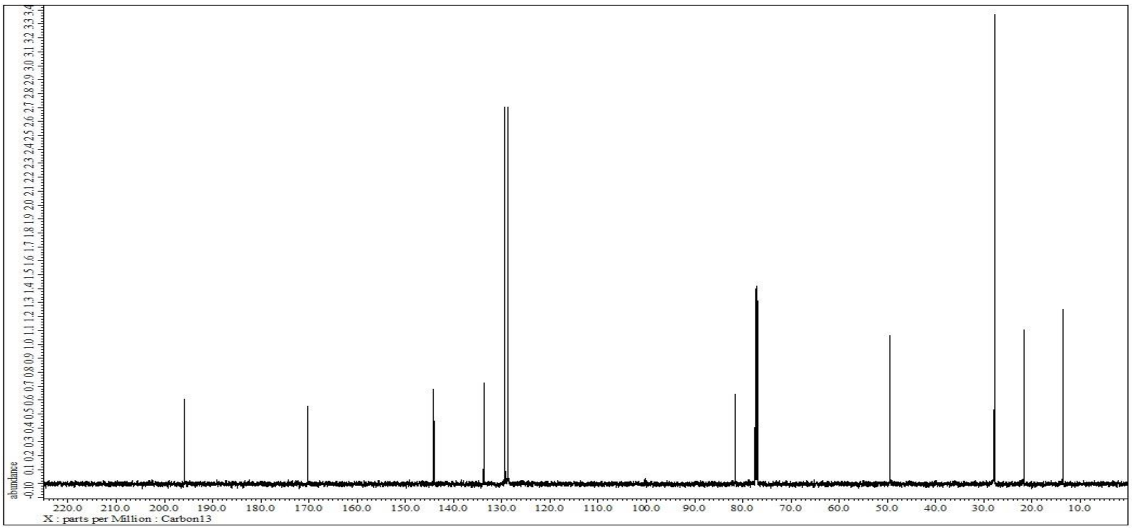

Supplementary Figure 16. ^{13}C NMR spectrum for β-ketoester **12n**.

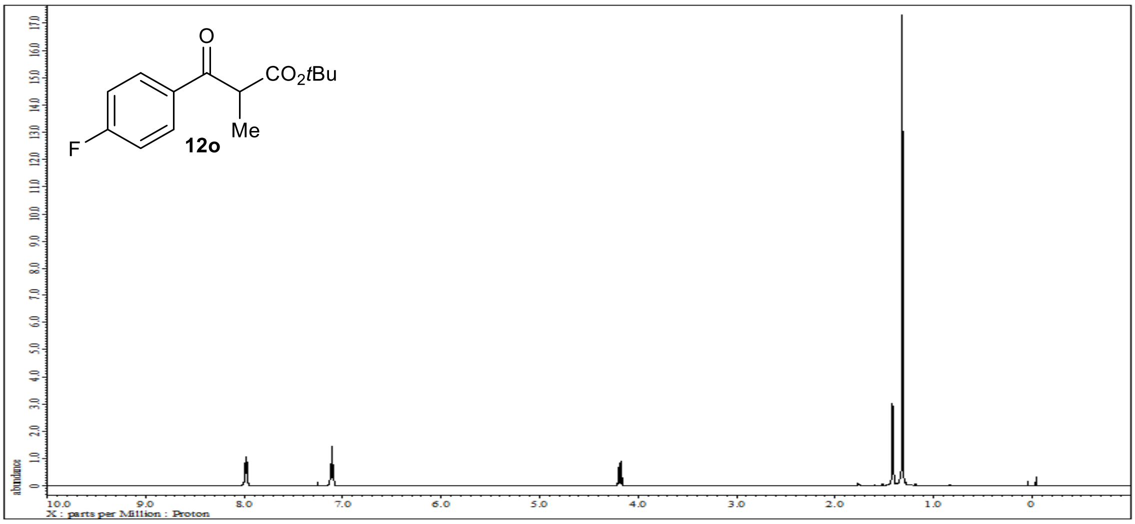

Supplementary Figure 17. ^{1}H NMR spectrum for β-ketoester **12o**.

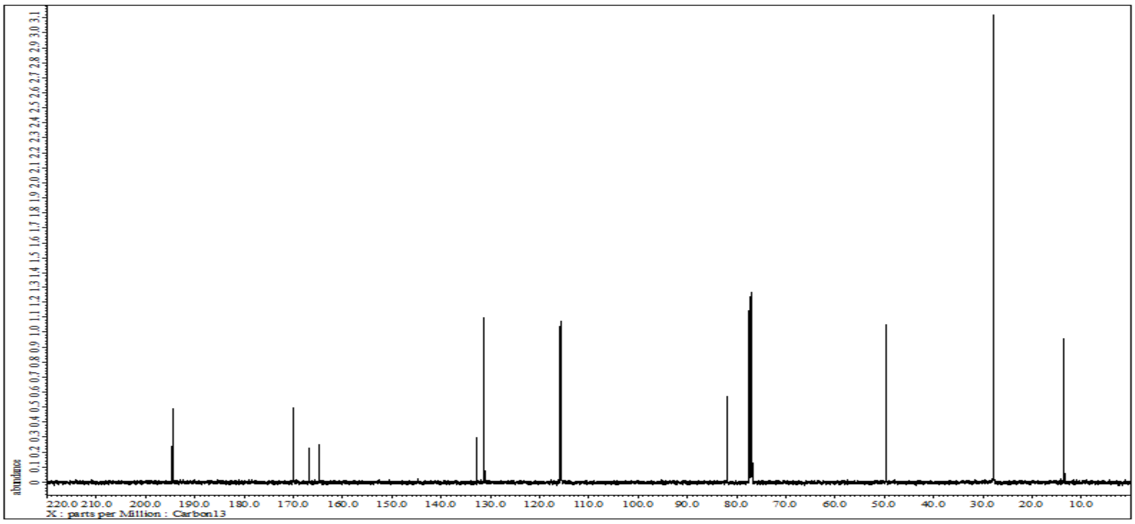

Supplementary Figure 18. ^{13}C NMR spectrum for β-ketoester **12o**.

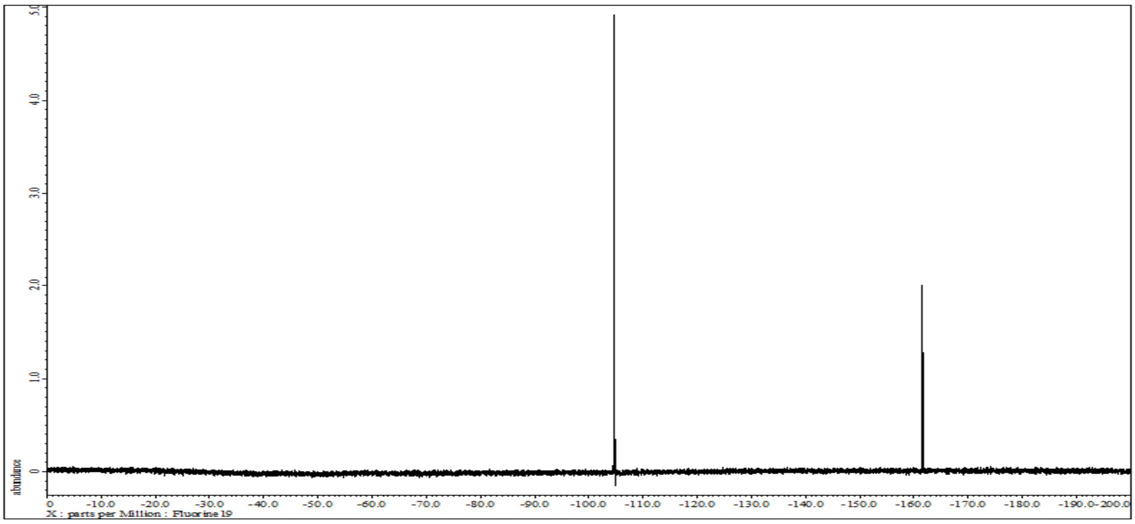

Supplementary Figure 19. ^{19}F NMR spectrum for β-ketoester **12o**.

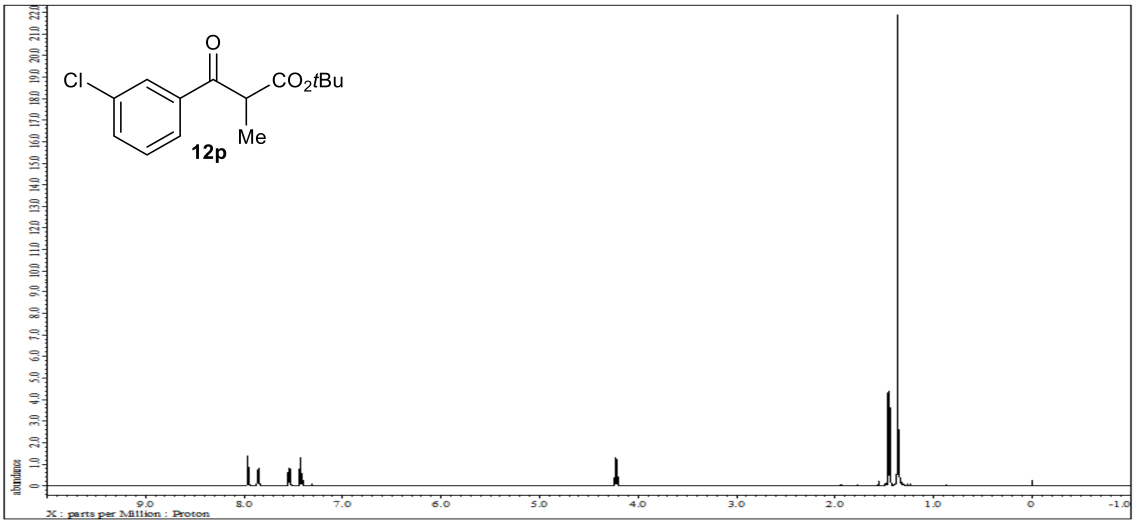

Supplementary Figure 20. ^{1}H NMR spectrum for β-ketoester **12p**.

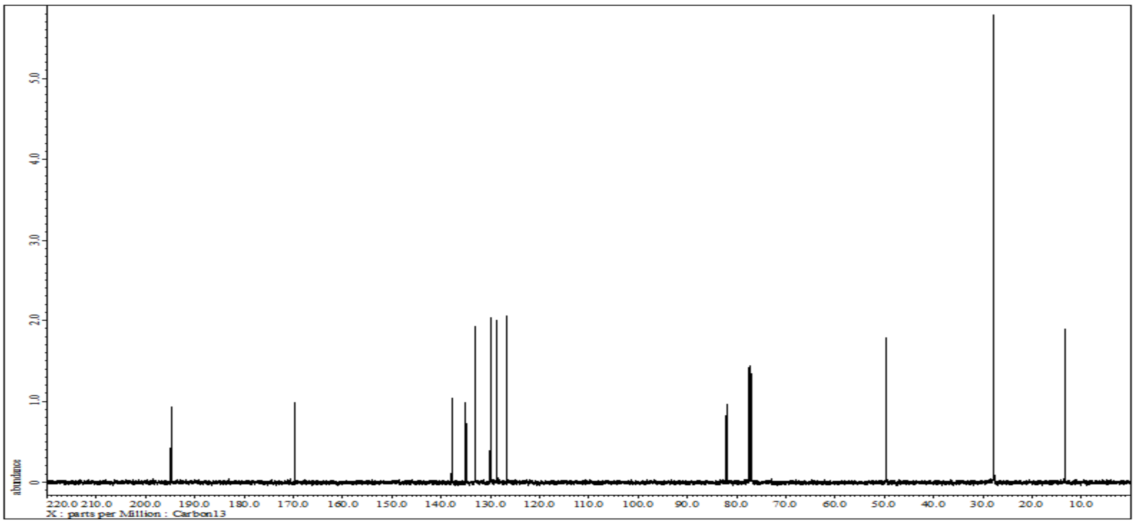

Supplementary Figure 21. ^{13}C NMR spectrum for β-ketoester **12p**.

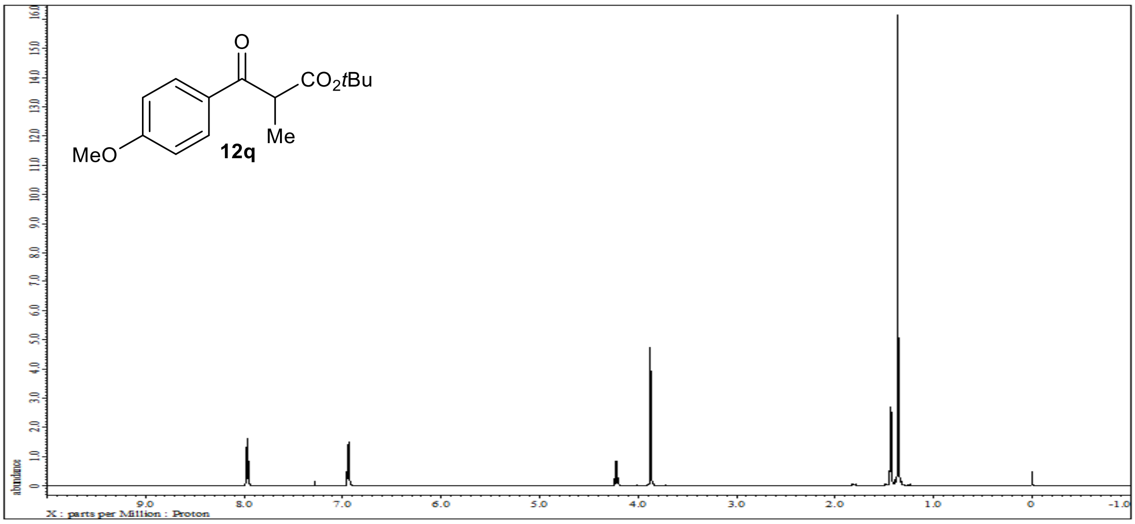

Supplementary Figure 22. ^{1}H NMR spectrum for β-ketoester **12q**.

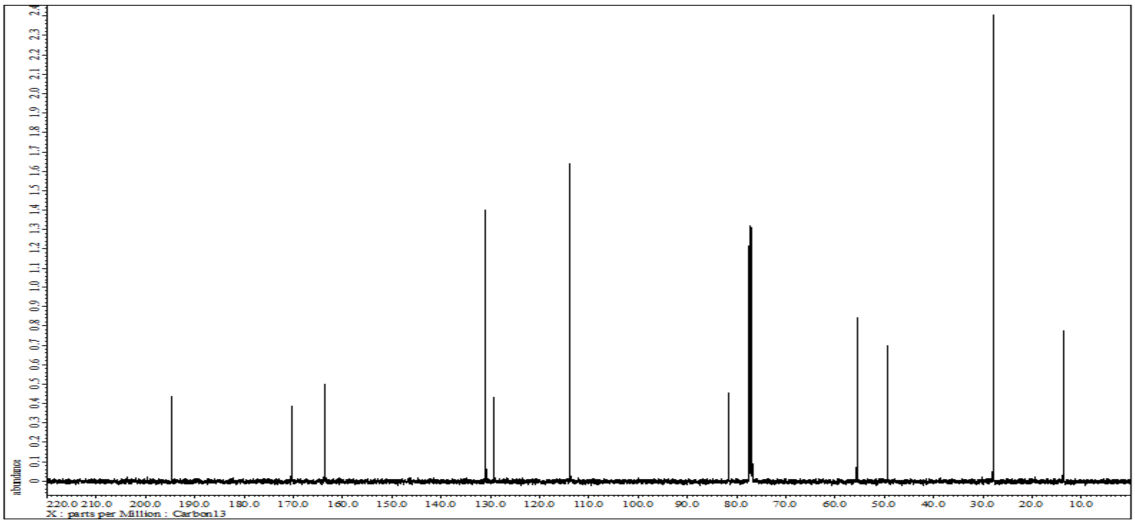

Supplementary Figure 23. ^{13}C NMR spectrum for β-ketoester **12q**.

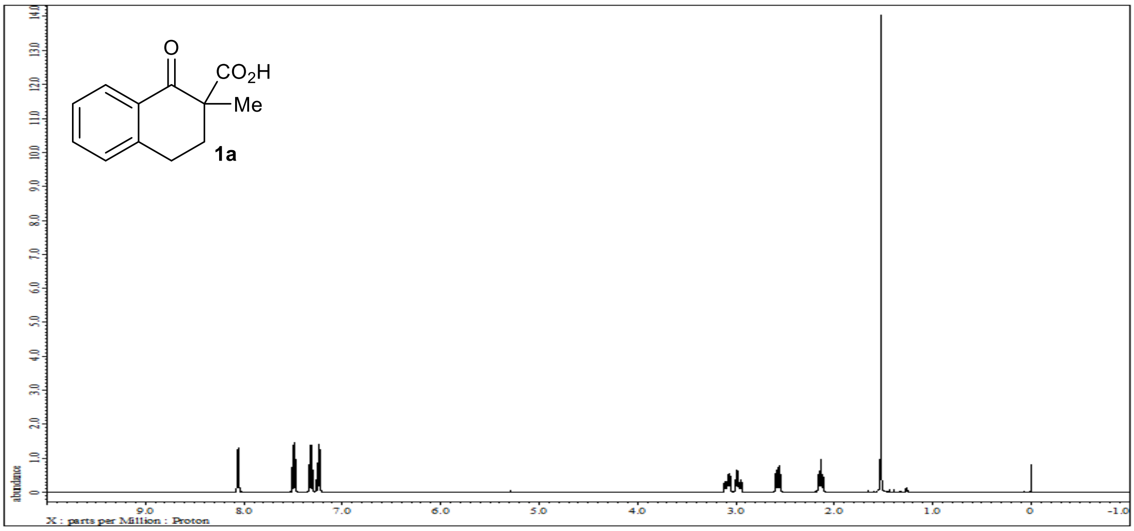

Supplementary Figure 24. ^{1}H NMR spectrum for β-ketocarboxylic acid **1a**.

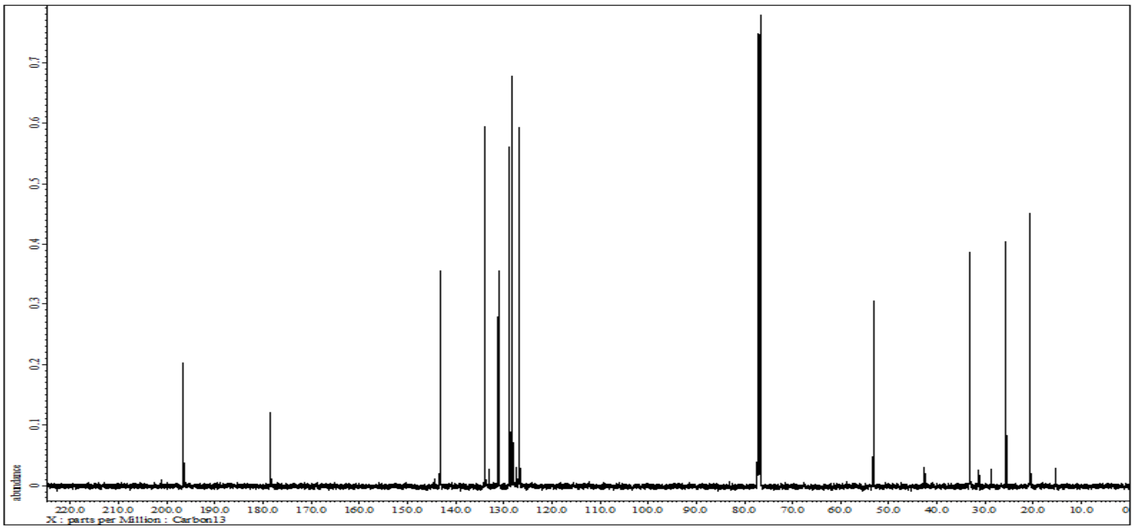

Supplementary Figure 25. ^{13}C NMR spectrum for β-ketocarboxylic acid **1a**.

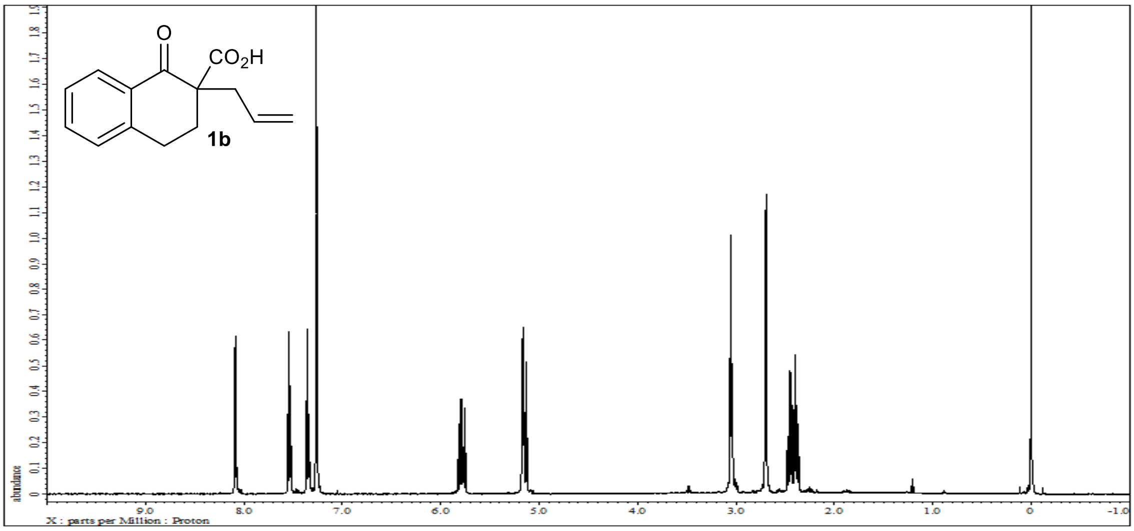

Supplementary Figure 26. ^{1}H NMR spectrum for β-ketocarboxylic acid **1b**.

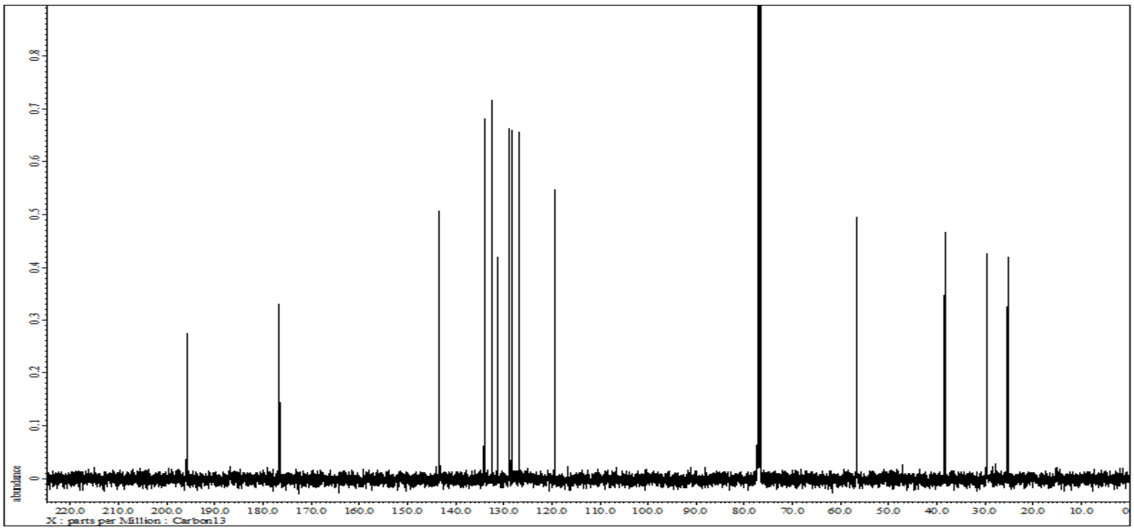

Supplementary Figure 27. ^{13}C NMR spectrum for β-ketocarboxylic acid **1b**.

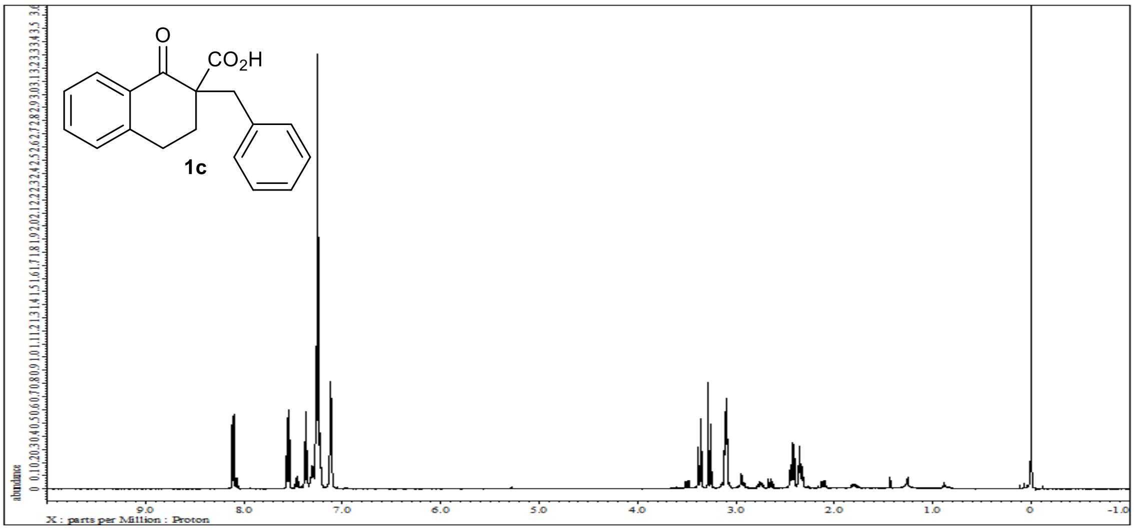

Supplementary Figure 28. ^{1}H NMR spectrum for β-ketocarboxylic acid **1c**.

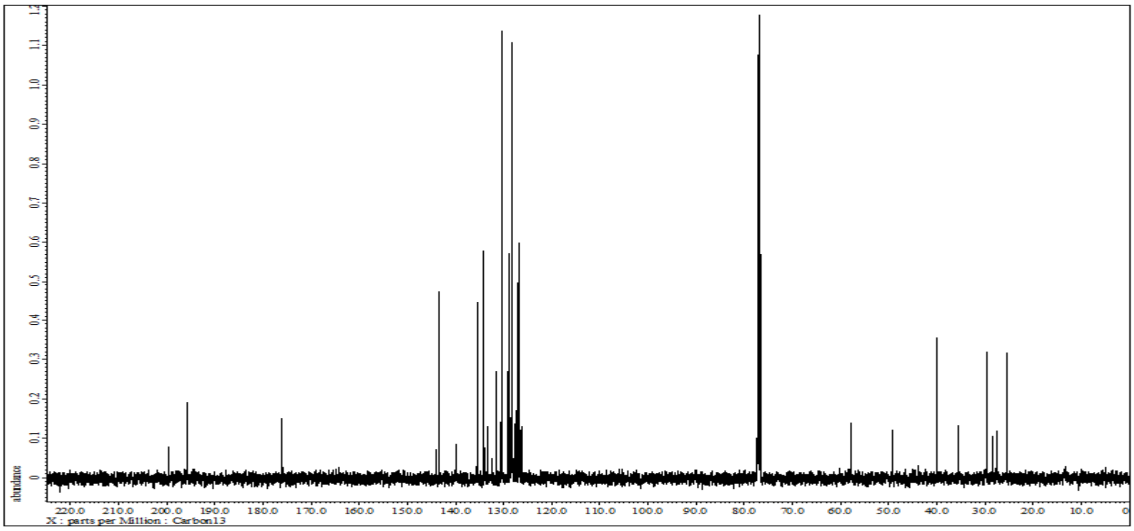

Supplementary Figure 29. ^{13}C NMR spectrum for β-ketocarboxylic acid **1c**.

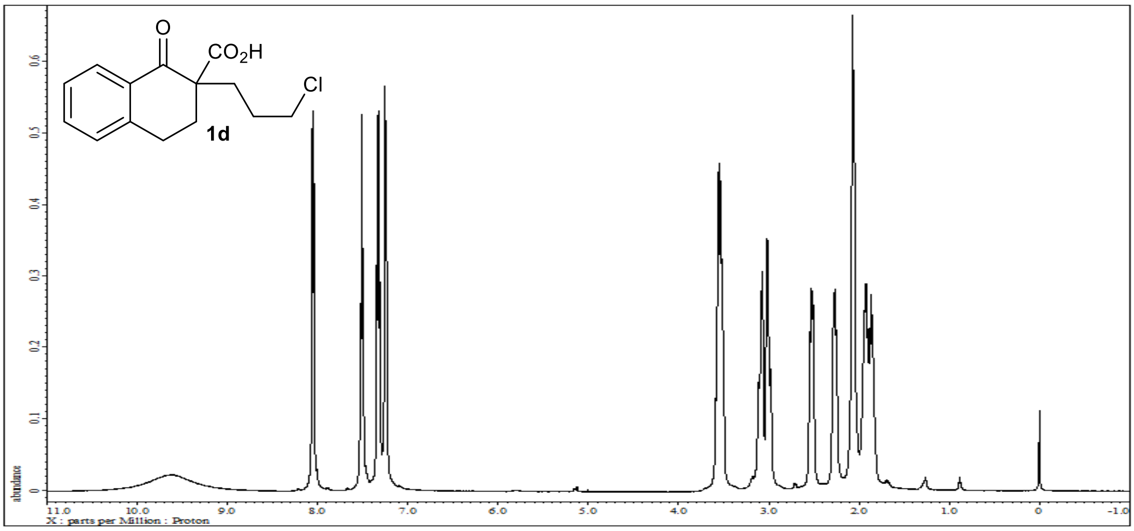

Supplementary Figure 30. ^{1}H NMR spectrum for β-ketocarboxylic acid **1d**.

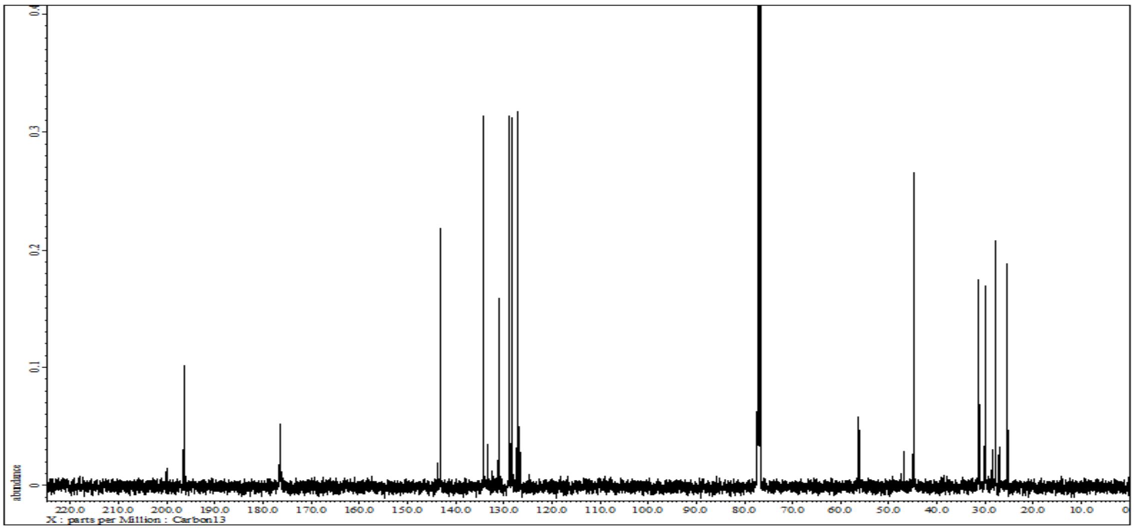

Supplementary Figure 31. ^{13}C NMR spectrum for β-ketocarboxylic acid **1d**.

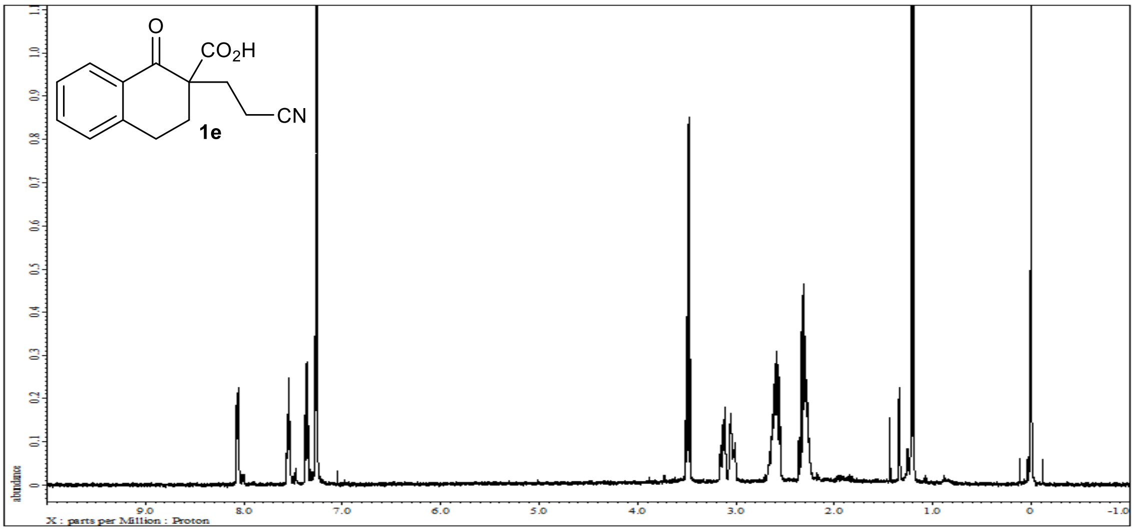

Supplementary Figure 32. ^{1}H NMR spectrum for β-ketocarboxylic acid **1e**.

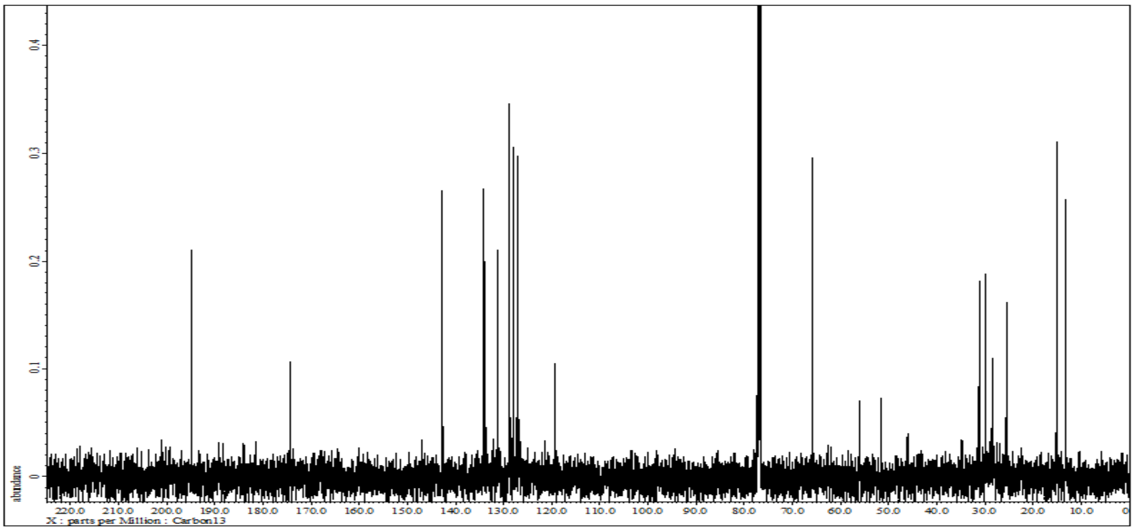

Supplementary Figure 33. ^{13}C NMR spectrum for β-ketocarboxylic acid **1e**.

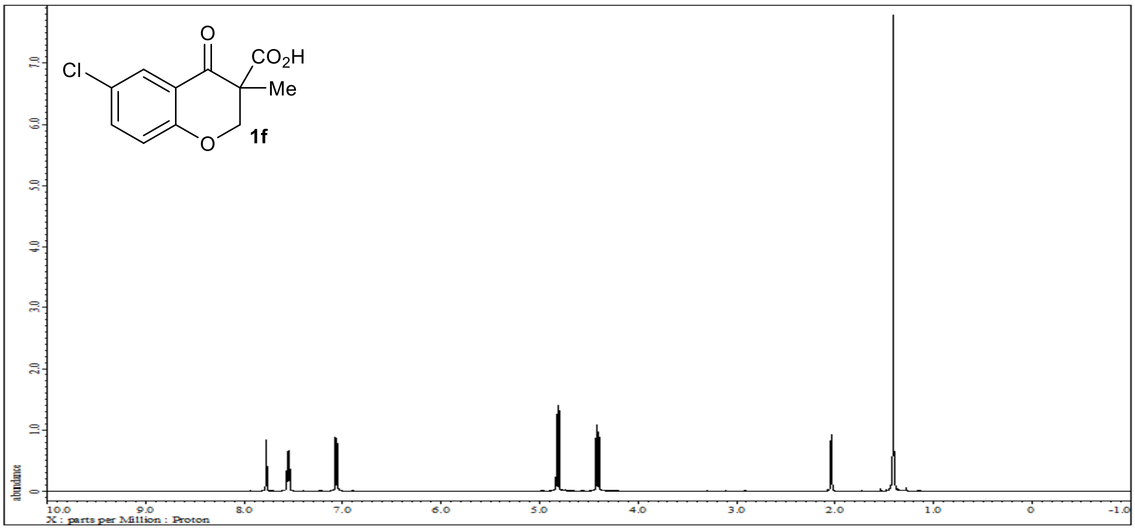

Supplementary Figure 34. ^{1}H NMR spectrum for β-ketocarboxylic acid **1f**.

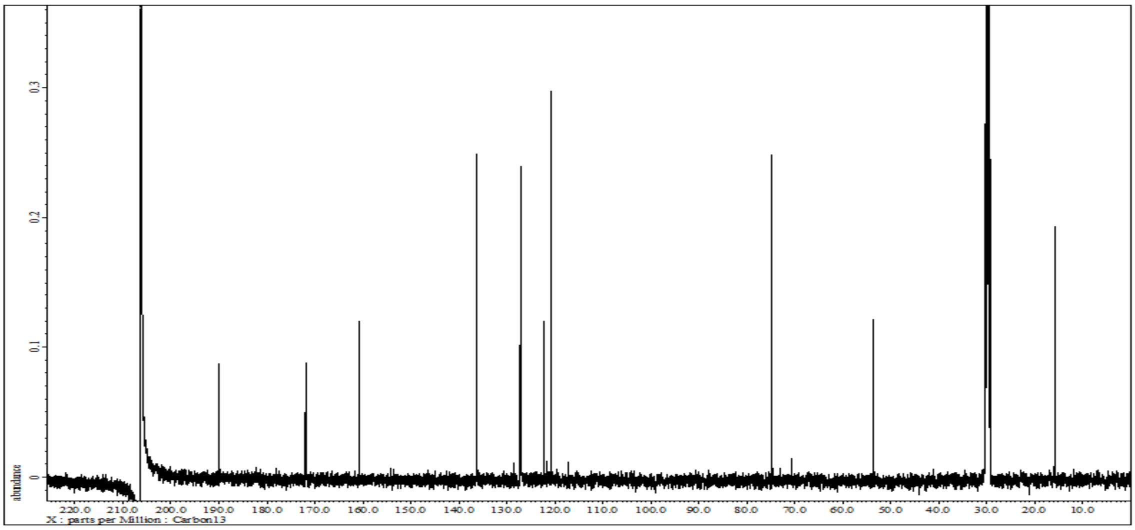

Supplementary Figure 35. ^{13}C NMR spectrum for β-ketocarboxylic acid **1f**.

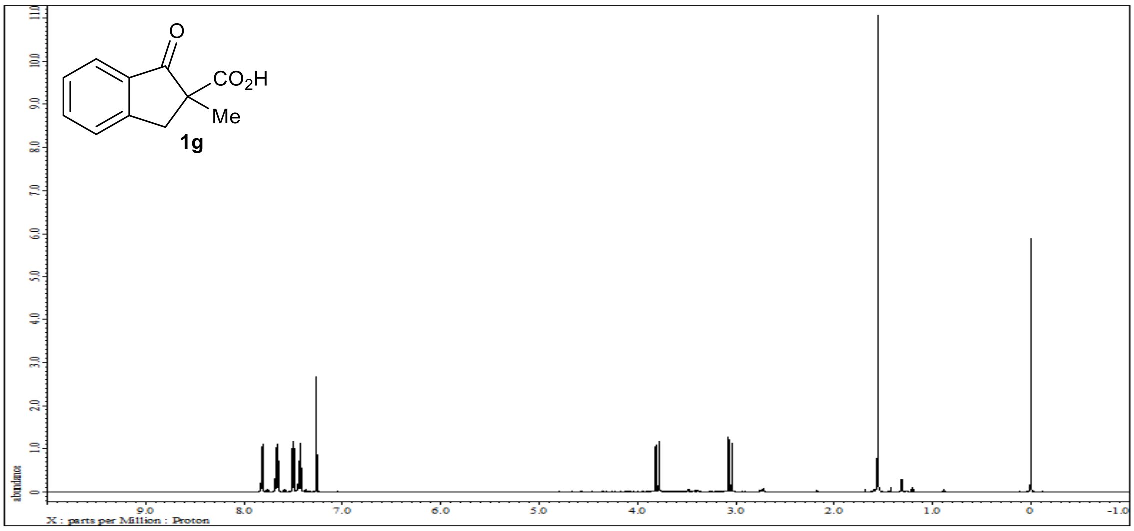

Supplementary Figure 36. ^{1}H NMR spectrum for β-ketocarboxylic acid **1g**.

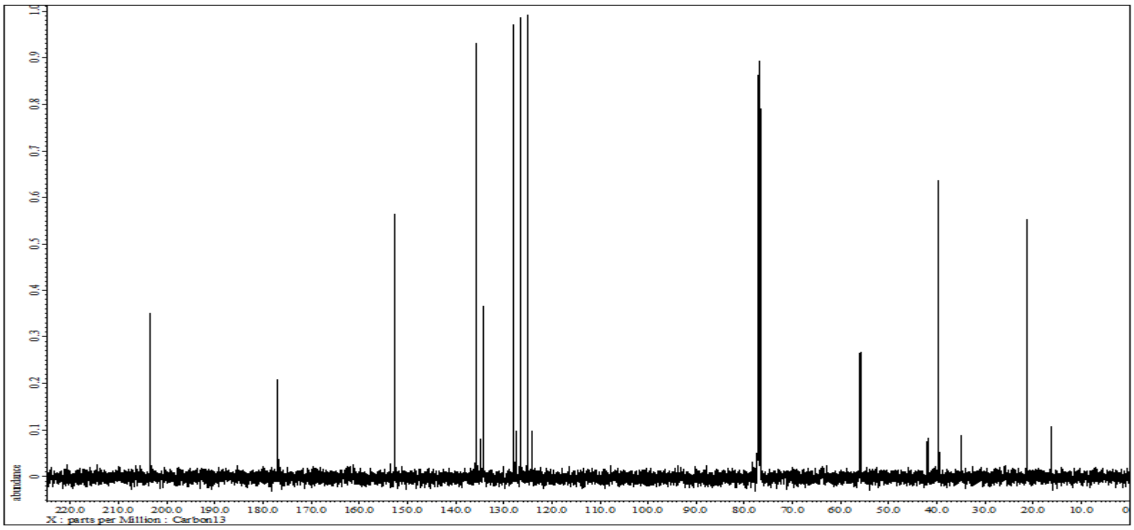

Supplementary Figure 37. ^{13}C NMR spectrum for β-ketocarboxylic acid **1g**.

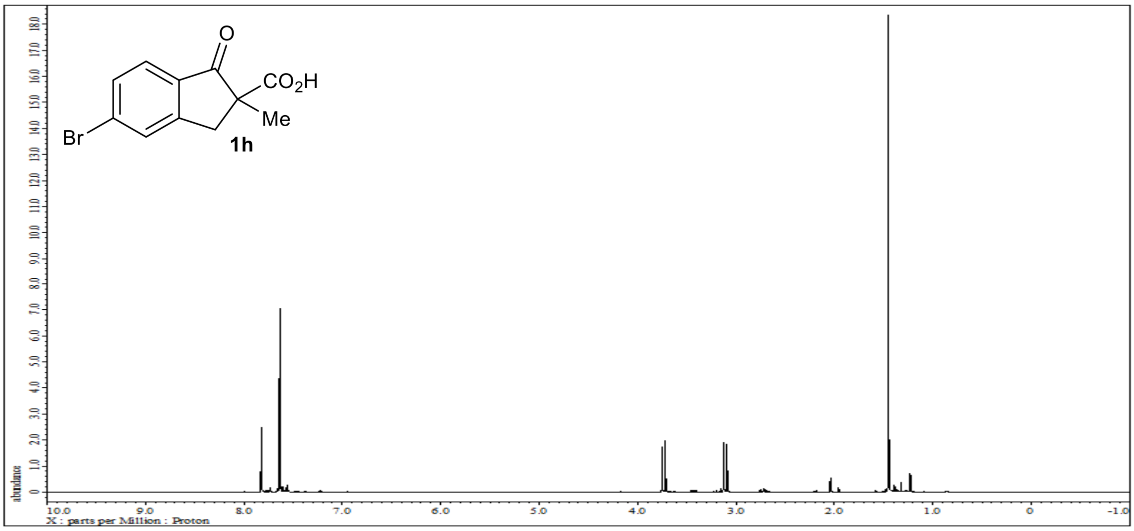

Supplementary Figure 38. ^{1}H NMR spectrum for β-ketocarboxylic acid **1h**.

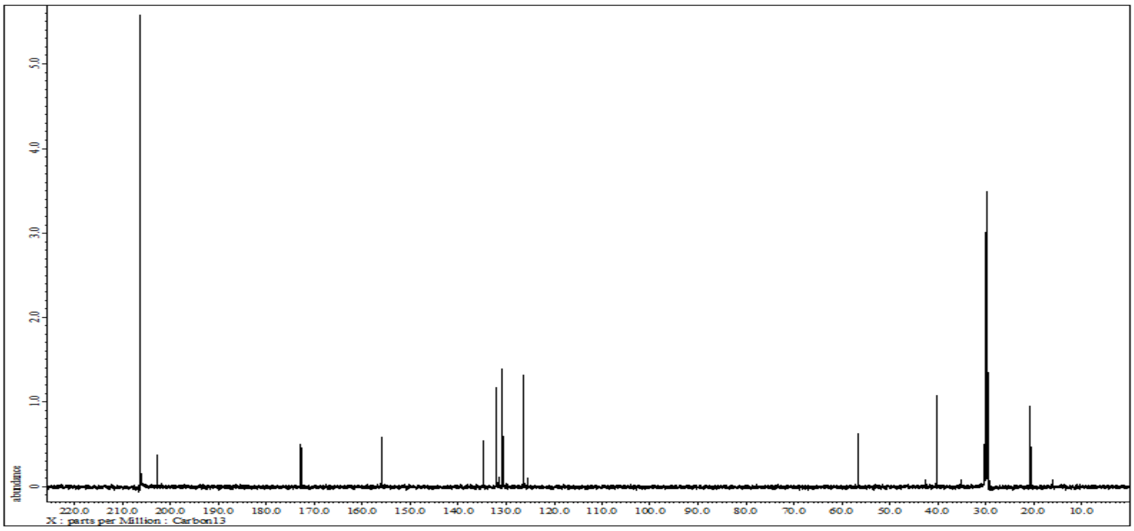

Supplementary Figure 39. ^{13}C NMR spectrum for β-ketocarboxylic acid **1h**.

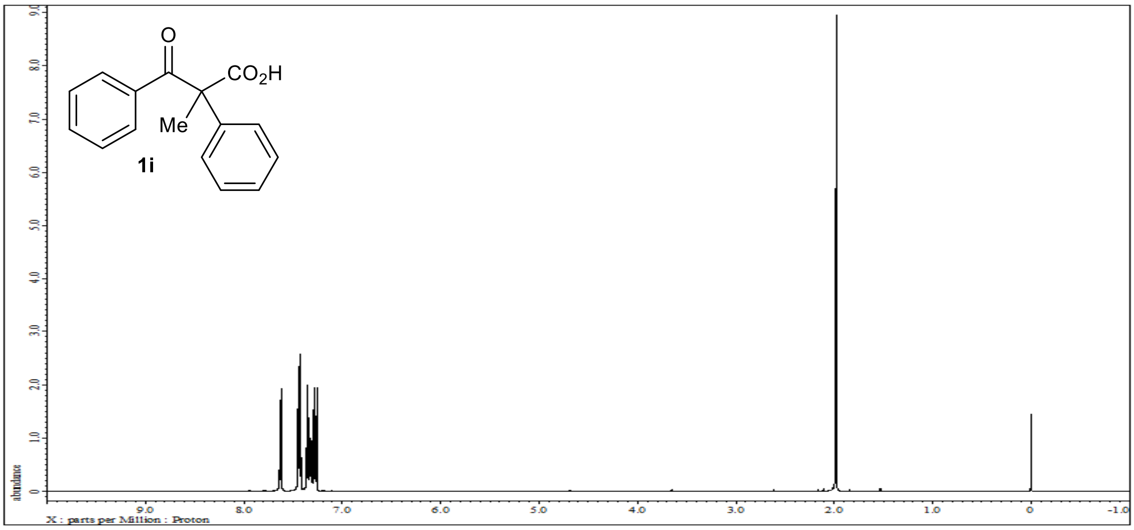

Supplementary Figure 40. ^{1}H NMR spectrum for β-ketocarboxylic acid **1i**.

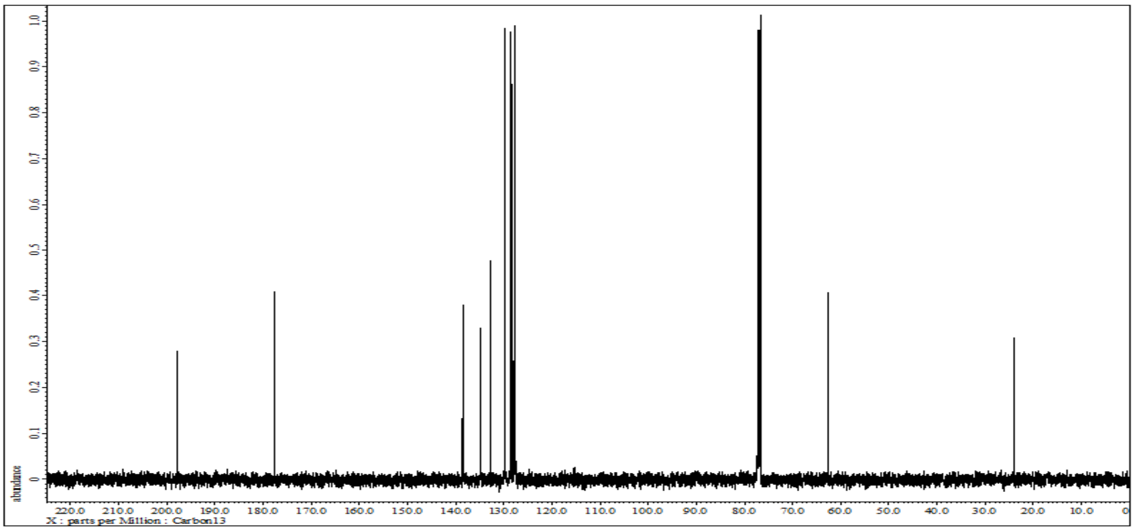

Supplementary Figure 41. ^{13}C NMR spectrum for β-ketocarboxylic acid **1i**.

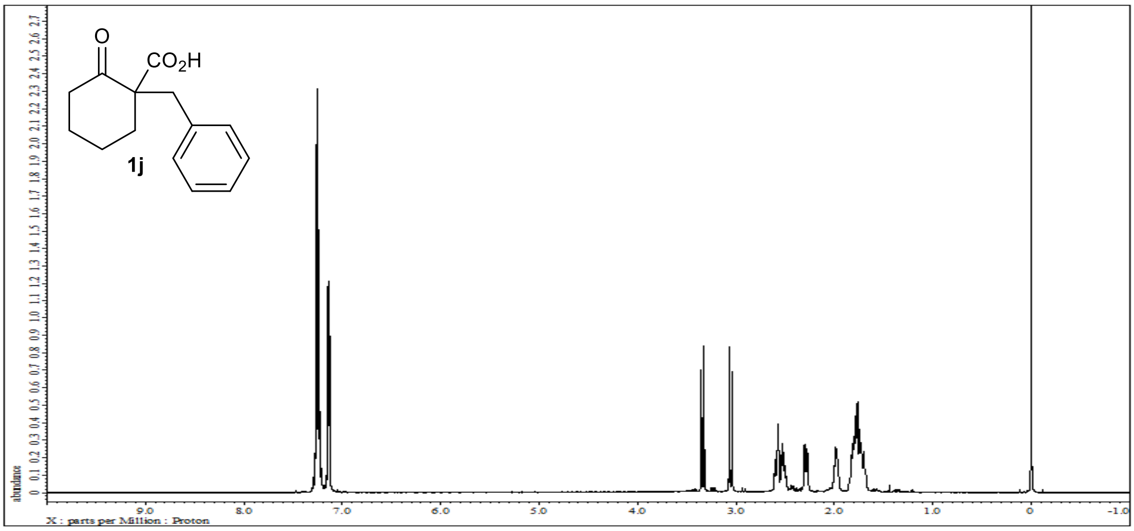

Supplementary Figure 42. ^{1}H NMR spectrum for β-ketocarboxylic acid **1j**.

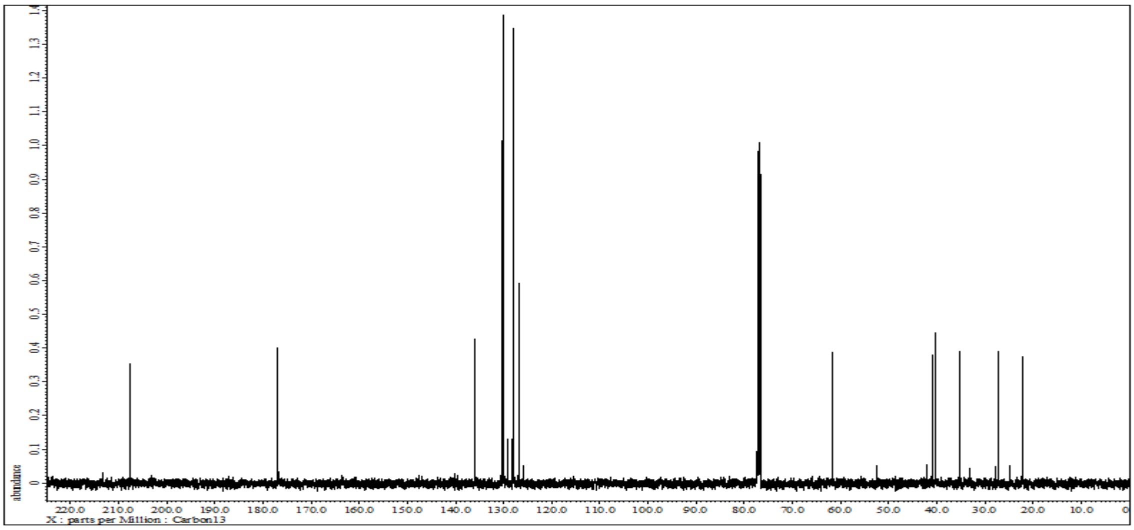

Supplementary Figure 43. ^{13}C NMR spectrum for β-ketocarboxylic acid **1j**.

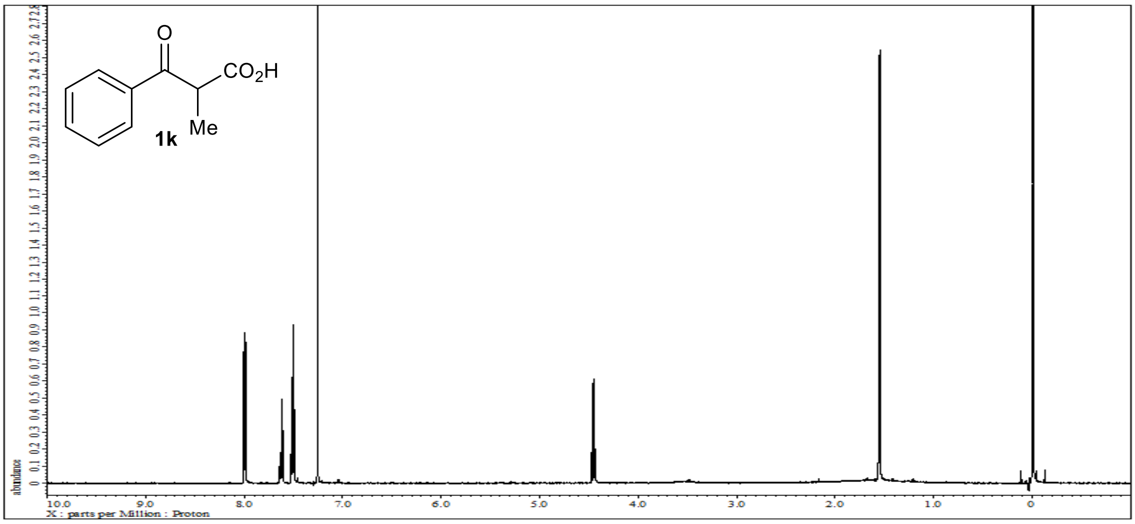

Supplementary Figure 44. ^{1}H NMR spectrum for β-ketocarboxylic acid **1k**.

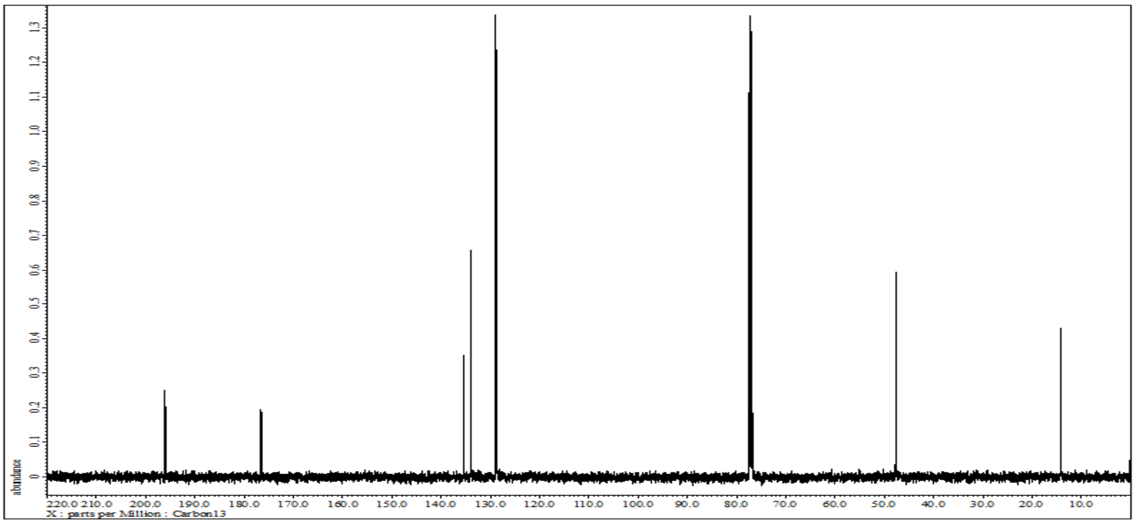

Supplementary Figure 45.^{13}C NMR spectrum for β-ketocarboxylic acid **1k**.

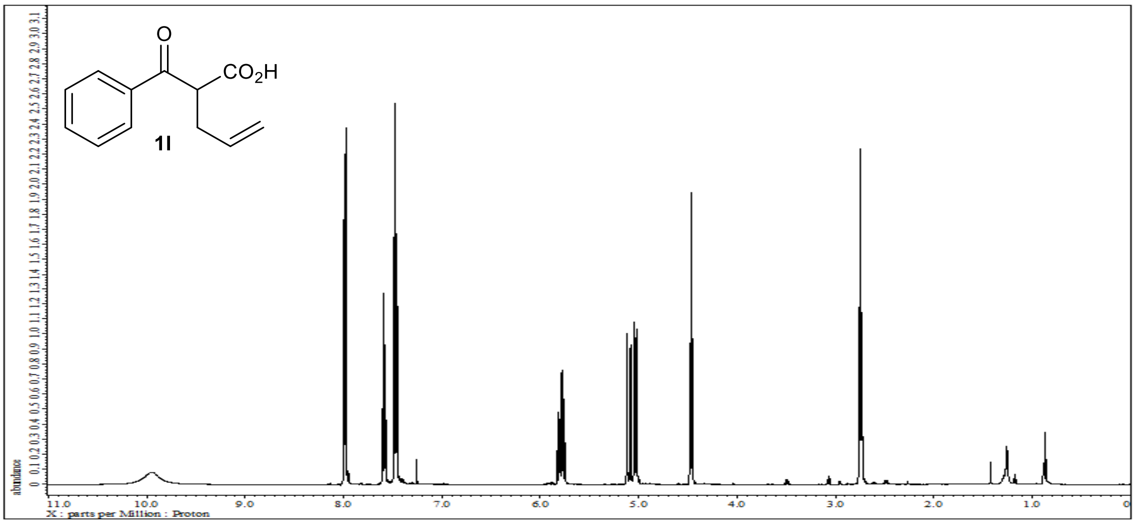

Supplementary Figure 46 ^{1}H NMR spectrum for β-ketocarboxylic acid **1l**.

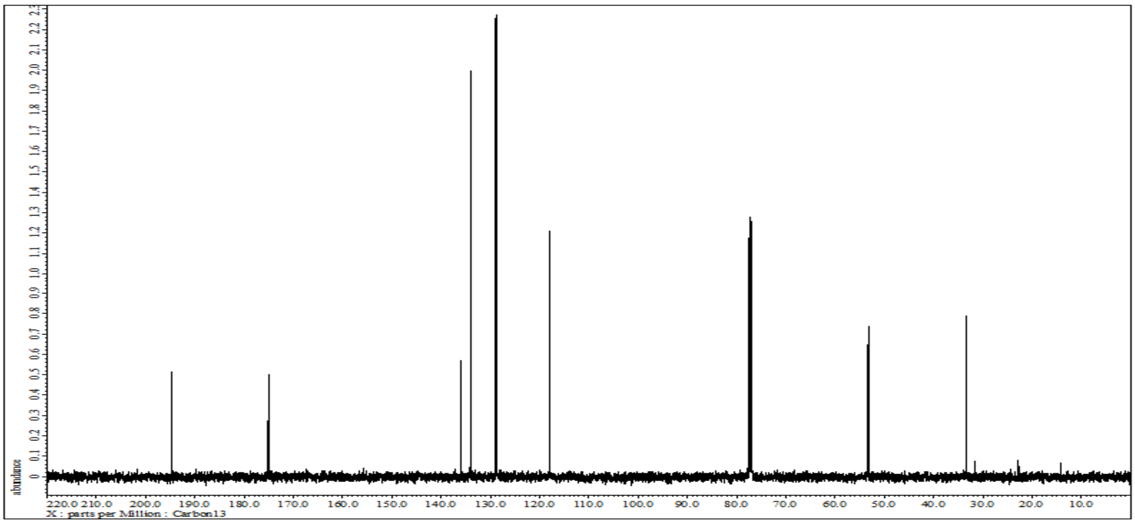

Supplementary Figure 47 ^{13}C NMR spectrum for β-ketocarboxylic acid **1l**.

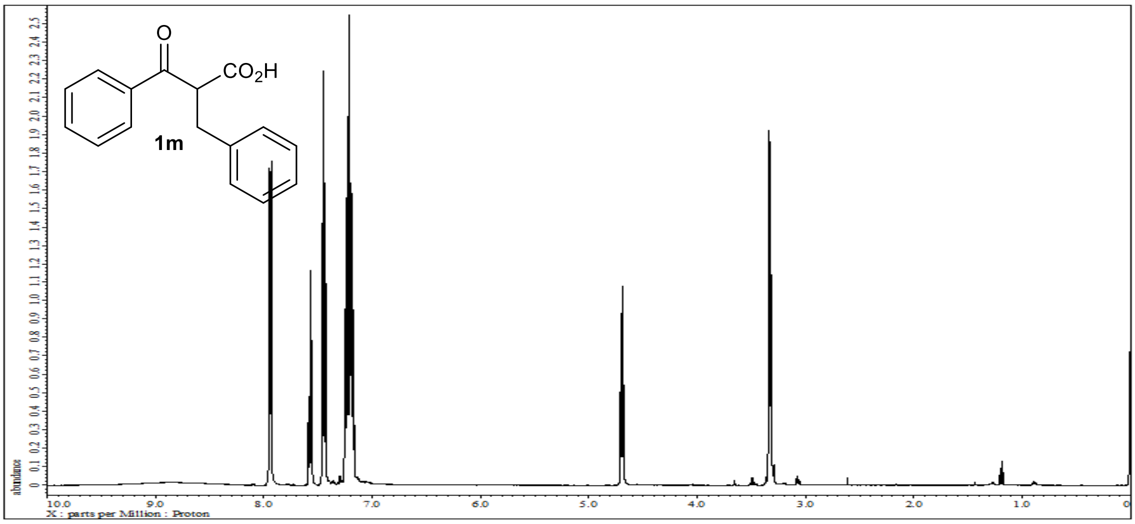

Supplementary Figure 48. ^{1}H NMR spectrum for β-ketocarboxylic acid **1m**.

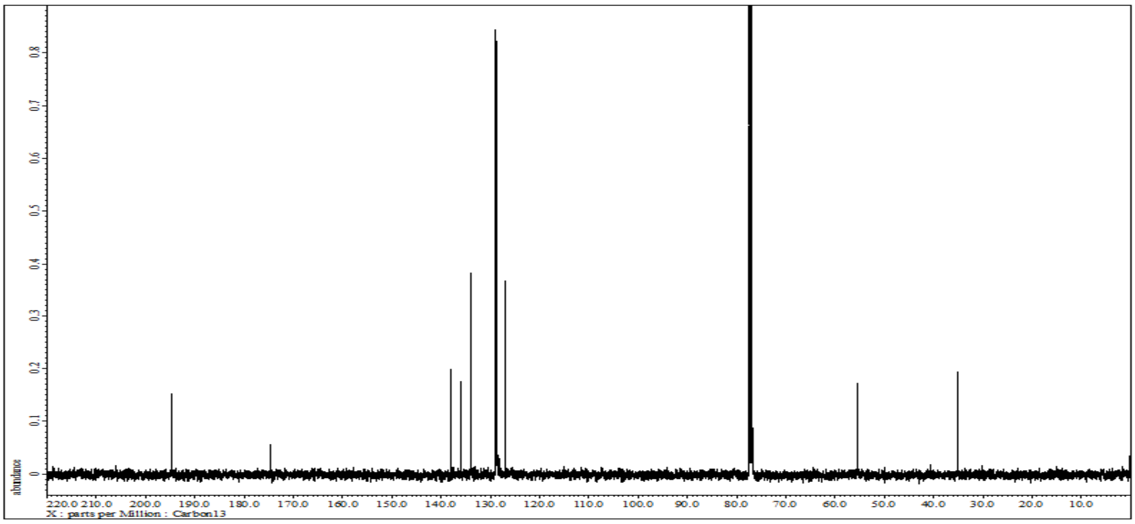

Supplementary Figure 49. ^{13}C NMR spectrum for β-ketocarboxylic acid **1m**.

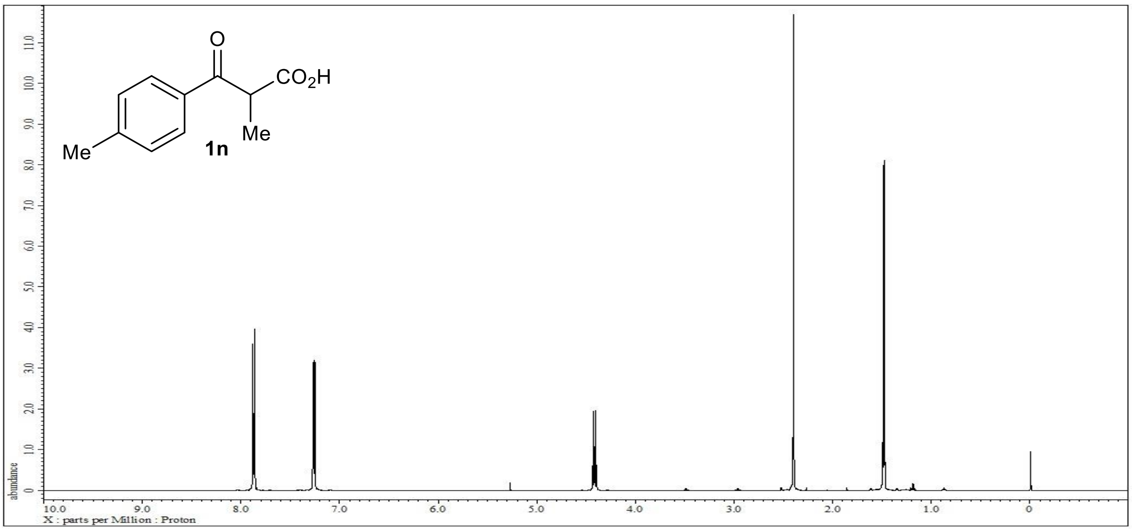

Supplementary Figure 50. ^{1}H NMR spectrum for β-ketocarboxylic acid **1n**.

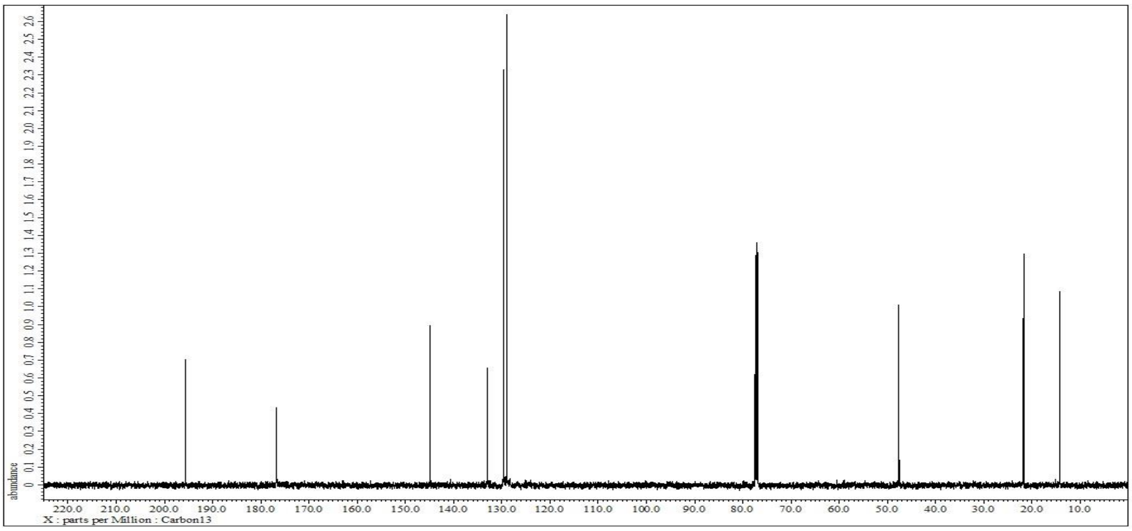

Supplementary Figure 51. ^{13}C NMR spectrum for β-ketocarboxylic acid **1n**.

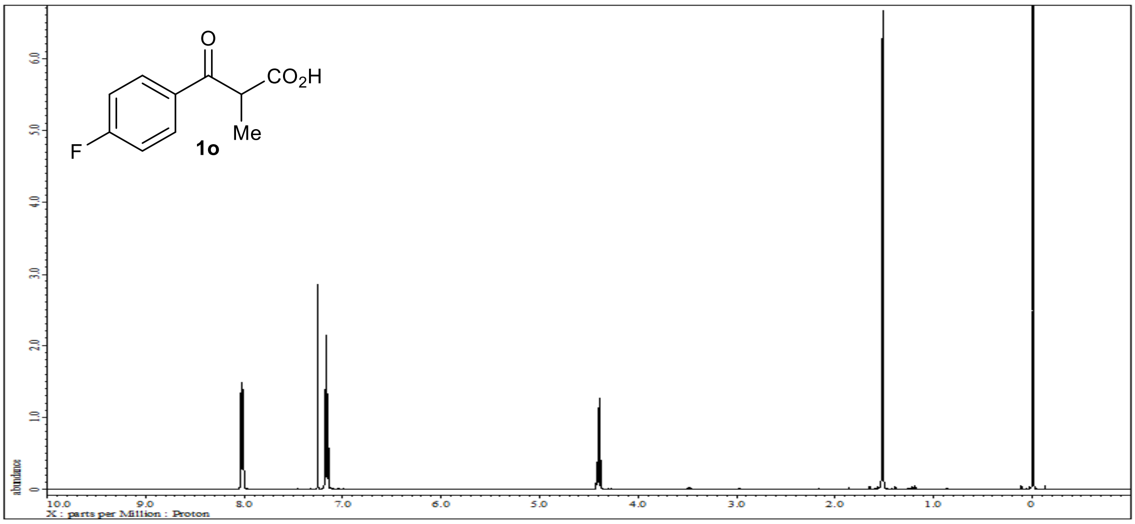

Supplementary Figure 52. ^{1}H NMR spectrum for β-ketocarboxylic acid **1o**.

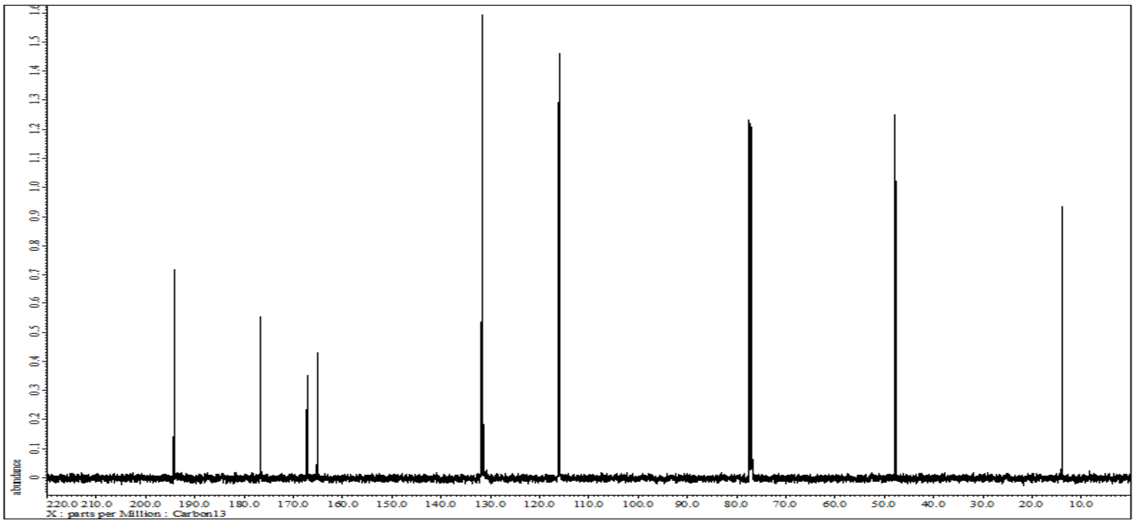

Supplementary Figure 53. ^{13}C NMR spectrum for β-ketocarboxylic acid **1o**.

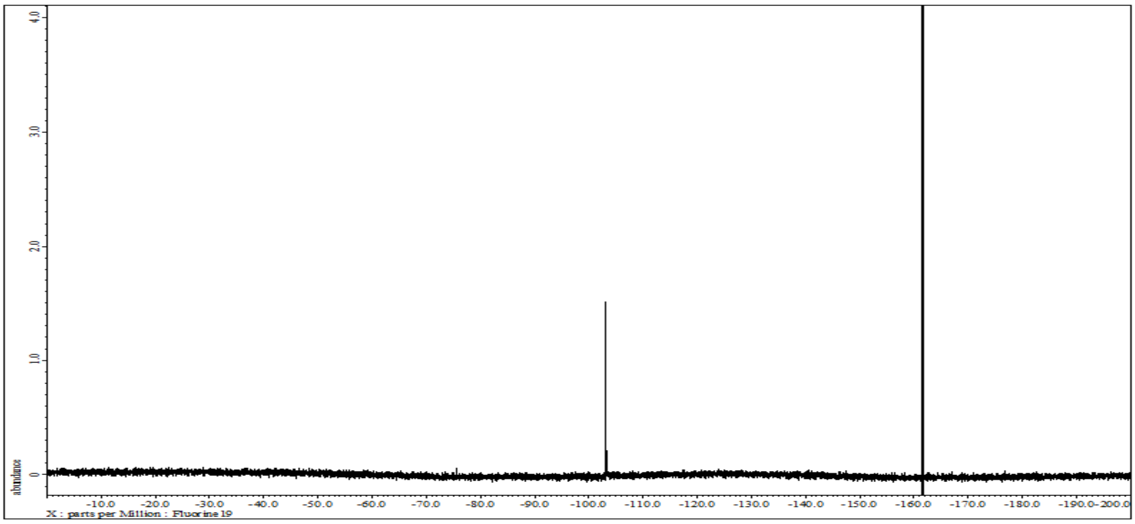

Supplementary Figure 54. ^{19}F NMR spectrum for β-ketocarboxylic acid **1o**.

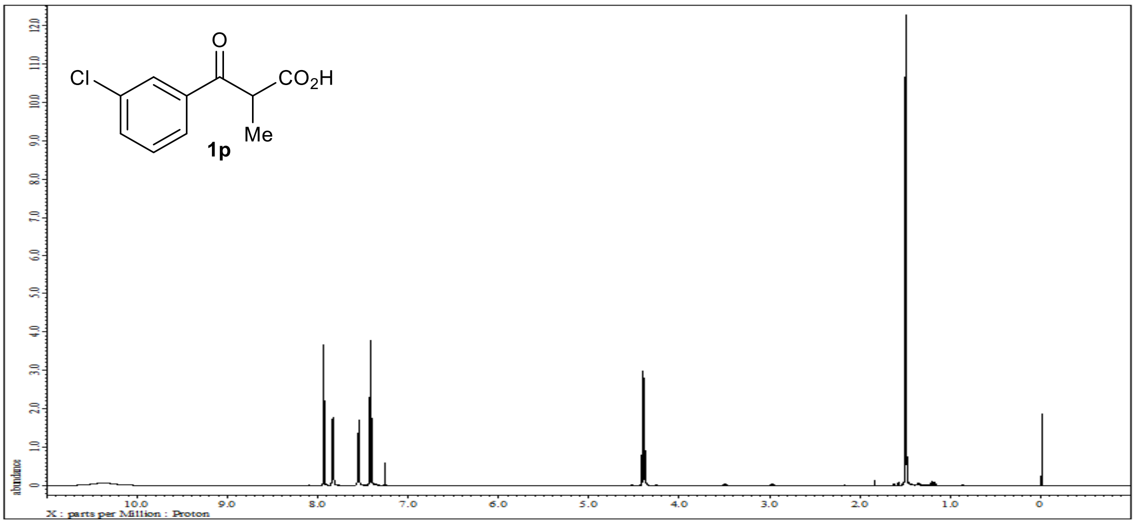

Supplementary Figure 55. ^{1}H NMR spectrum for β-ketocarboxylic acid **1p**.

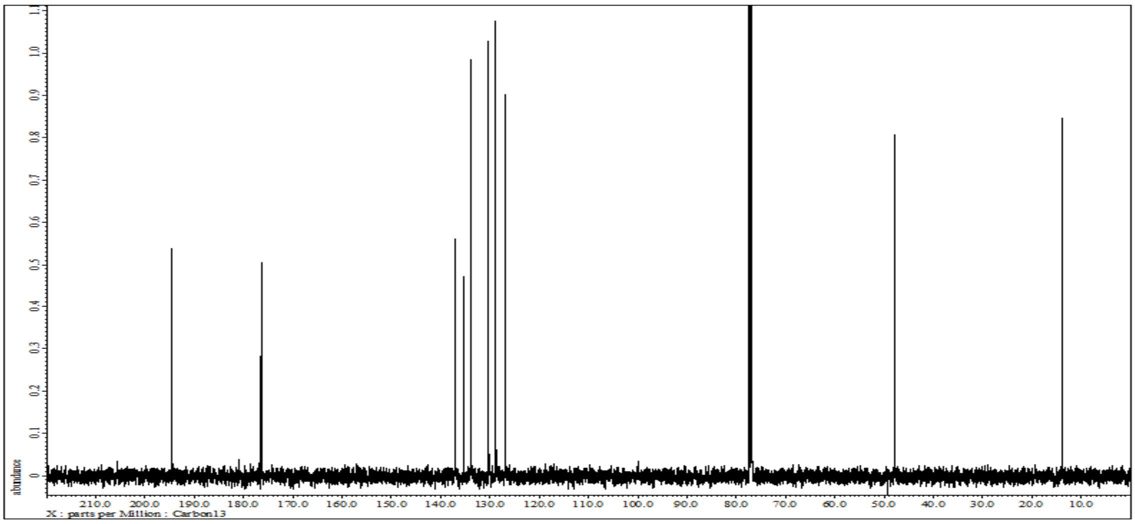

Supplementary Figure 56. ^{13}C NMR spectrum for β-ketocarboxylic acid **1p**.

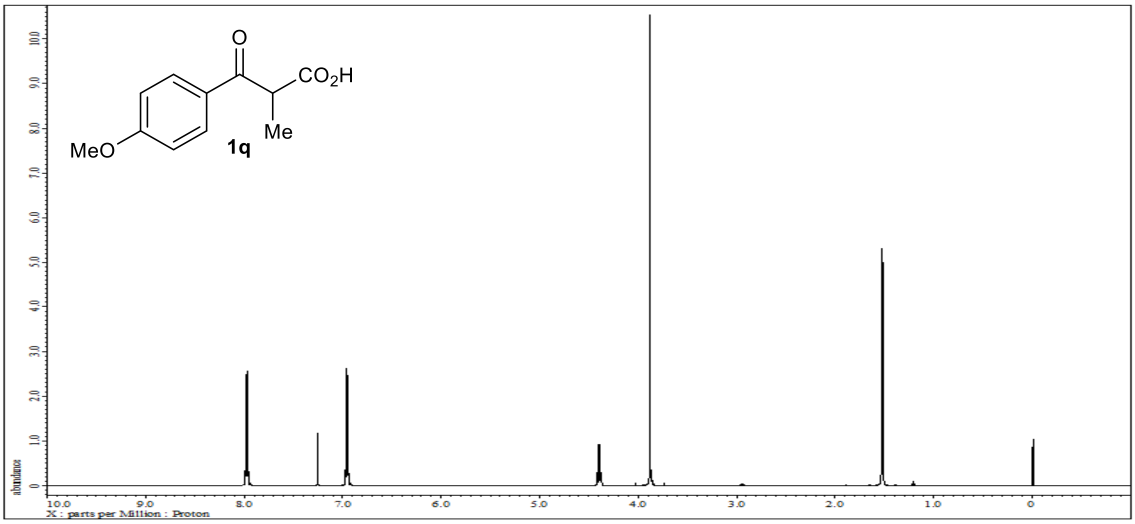

Supplementary Figure 57. ^{1}H NMR spectrum for β-ketocarboxylic acid **1q**.

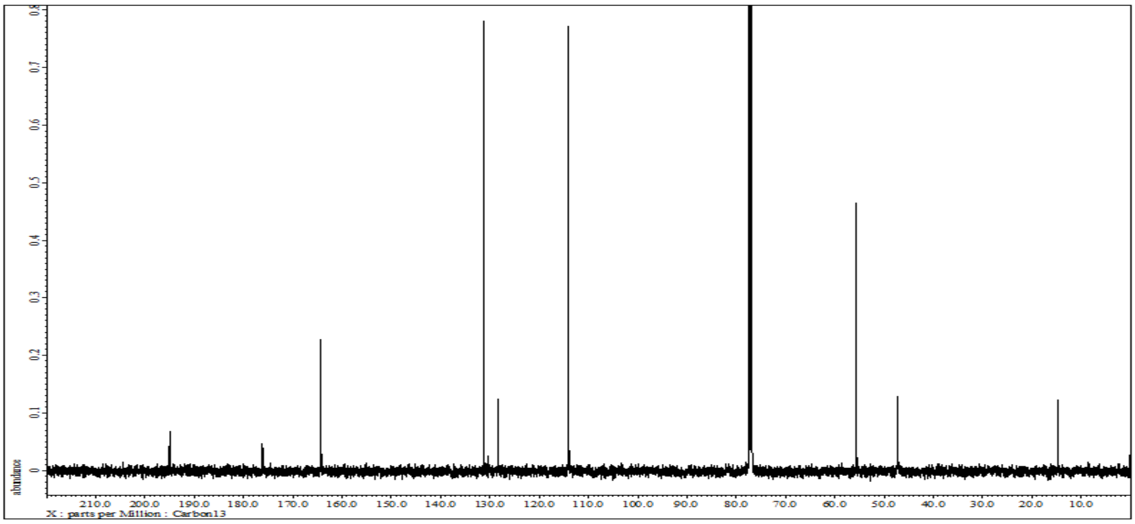

Supplementary Figure 58. ^{13}C NMR spectrum for β-ketocarboxylic acid **1q**.

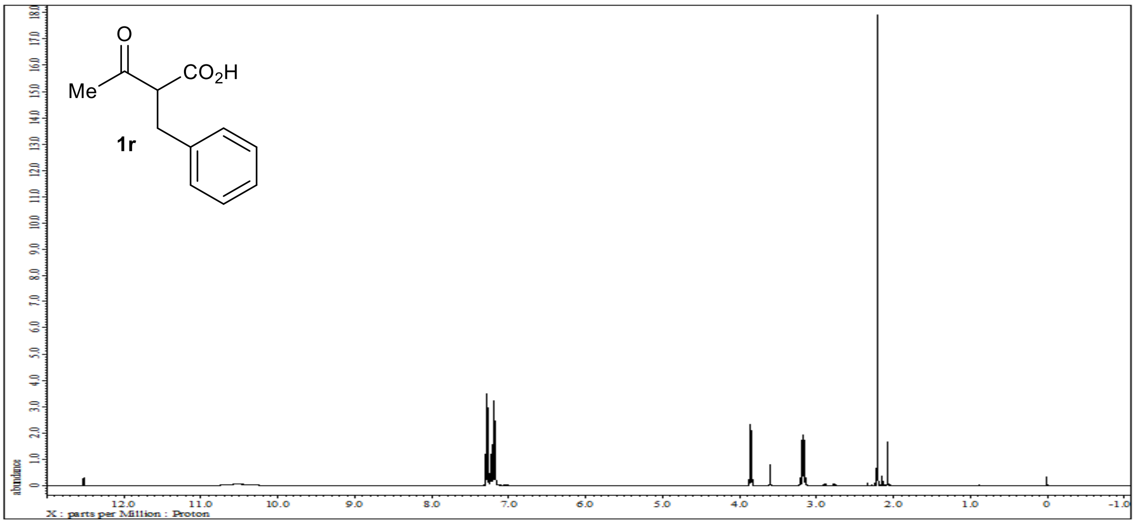

Supplementary Figure 59. ^{1}H NMR spectrum for β-ketocarboxylic acid **1r**.

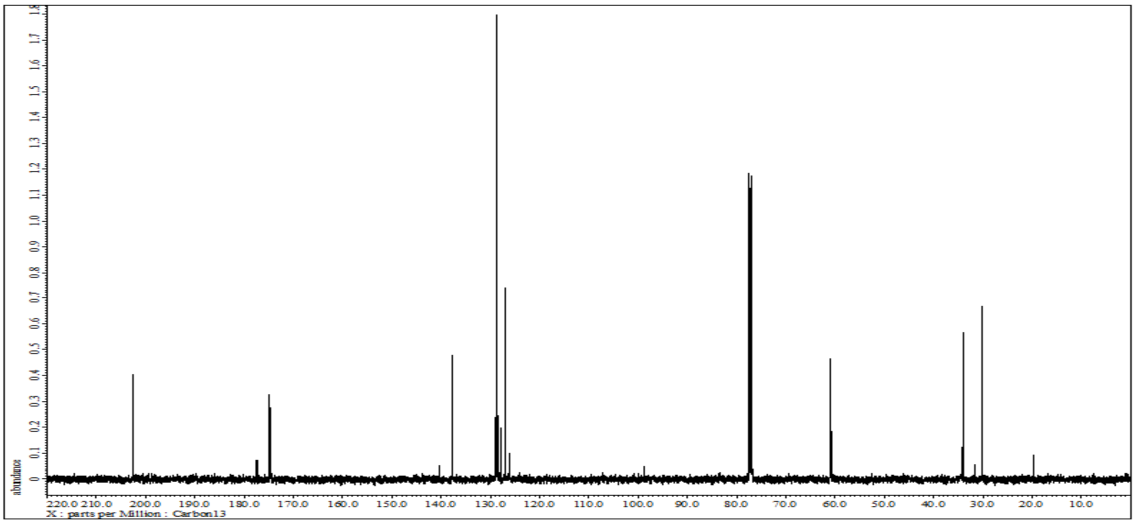

Supplementary Figure 60. ^{13}C NMR spectrum for β-ketocarboxylic acid **1r**.

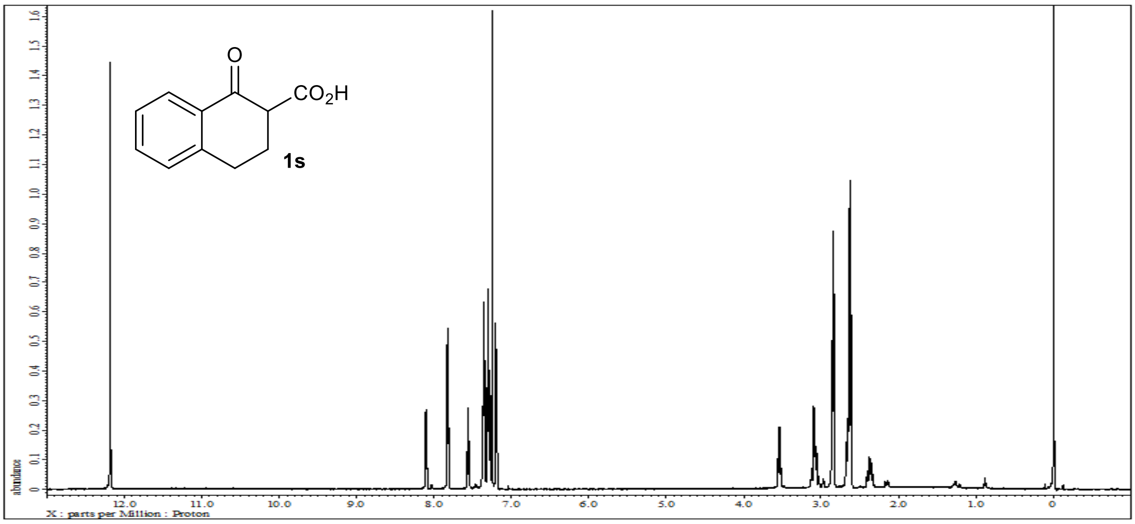

Supplementary Figure 61. ^{1}H NMR spectrum for β-ketocarboxylic acid **1s**.

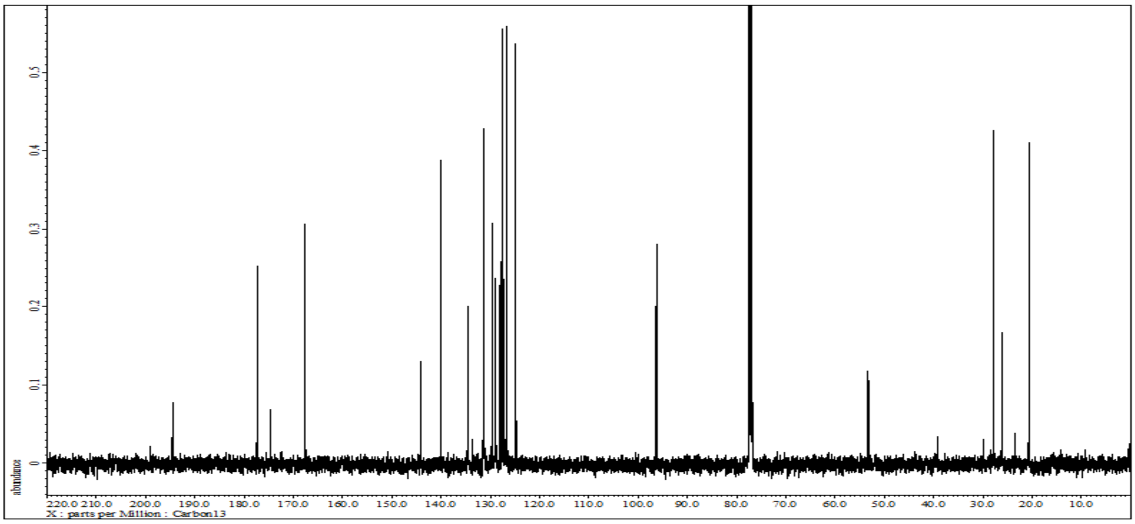

Supplementary Figure 62. ^{13}C NMR spectrum for β-ketocarboxylic acid **1s**.

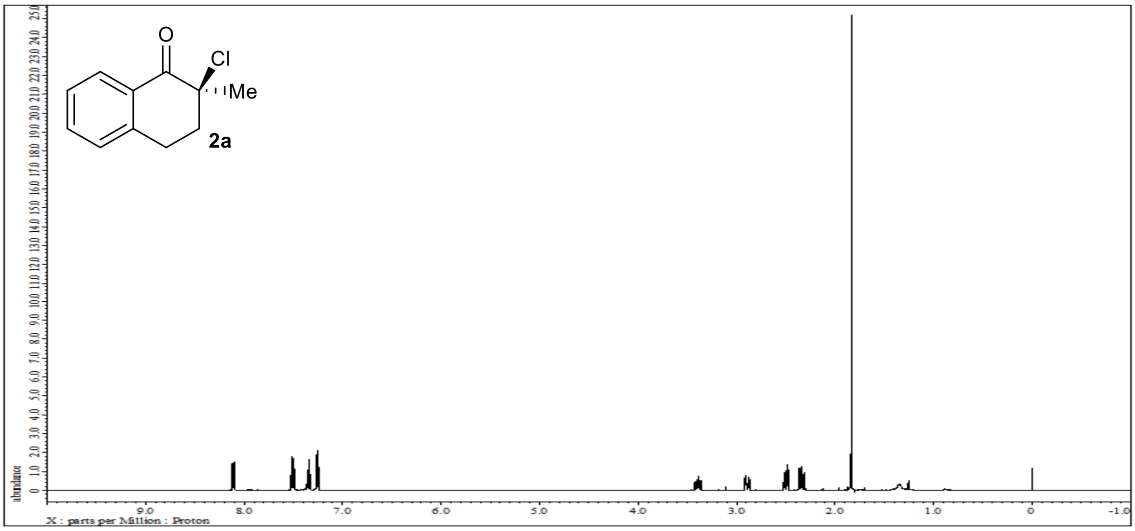

Supplementary Figure 63. ^{1}H NMR spectrum for α-chloroketone **2a**.

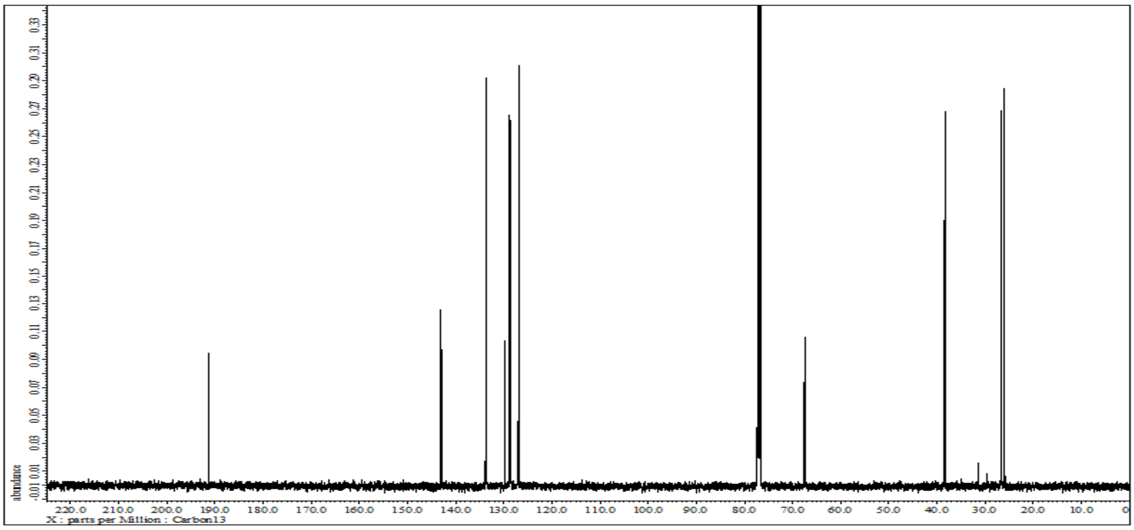

Supplementary Figure 64. ^{13}C NMR spectrum for α-chloroketone **2a**.

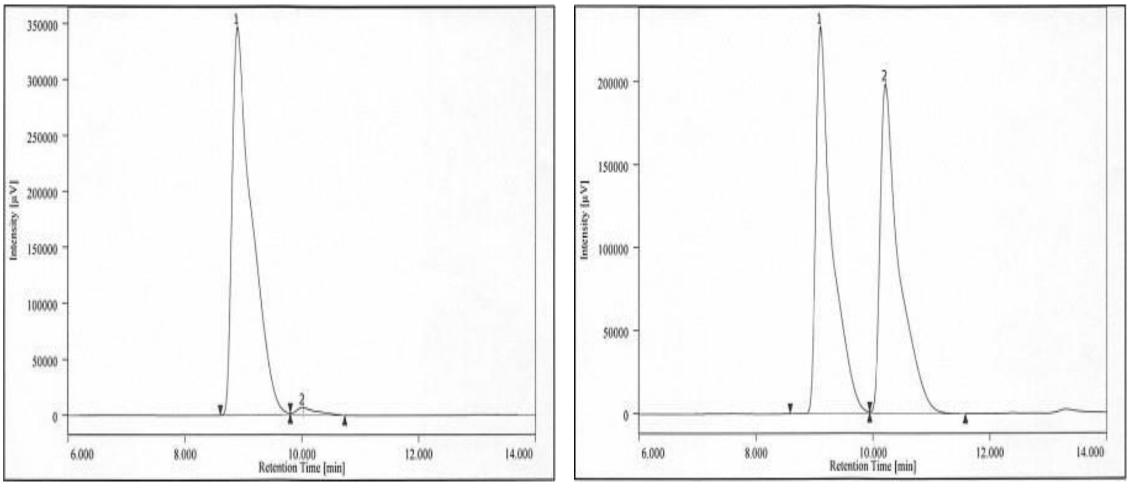

Peak	Retention Time [min]	Area [%]
1	8.9	98.001
2	10.0	1.999

Peak	Retention Time [min]	Area [%]
1	9.1	50.214
2	10.2	49.786

Supplementary Figure 65. HPLC spectra for α-chloroketone **2a**.

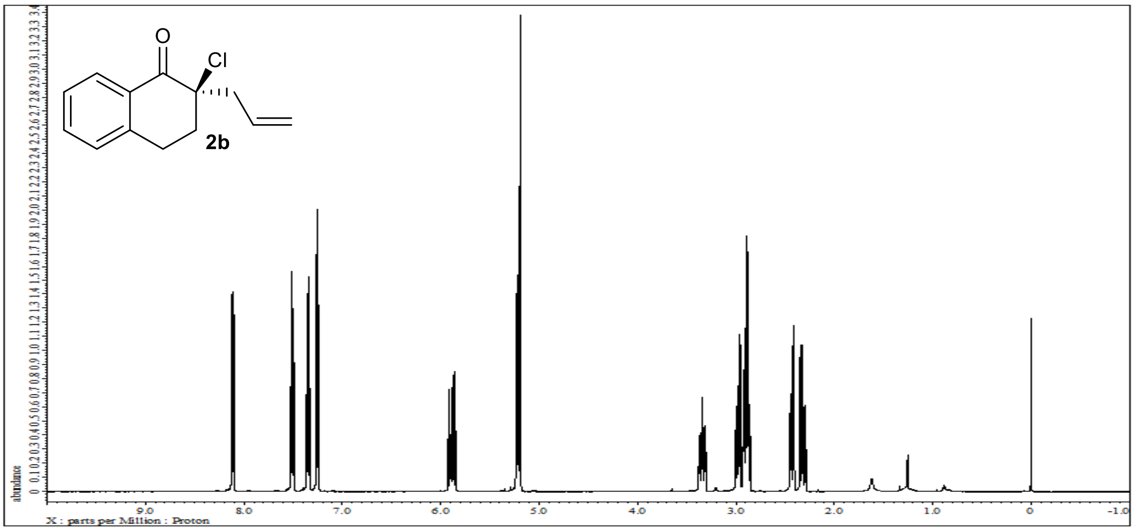

Supplementary Figure 66. ^{1}H NMR spectrum for α-chloroketone **2b**.

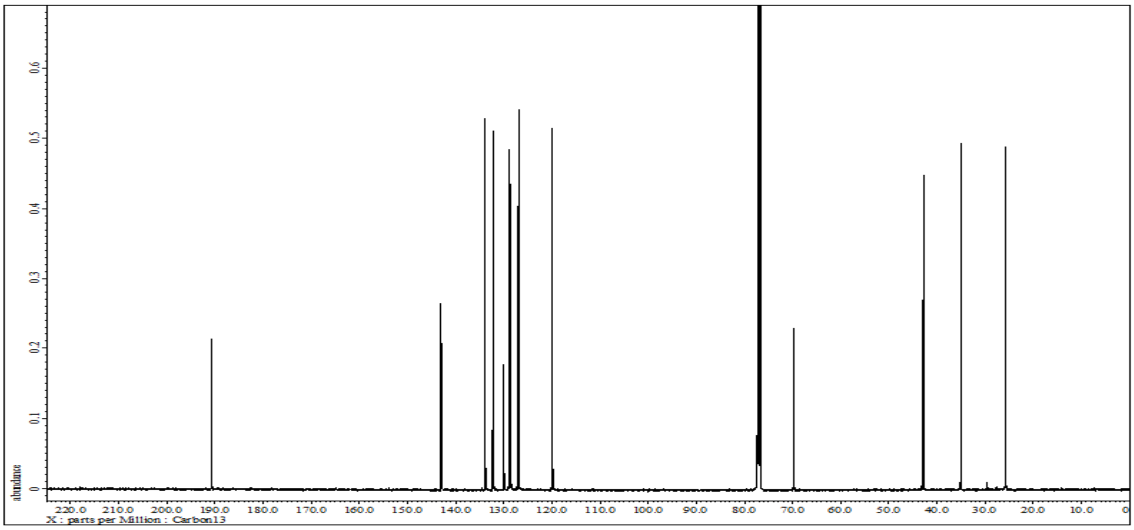

Supplementary Figure 67. ^{13}C NMR spectrum for α-chloroketone **2b**.

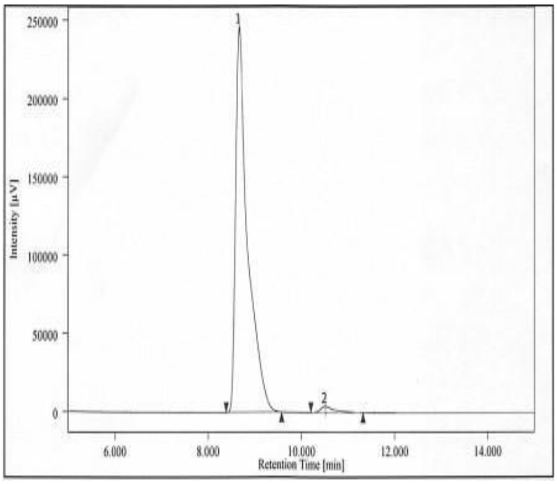

Peak	Retention Time [min]	Area [%]
1	8.7	98.122
2	10.5	1.878

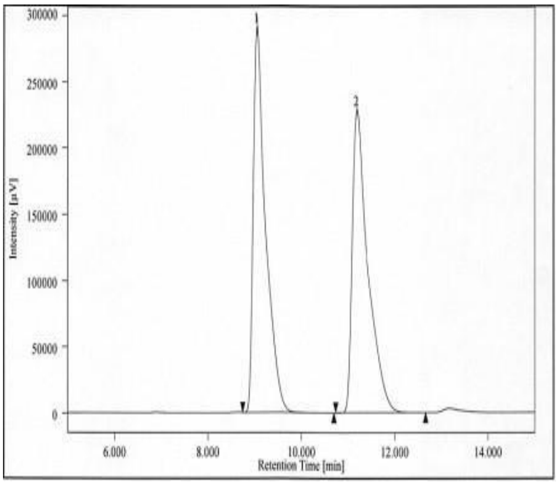

Peak	Retention Time [min]	Area [%]
1	9.1	49.934
2	11.2	50.066

Supplementary Figure 68. HPLC spectra for α-chloroketone **2b**.

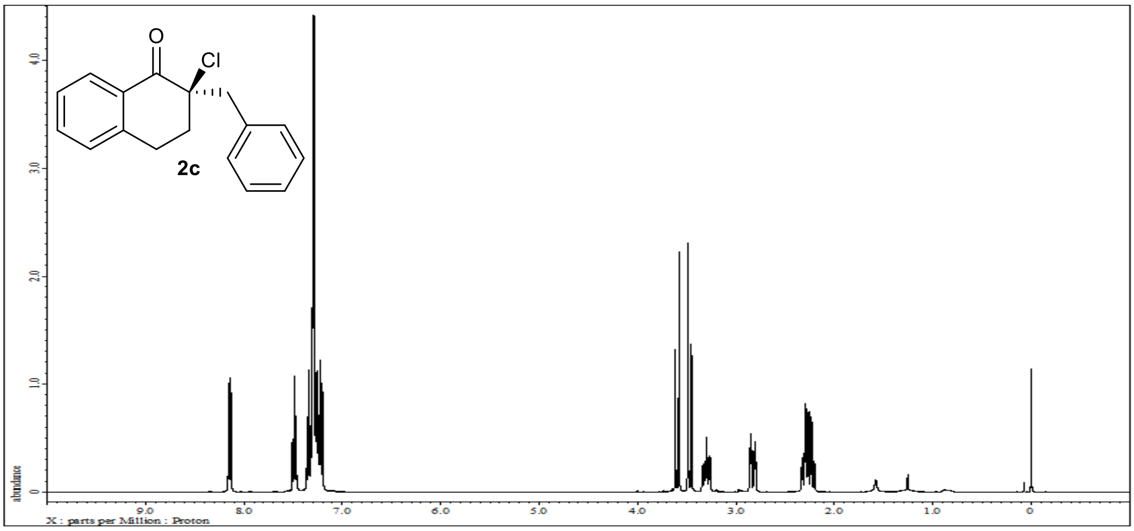

Supplementary Figure 69. ^{1}H NMR spectrum for α-chloroketone **2c**.

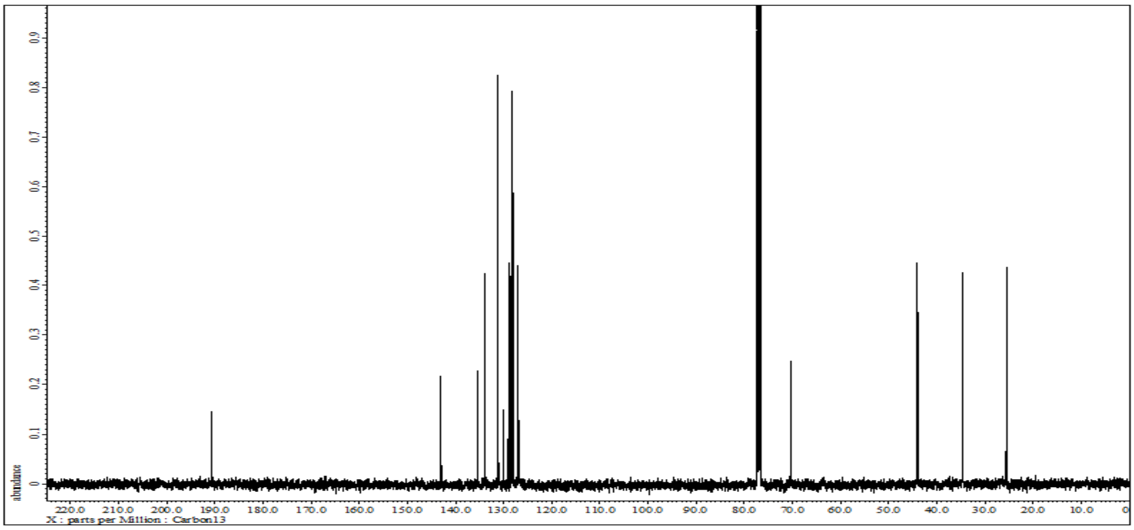

Supplementary Figure 70. ^{13}C NMR spectrum for α-chloroketone **2c**.

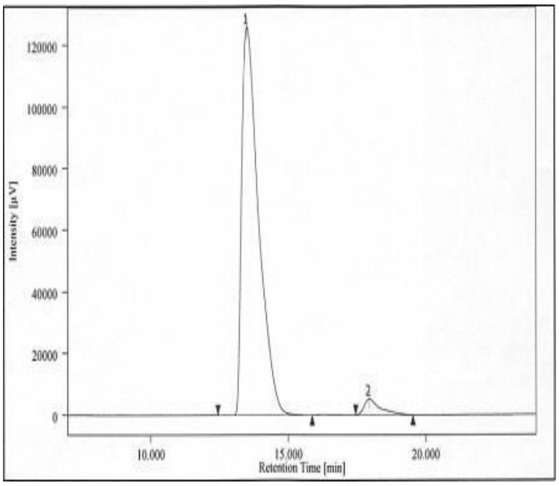

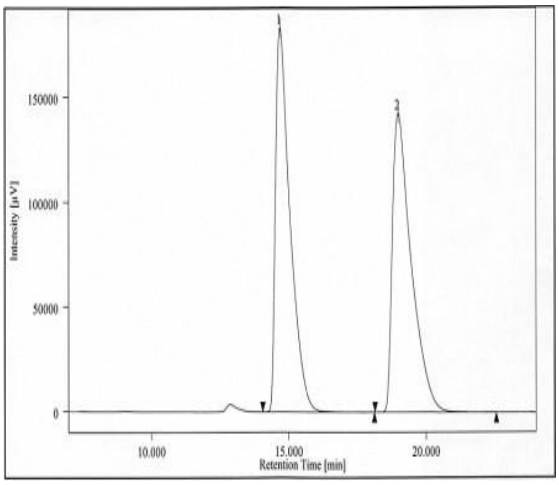

Peak	Retention Time [min]	Area [%]
1	13.5	96.295
2	17.9	3.705

Peak	Retention Time [min]	Area [%]
1	14.7	49.998
2	19.0	50.002

Supplementary Figure 71. HPLC spectra for α-chloroketone **2c**.

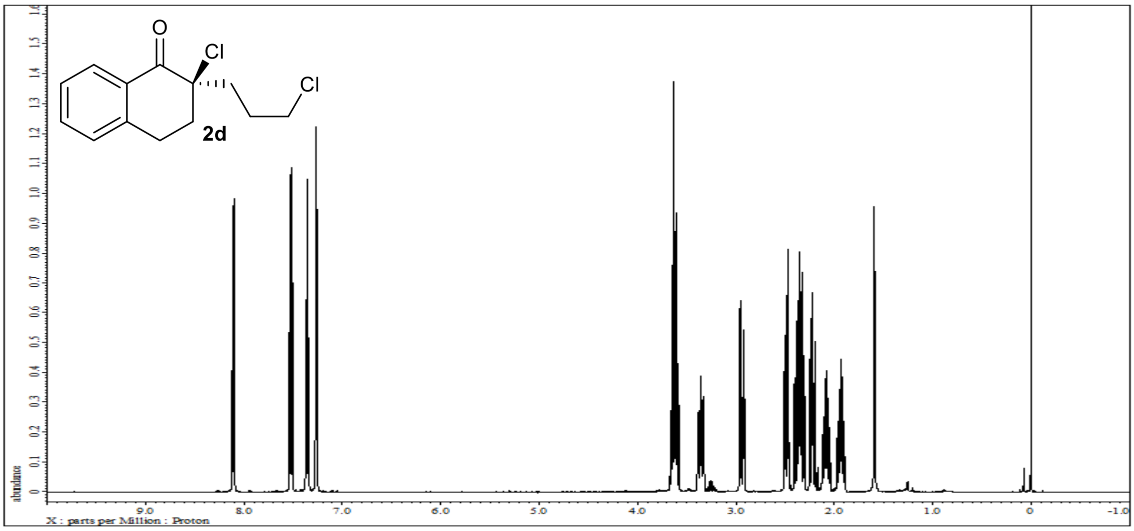

Supplementary Figure 72. ^{1}H NMR spectrum for α-chloroketone **2d**.

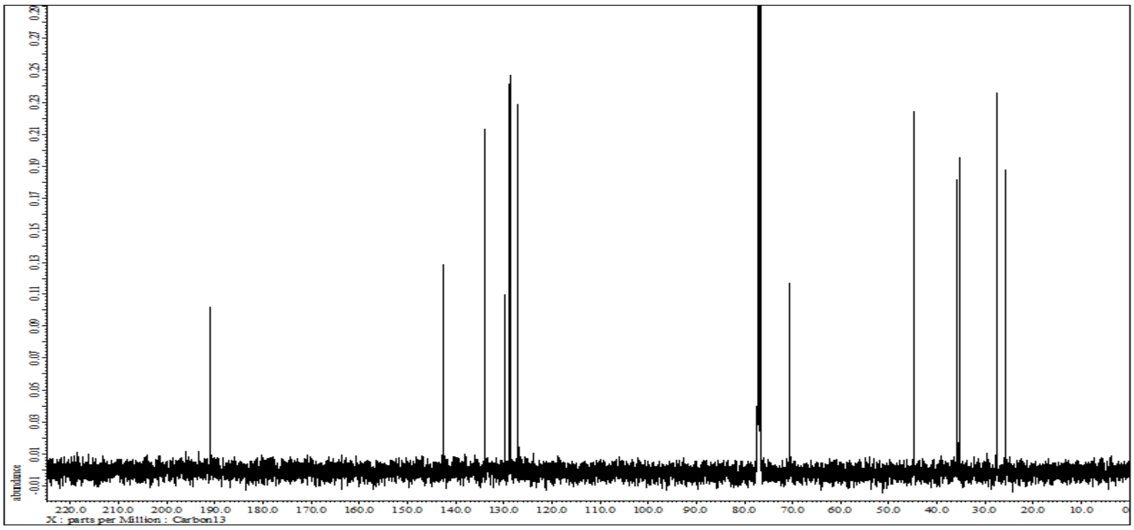

Supplementary Figure 73. ^{13}C NMR spectrum for α-chloroketone **2d**.

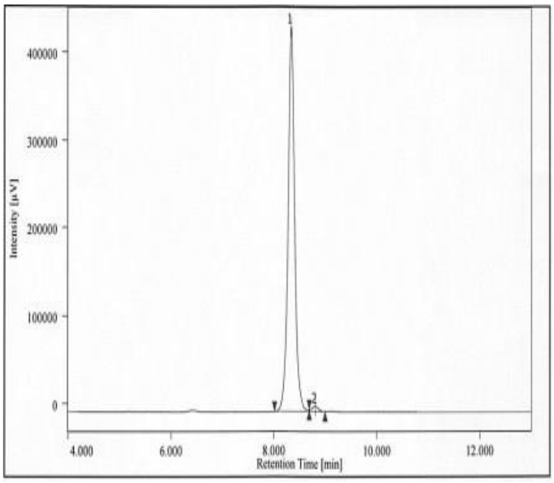

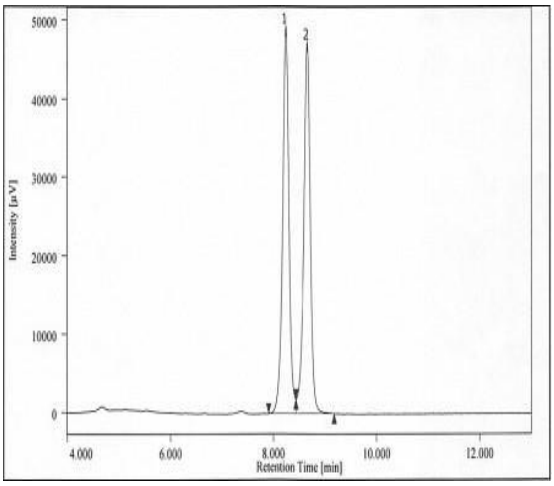

Peak	Retention Time [min]	Area [%]
1	8.3	98.688
2	8.8	1.312

Peak	Retention Time [min]	Area [%]
1	8.2	49.839
2	8.7	50.161

Supplementary Figure 74. HPLC spectra for α-chloroketone **2d**.

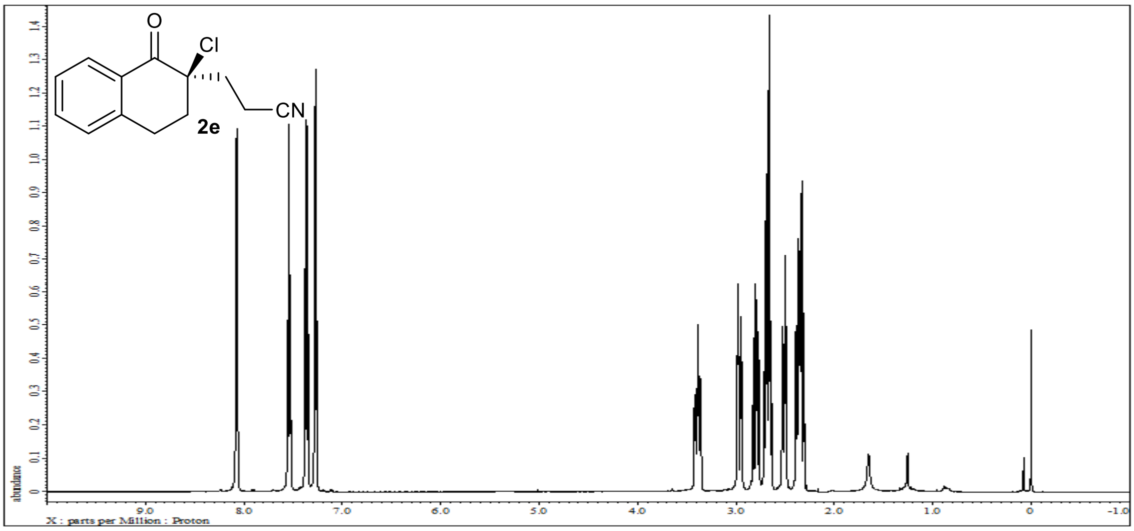

Supplementary Figure 75. ^{1}H NMR spectrum for α-chloroketone **2e**.

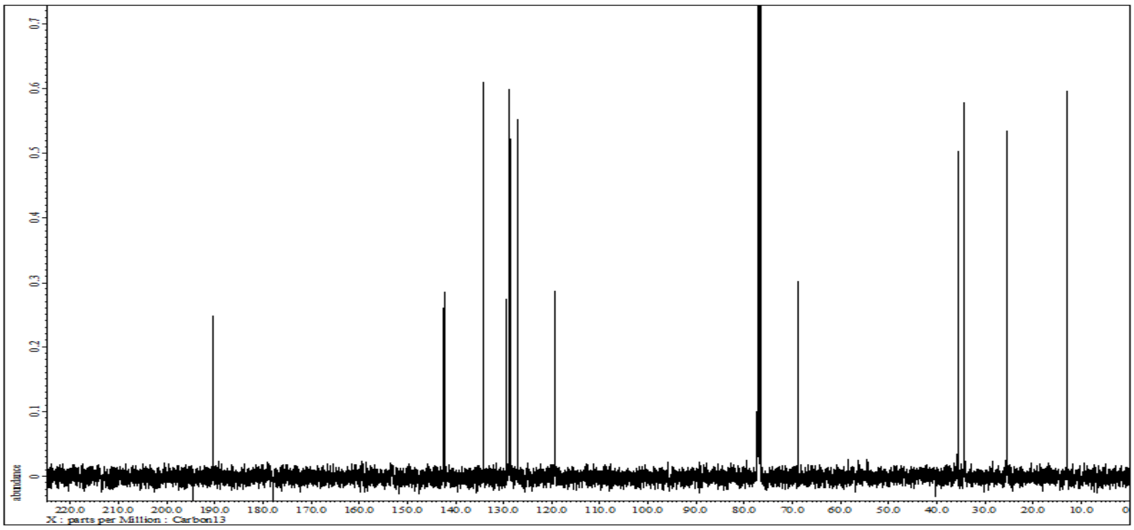

Supplementary Figure 76. ^{13}C NMR spectrum for α-chloroketone **2e**.

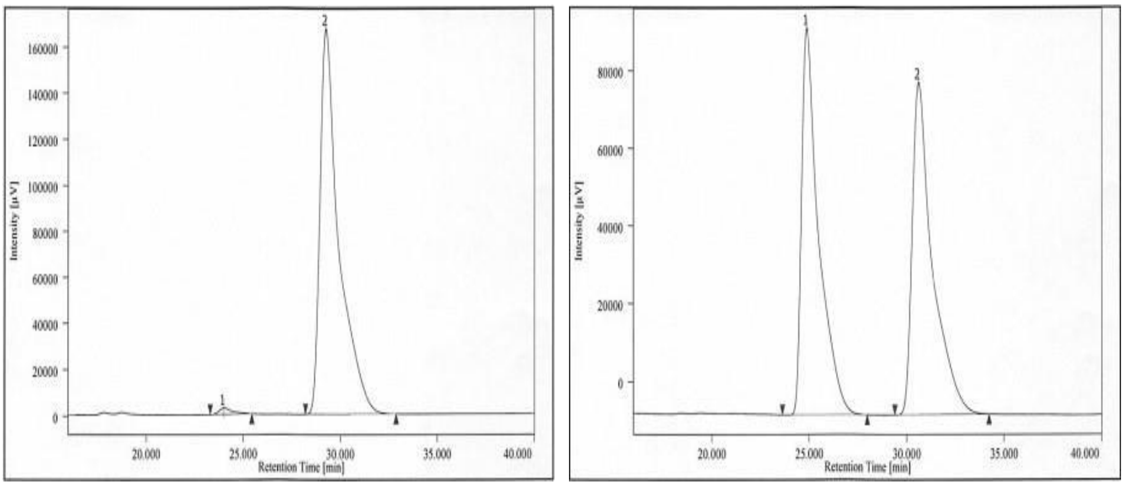

Peak	Retention Time [min]	Area [%]
1	24.0	1.179
2	29.3	98.821

Peak	Retention Time [min]	Area [%]
1	24.9	49.892
2	30.6	50.108

Supplementary Figure 77. HPLC spectra for α-chloroketone **2e**.

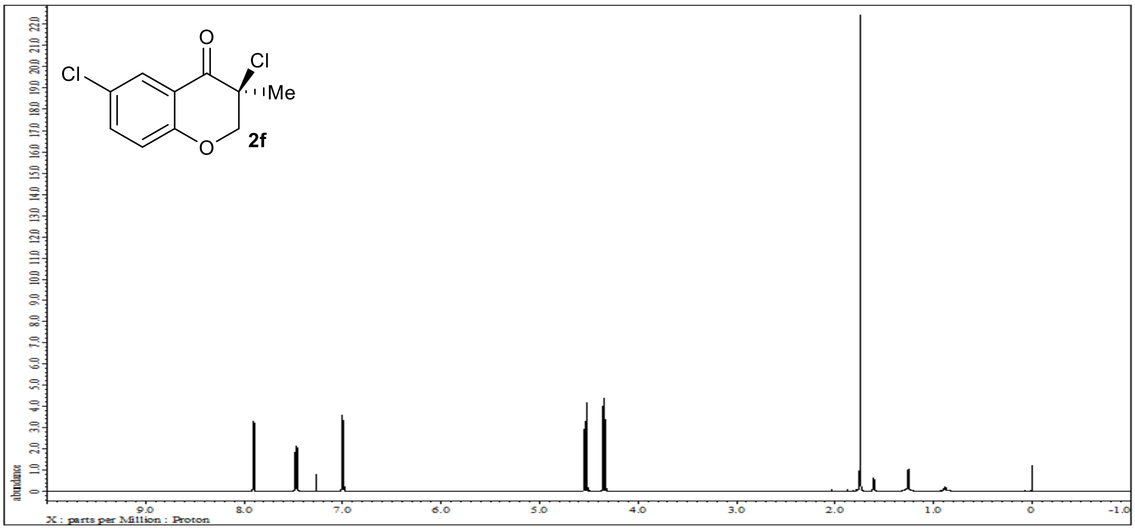

Supplementary Figure 78. ^{1}H NMR spectrum for α-chloroketone **2f**.

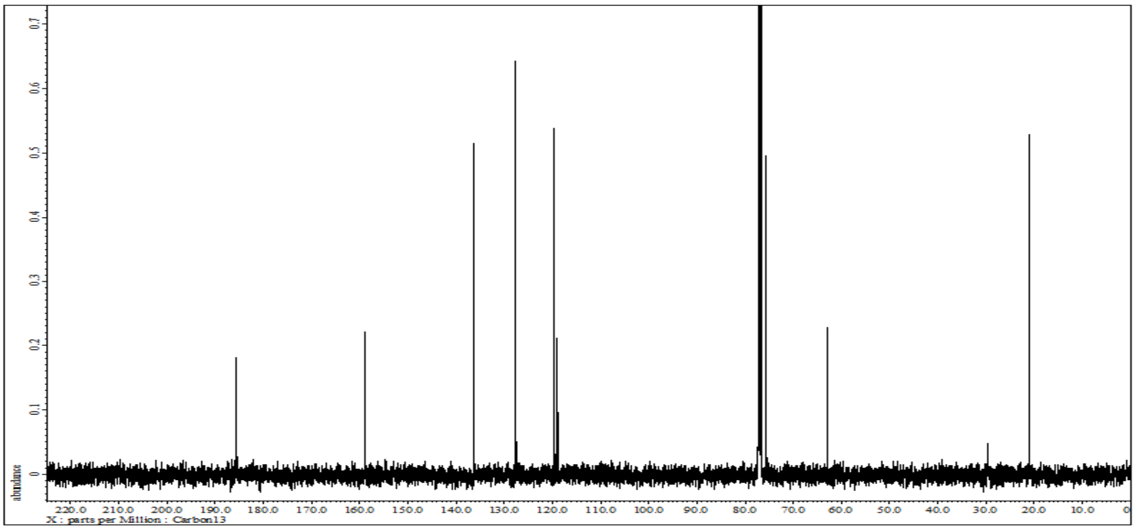

Supplementary Figure 79. ^{13}C NMR spectrum for α-chloroketone **2f**.

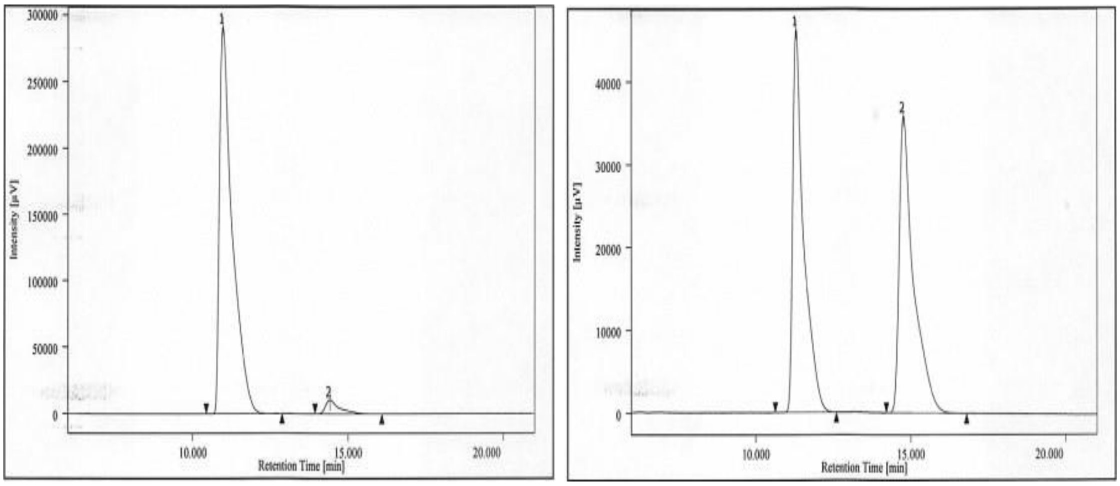

Peak	Retention Time [min]	Area [%]
1	11.0	96.249
2	14.4	3.751

Peak	Retention Time [min]	Area [%]
1	11.3	49.965
2	14.8	50.035

Supplementary Figure 80. HPLC spectra for α-chloroketone **2f**.

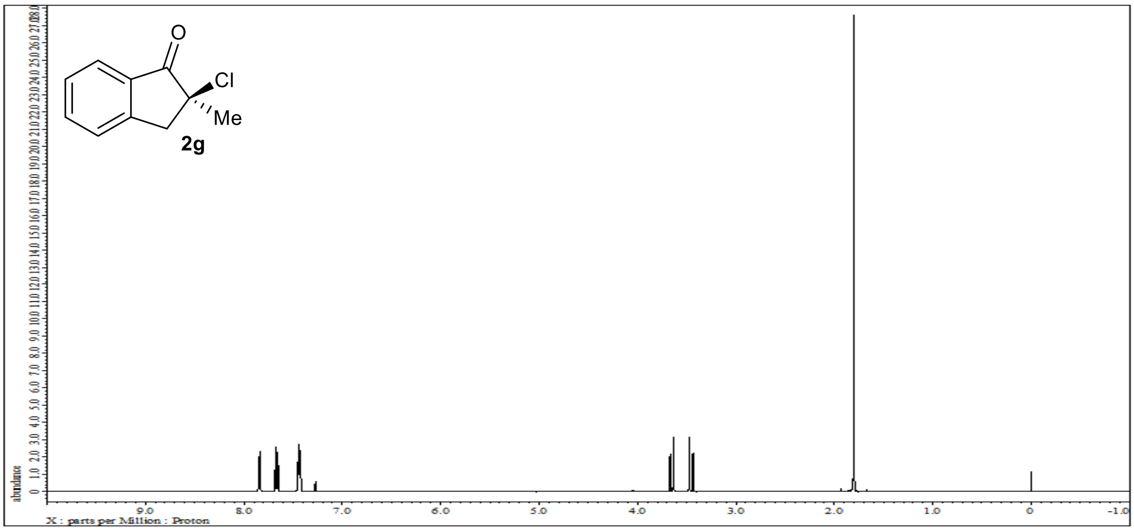

Supplementary Figure 81. ^{1}H NMR spectrum for α-chloroketone **2g**.

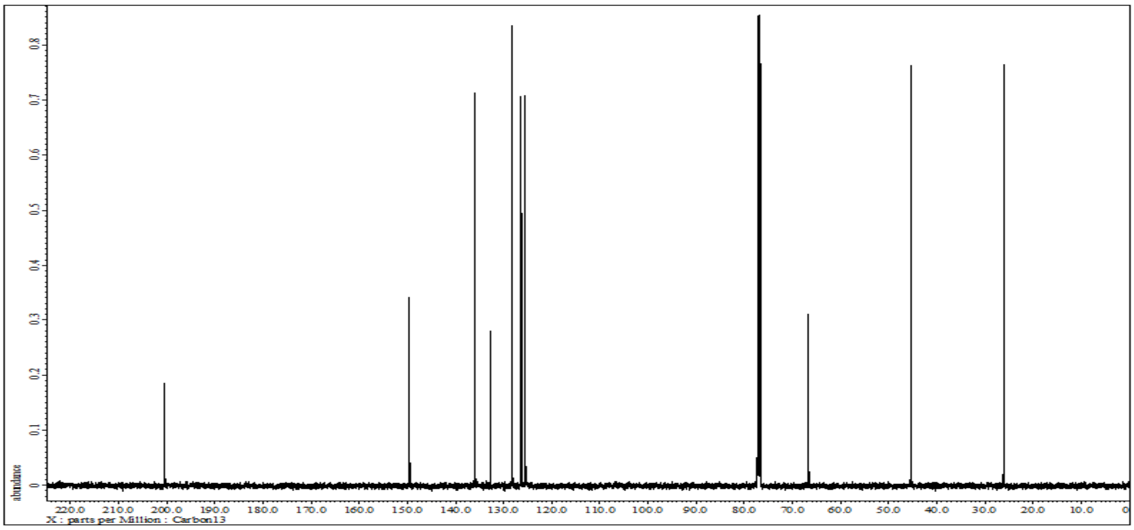

Supplementary Figure 82. ^{13}C NMR spectrum for α-chloroketone **2g**.

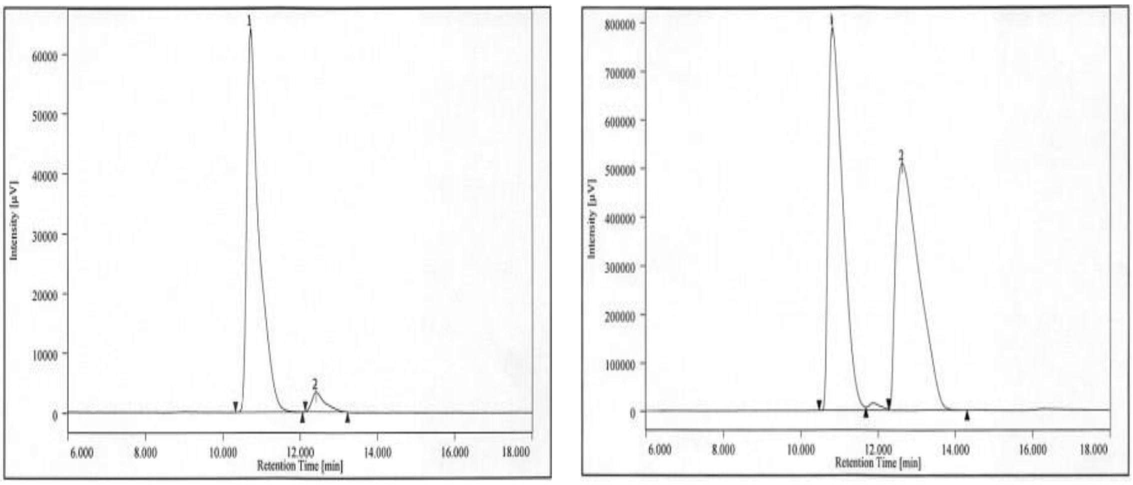

Peak	Retention Time [min]	Area [%]
1	10.7	94.823
2	12.4	5.177

Peak	Retention Time [min]	Area [%]
1	10.8	48.964
2	12.6	51.036

Supplementary Figure 83. HPLC spectra for α-chloroketone **2g**.

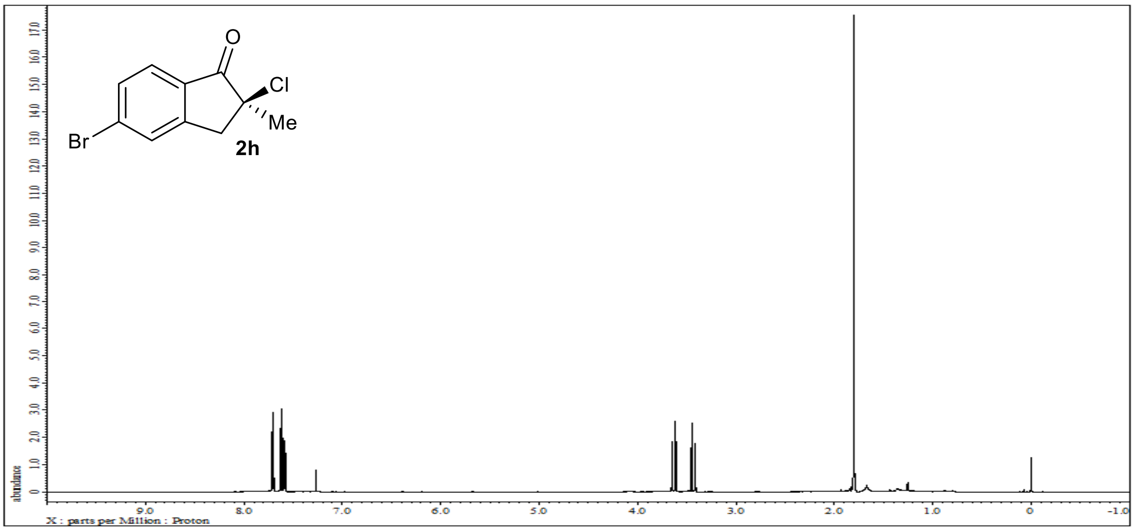

Supplementary Figure 84. ^{1}H NMR spectrum for α-chloroketone **2h**.

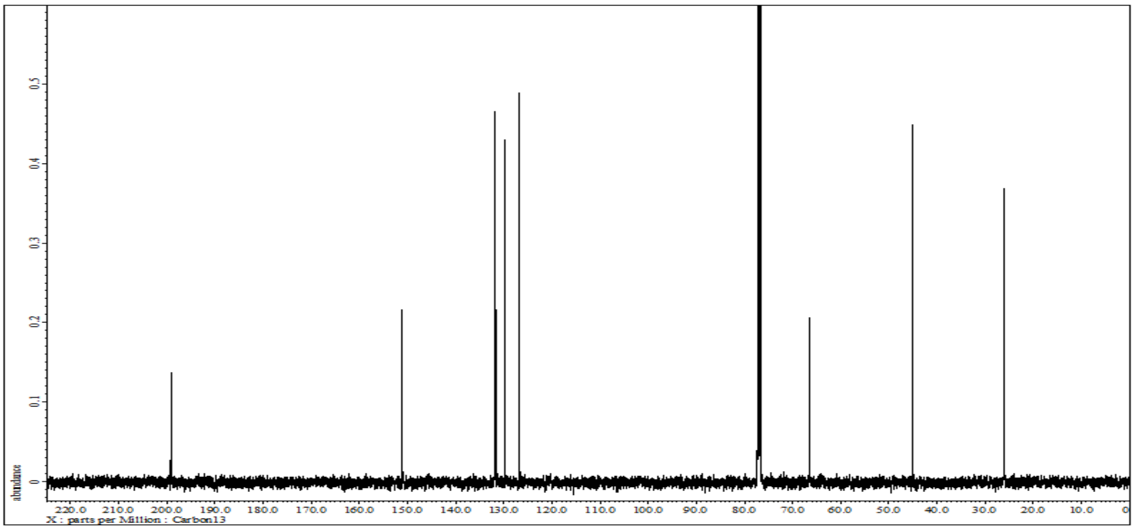

Supplementary Figure 85. ^{13}C NMR spectrum for α-chloroketone **2h**.

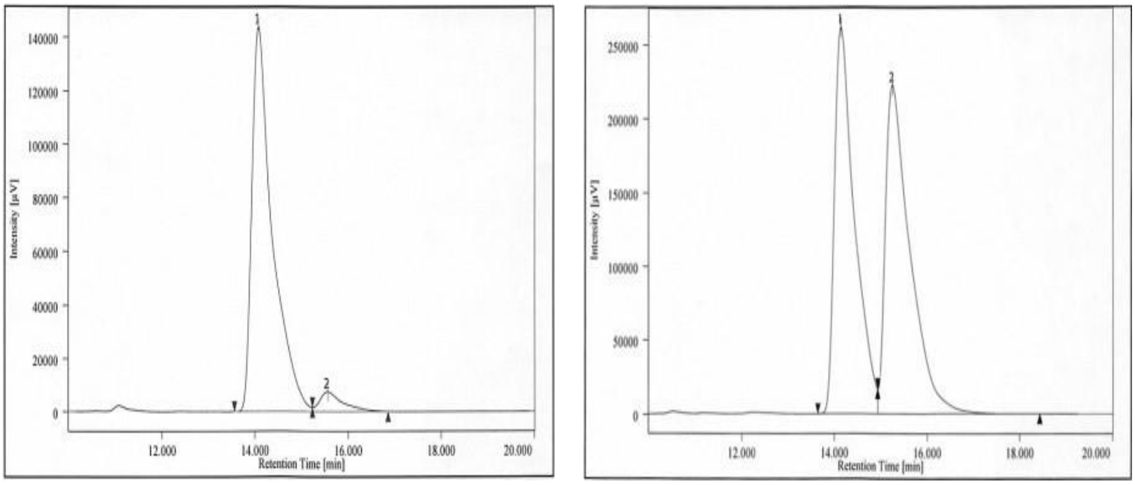

Peak	Retention Time [min]	Area [%]
1	14.1	94.527
2	15.6	5.473

Peak	Retention Time [min]	Area [%]
1	14.1	48.816
2	15.3	51.184

Supplementary Figure 86. HPLC spectra for α-chloroketone **2h**.

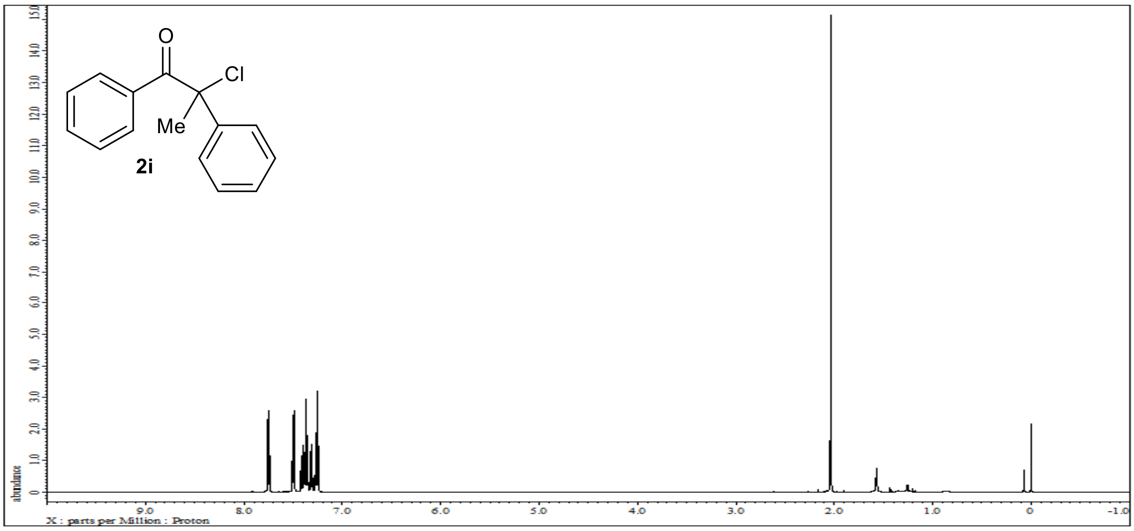

Supplementary Figure 87. ^{1}H NMR spectrum for α-chloroketone **2i**.

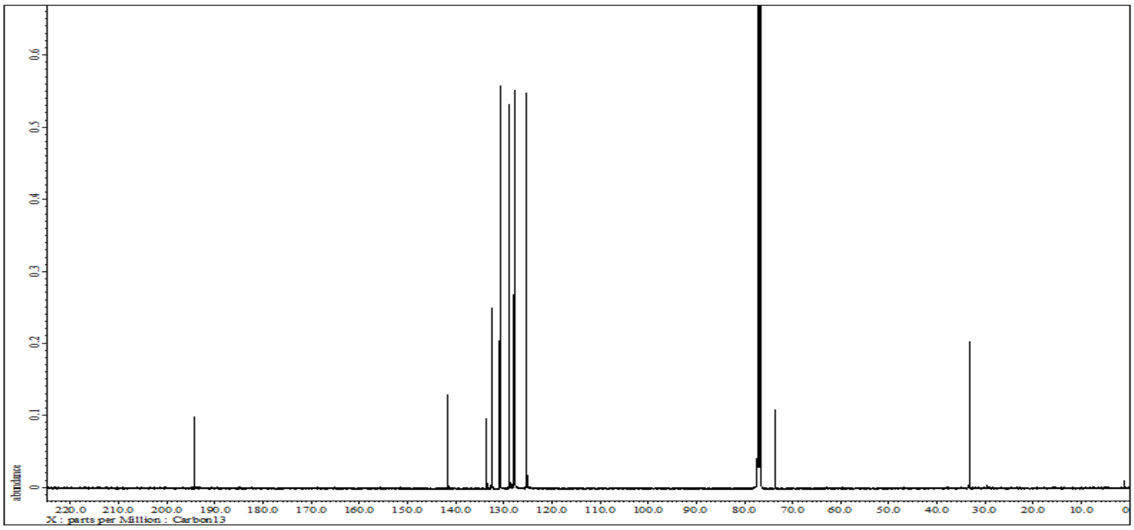

Supplementary Figure 88. ^{13}C NMR spectrum for α-chloroketone **2i**.

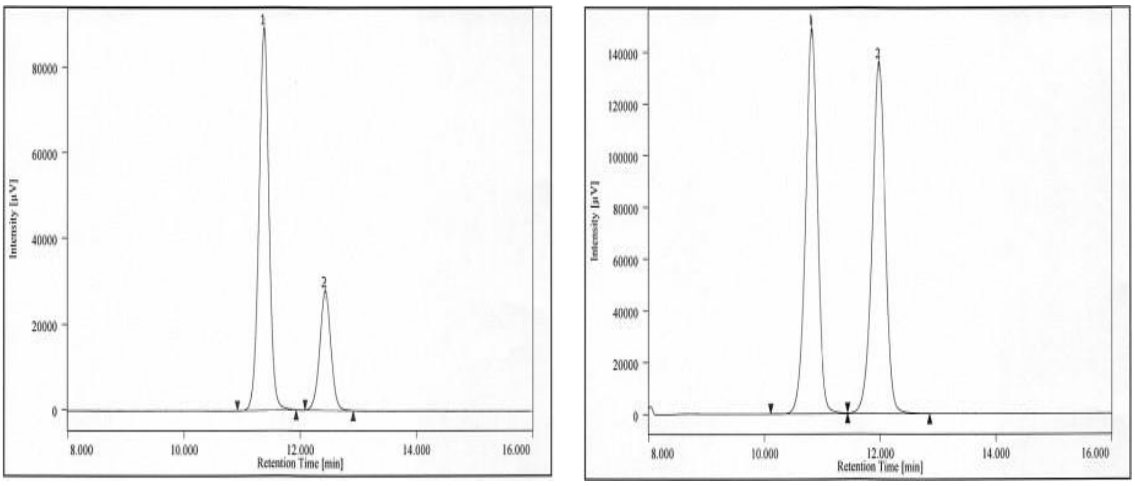

Peak	Retention Time [min]	Area [%]
1	11.4	74.189
2	12.4	25.811

Peak	Retention Time [min]	Area [%]
1	10.8	50.015
2	12.0	49.985

Supplementary Figure 89. HPLC spectra for α-chloroketone **2i**.

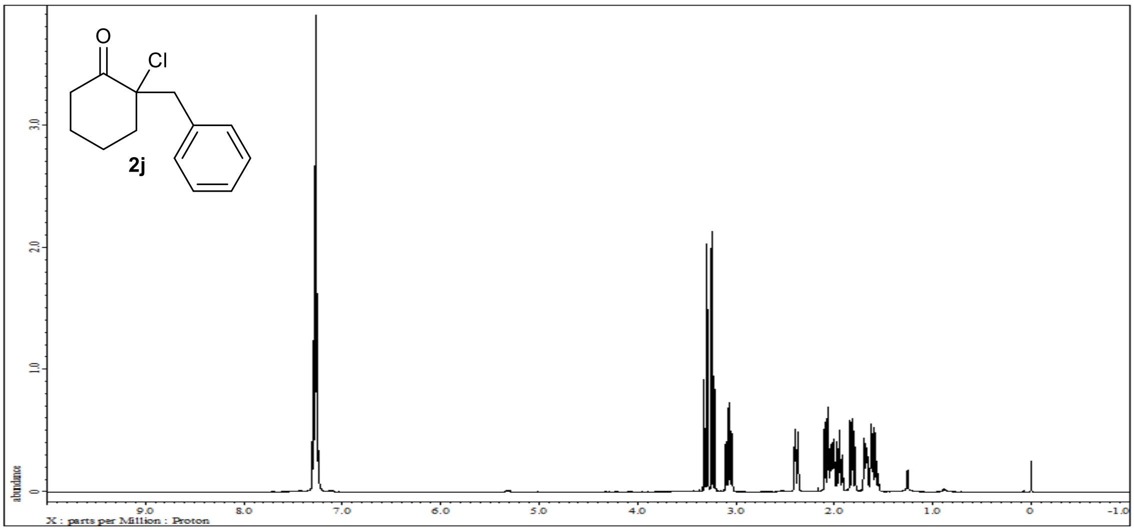

Supplementary Figure 90. ^{1}H NMR spectrum for α-chloroketone **2j**.

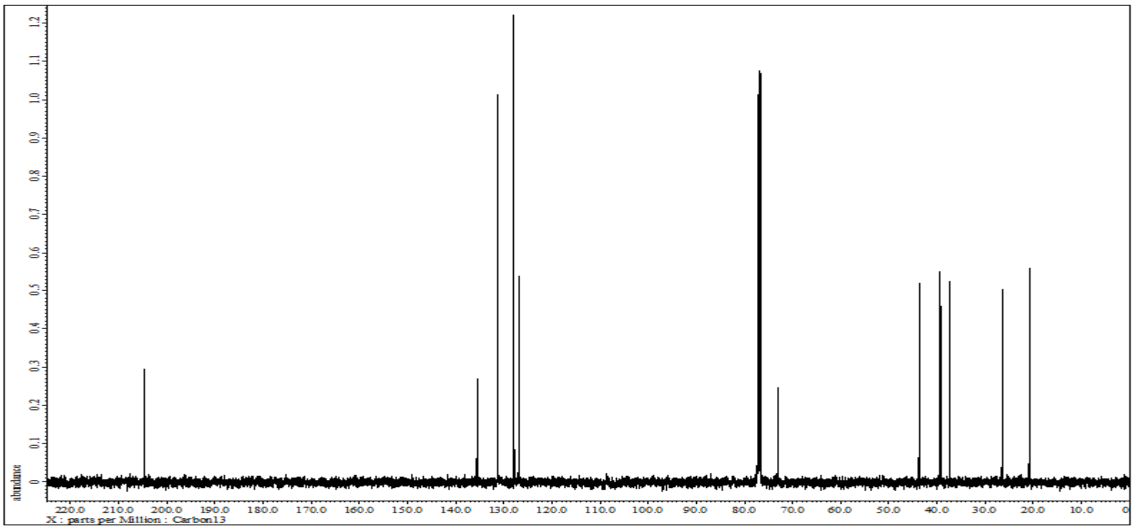

Supplementary Figure 91. ^{13}C NMR spectrum for α-chloroketone **2j**.

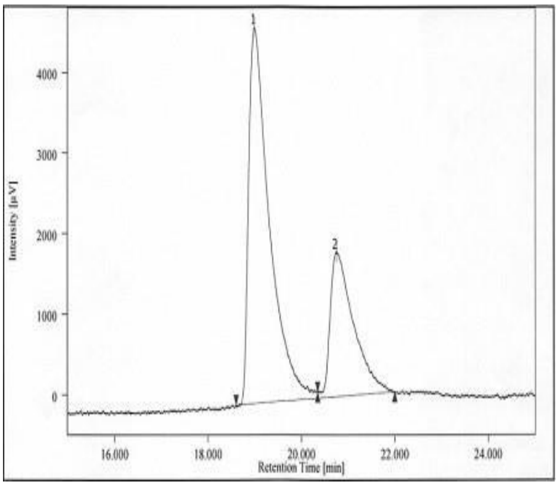

Peak	Retention Time [min]	Area [%]
1	19.0	69.793
2	20.7	30.207

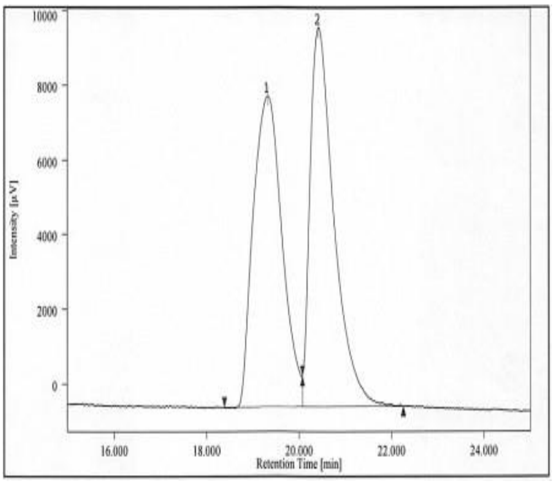

Peak	Retention Time [min]	Area [%]
1	19.3	49.575
2	20.4	50.425

Supplementary Figure 92. HPLC spectra for α-chloroketone **2j**.

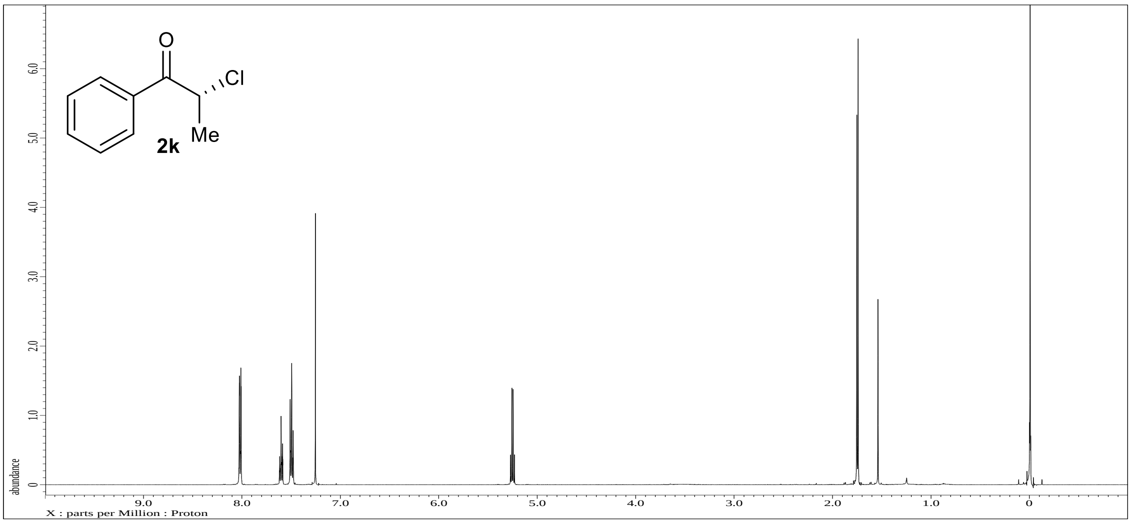

Supplementary Figure 93. ^{1}H NMR spectrum for α-chloroketone **2k**.

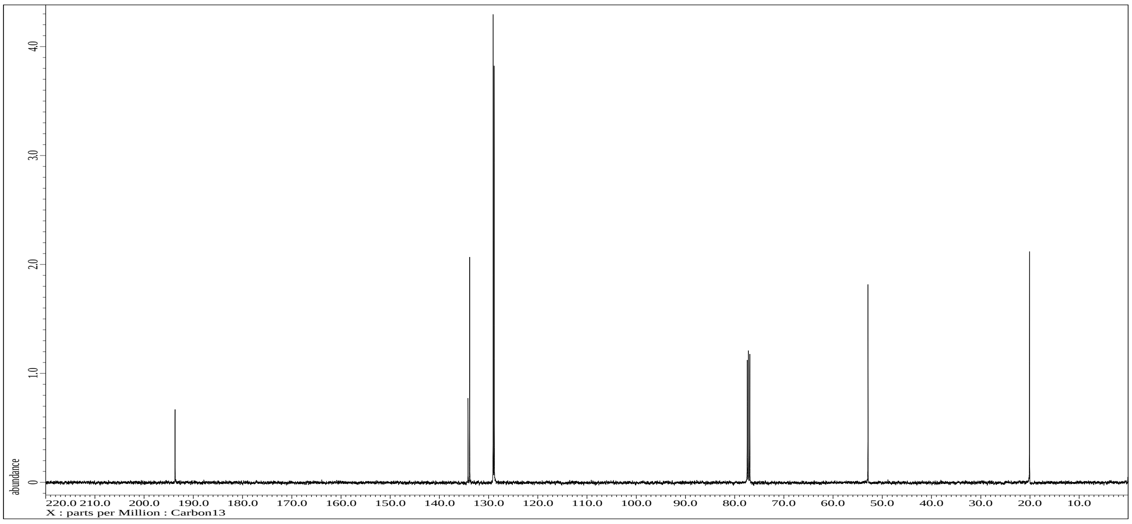

Supplementary Figure 94. ^{13}C NMR spectrum for α-chloroketone **2k**.

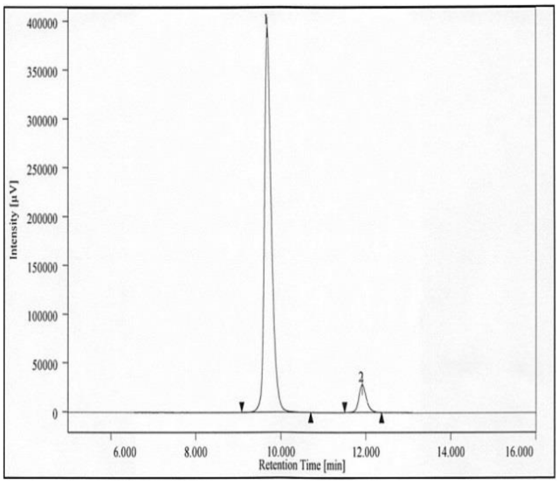

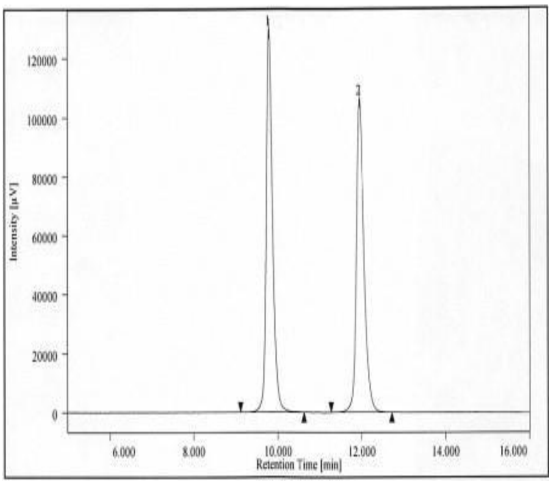

Peak	Retention Time [min]	Area [%]
1	9.7	92.604
2	11.9	7.396

Peak	Retention Time [min]	Area [%]
1	9.8	50.157
2	12.0	49.843

Supplementary Figure 95. HPLC spectra for α-chloroketone **2k**.

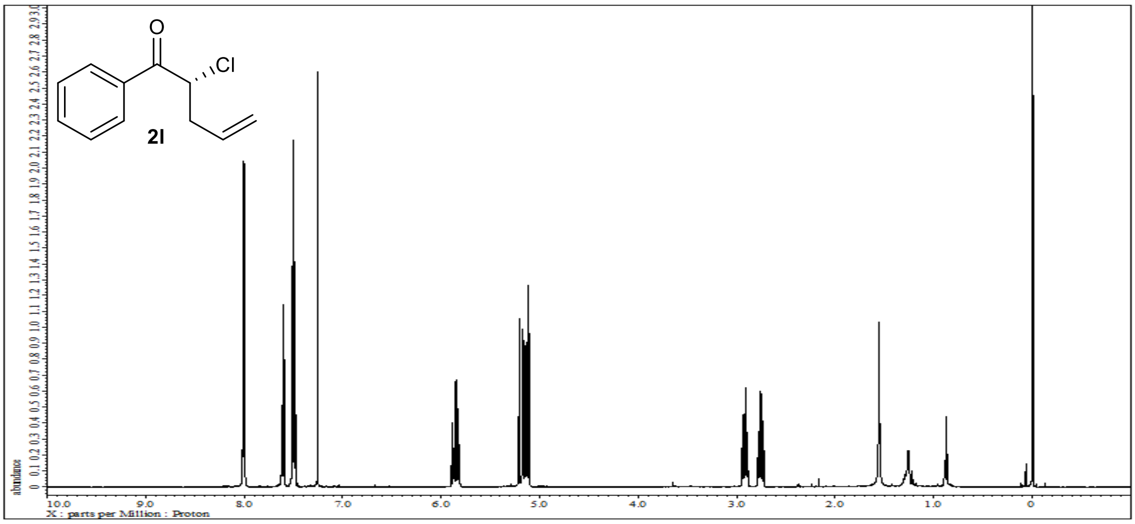

Supplementary Figure 96. ^{1}H NMR spectrum for α-chloroketone **2l**.

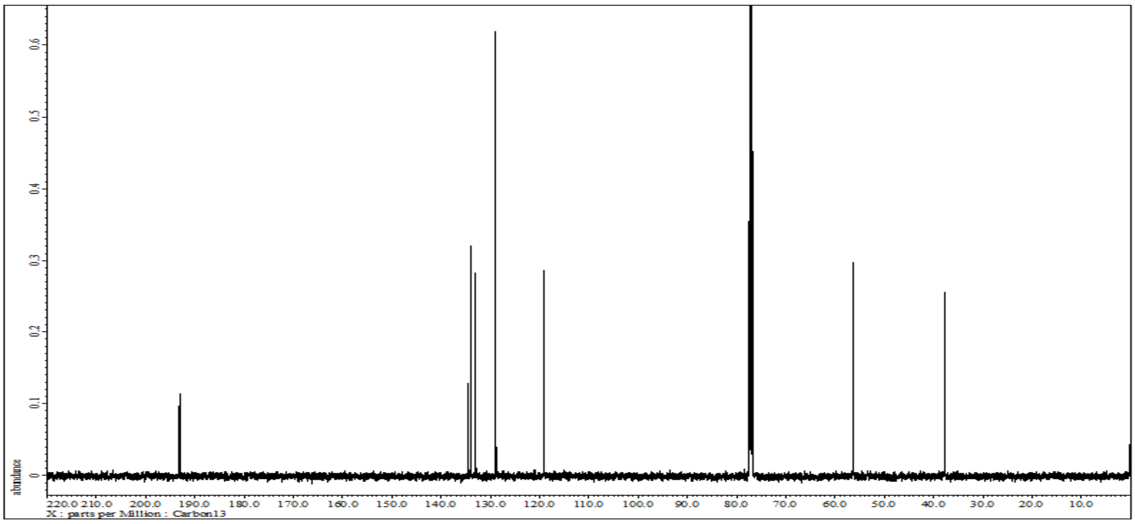

Supplementary Figure 97. ^{13}C NMR spectrum for α-chloroketone **2l**.

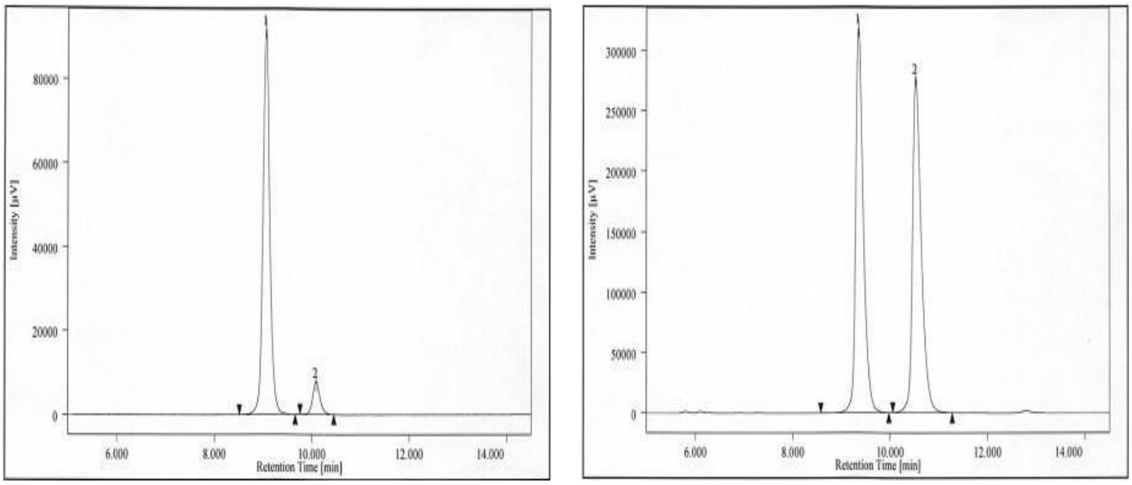

Peak	Retention Time [min]	Area [%]
1	9.1	91.586
2	10.1	8.414

Peak	Retention Time [min]	Area [%]
1	9.4	50.010
2	10.5	49.990

Supplementary Figure 98. HPLC spectra for α-chloroketone **2l**.

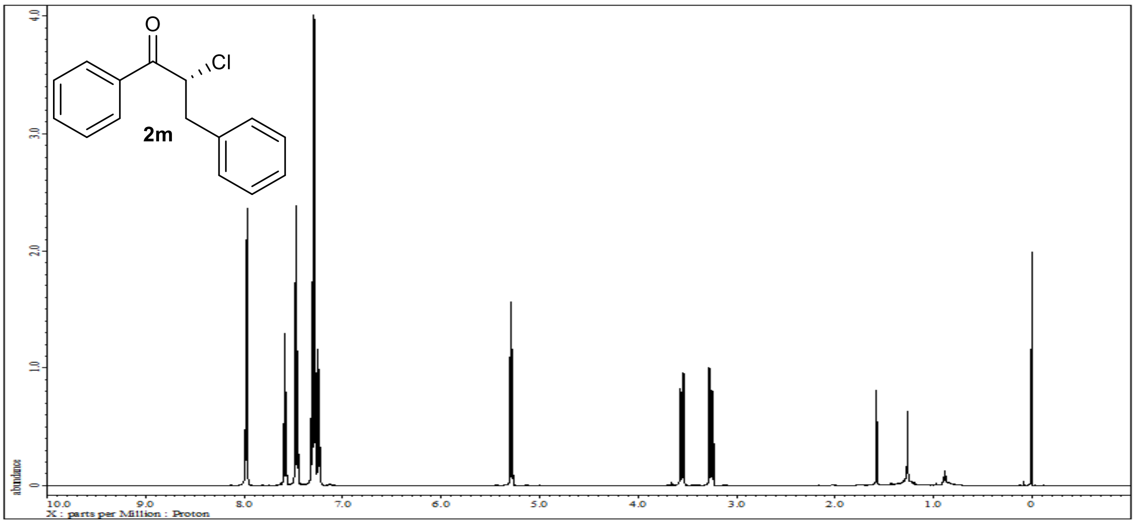

Supplementary Figure 99. ^{1}H NMR spectrum for α-chloroketone **2m**.

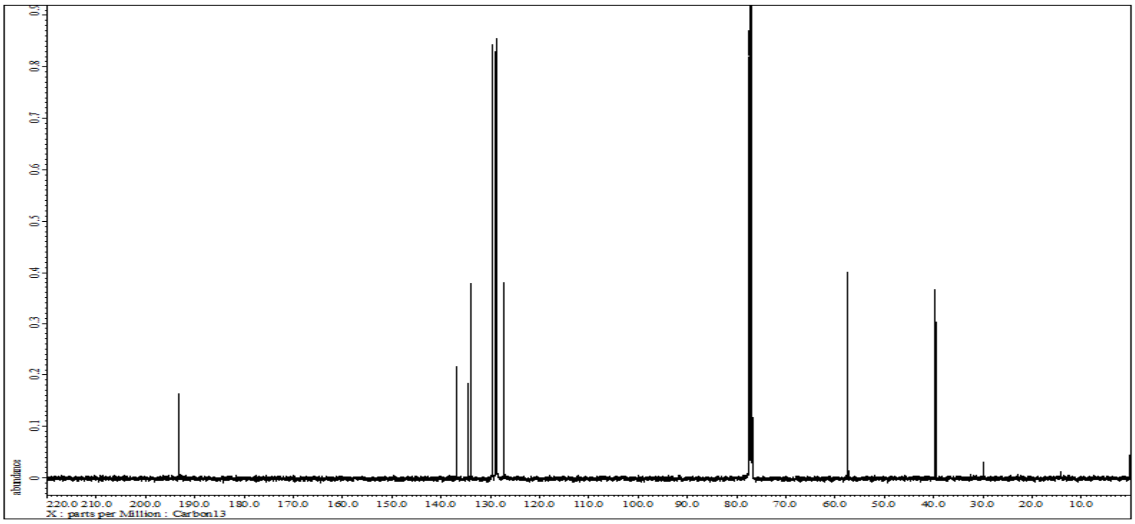

Supplementary Figure 100. ^{13}C NMR spectrum for α-chloroketone **2m**.

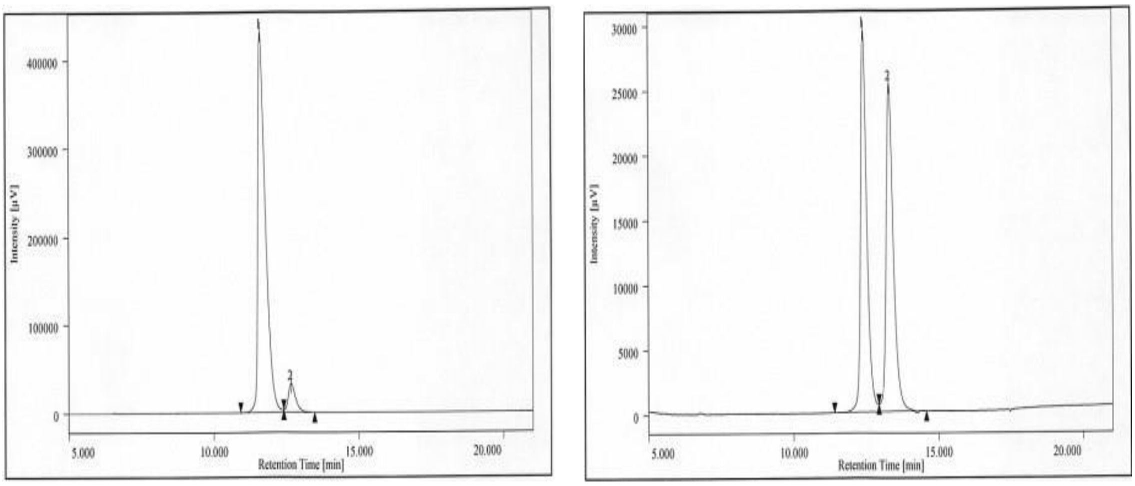

Peak	Retention Time [min]	Area [%]
1	11.6	93.058
2	12.7	6.942

Peak	Retention Time [min]	Area [%]
1	12.4	50.069
2	13.3	49.931

Supplementary Figure 101. HPLC spectra for α-chloroketone **2m**.

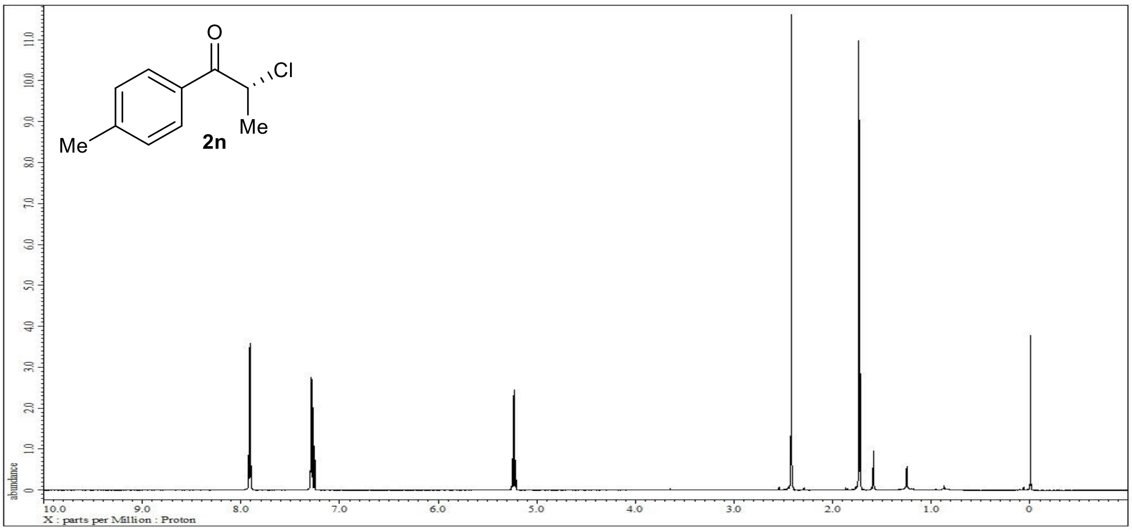

Supplementary Figure 102. ^{1}H NMR spectrum for α-chloroketone **2n**.

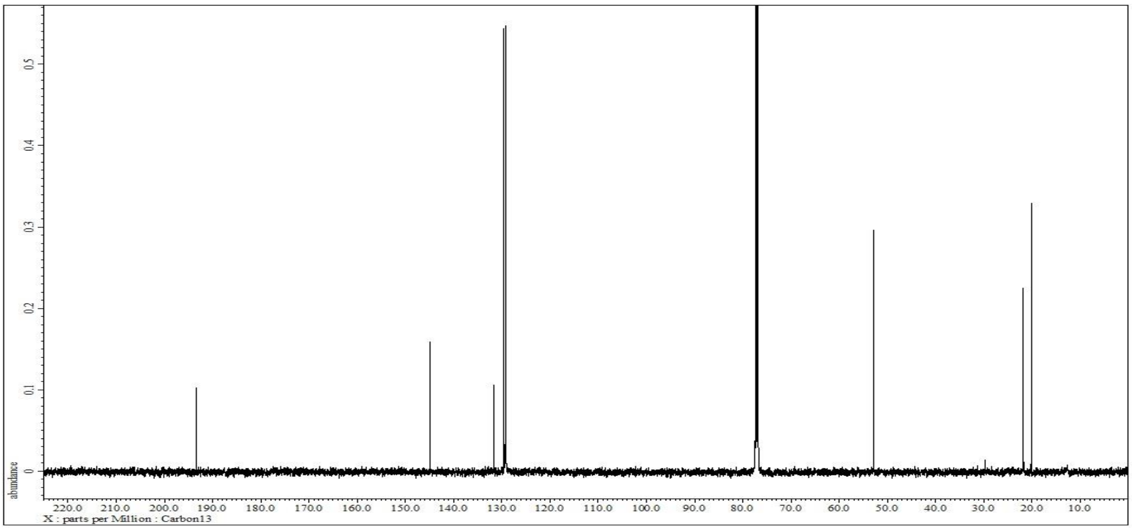

Supplementary Figure 103. ^{13}C NMR spectrum for α-chloroketone **2n**.

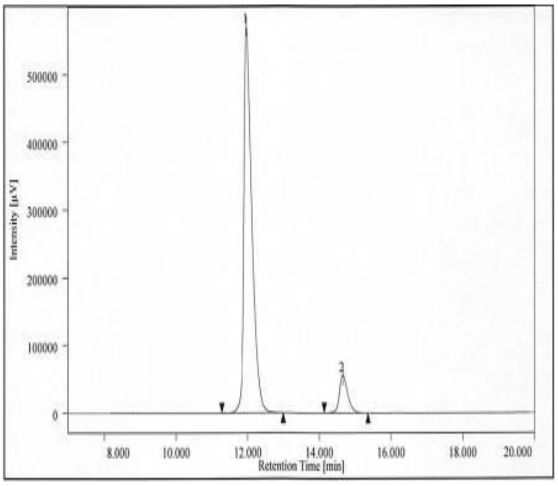

Peak	Retention Time [min]	Area [%]
1	12.0	90.854
2	14.7	9.146

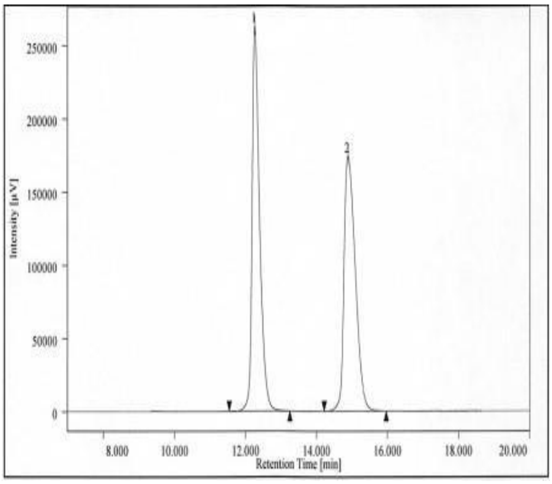

Peak	Retention Time [min]	Area [%]
1	12.3	49.805
2	14.9	50.195

Supplementary Figure 104. HPLC spectra for α-chloroketone **2n**.

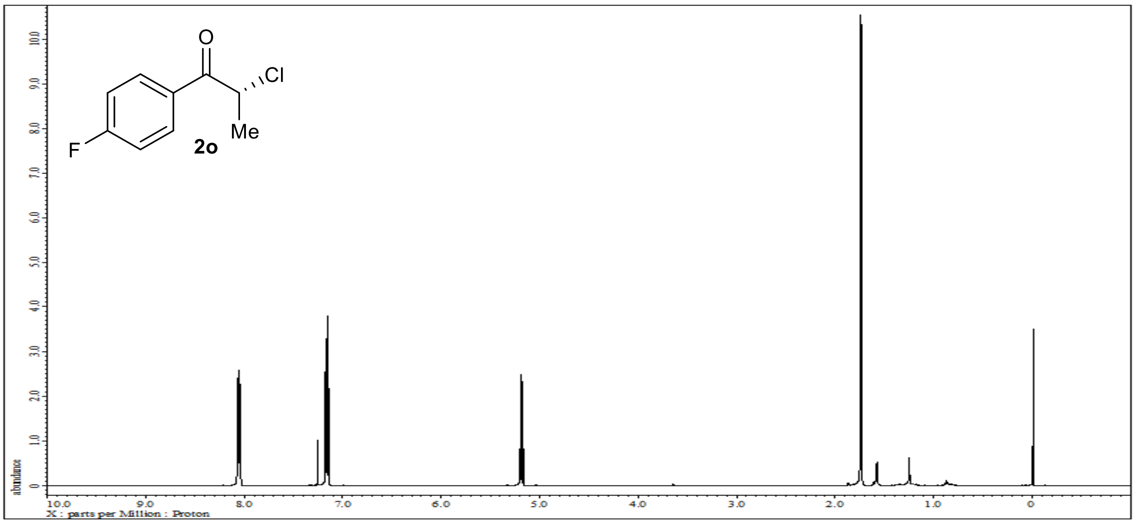

Supplementary Figure 105. ^{1}H NMR spectrum for α-chloroketone **2o**.

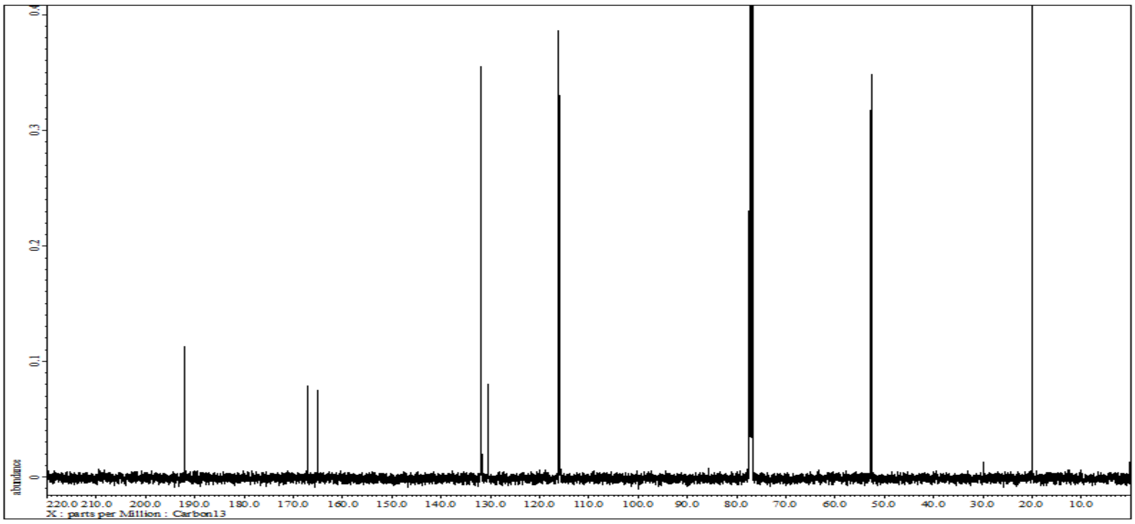

Supplementary Figure 106. ^{13}C NMR spectrum for α-chloroketone **2o**.

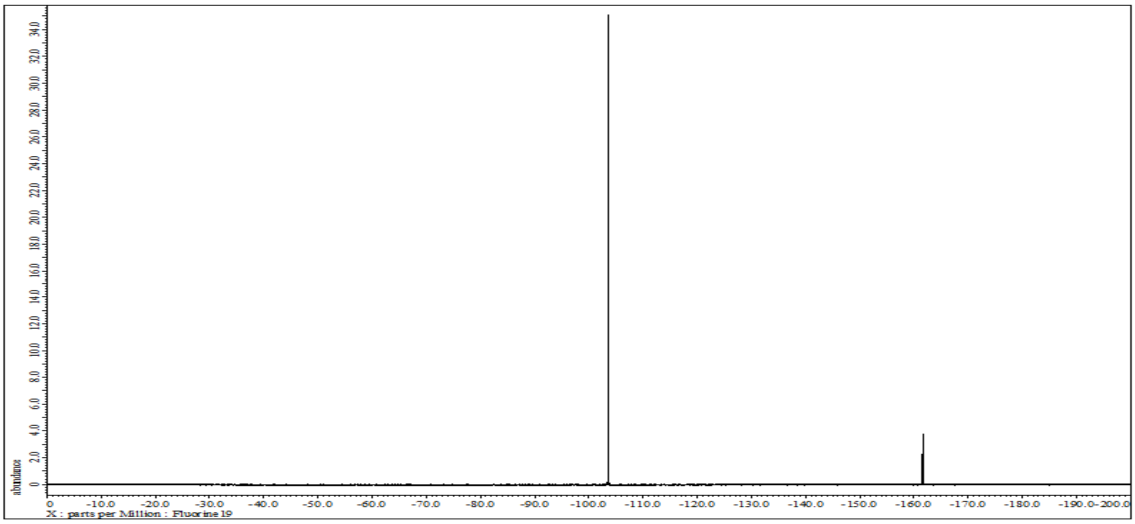

Supplementary Figure 107. ^{19}F NMR spectrum for α-chloroketone **2o**.

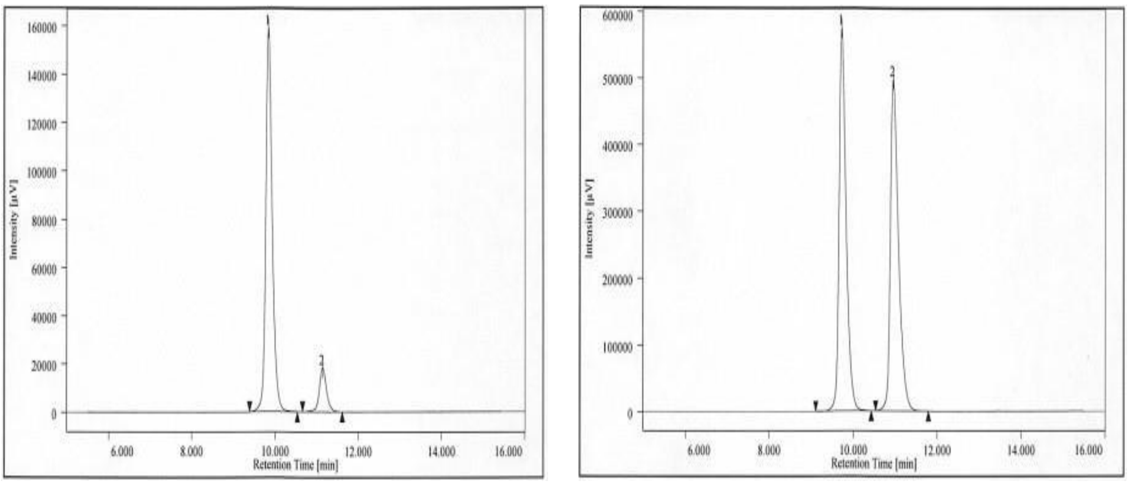

Peak	Retention Time [min]	Area [%]
1	9.8	88.815
2	11.1	11.185

Peak	Retention Time [min]	Area [%]
1	9.7	49.959
2	11.0	50.041

Supplementary Figure 108. HPLC spectra for α-chloroketone **2o**.

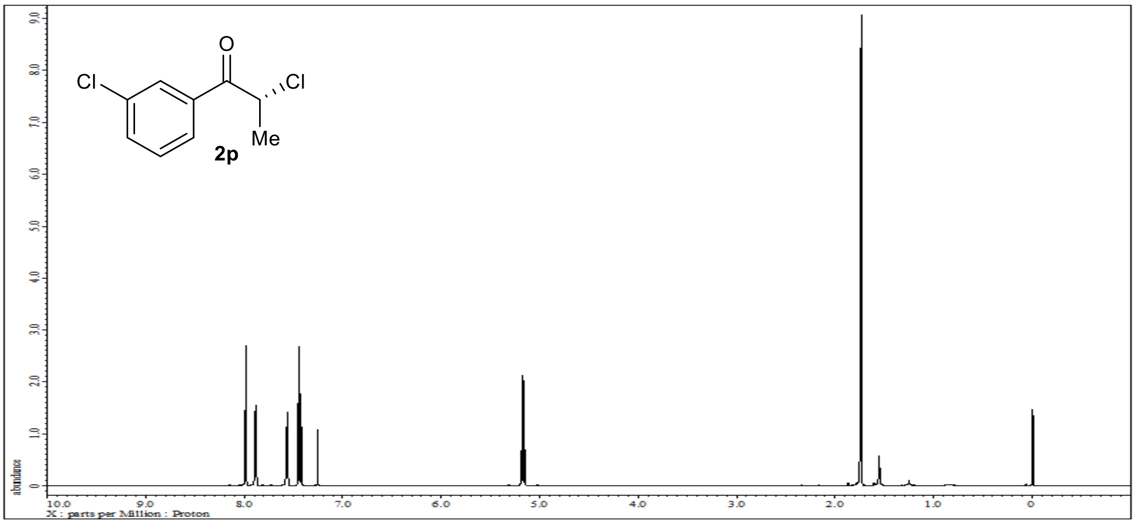

Supplementary Figure 109. ^{1}H NMR spectrum for α-chloroketone **2p**.

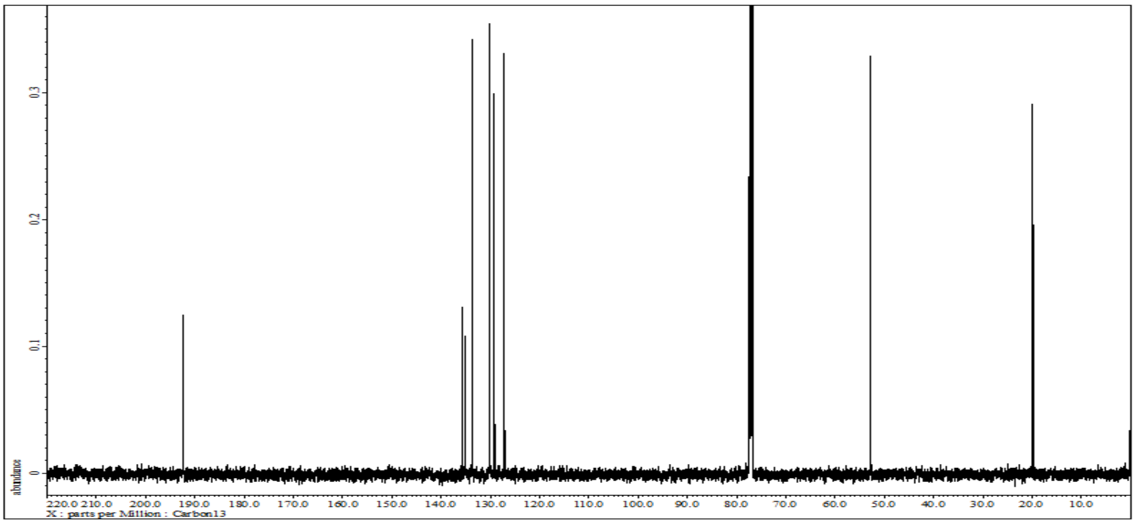

Supplementary Figure 110. ^{13}C NMR spectrum for α-chloroketone **2p**.

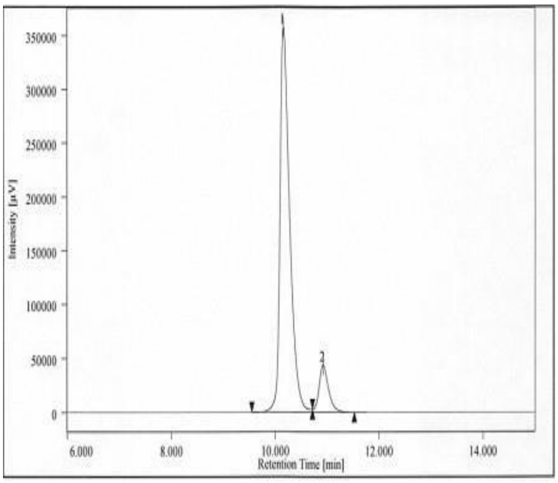

Peak	Retention Time [min]	Area [%]
1	10.2	89.430
2	10.9	10.570

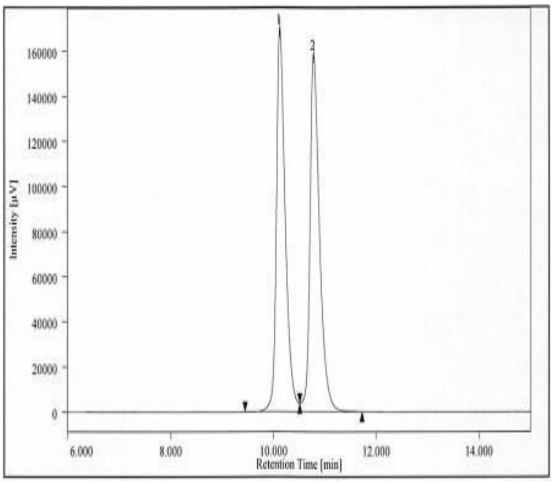

Peak	Retention Time [min]	Area [%]
1	10.1	49.780
2	10.8	50.220

Supplementary Figure 111. HPLC spectra for α-chloroketone **2p**.

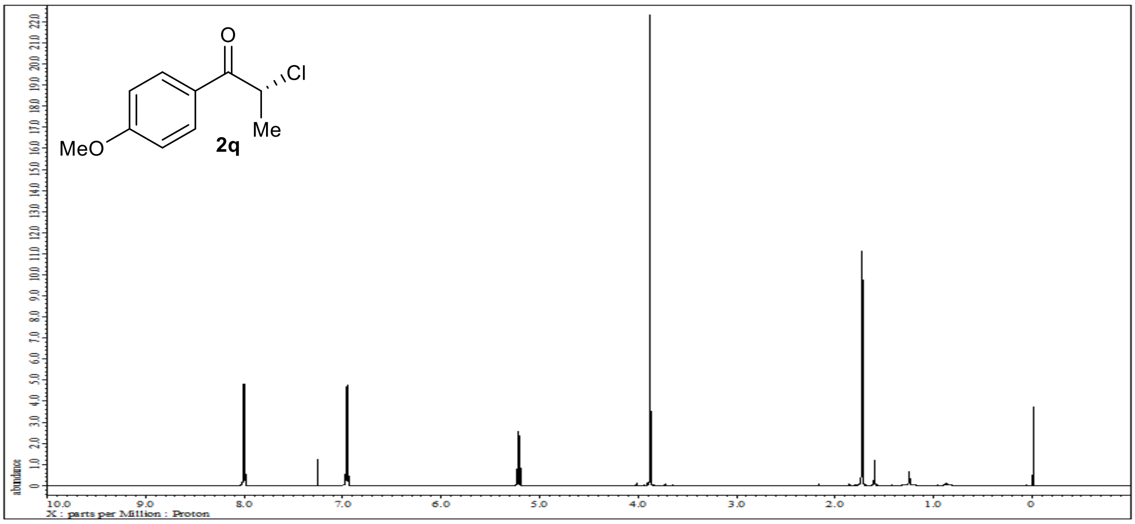

Supplementary Figure 112. ^{1}H NMR spectrum for α-chloroketone **2q**.

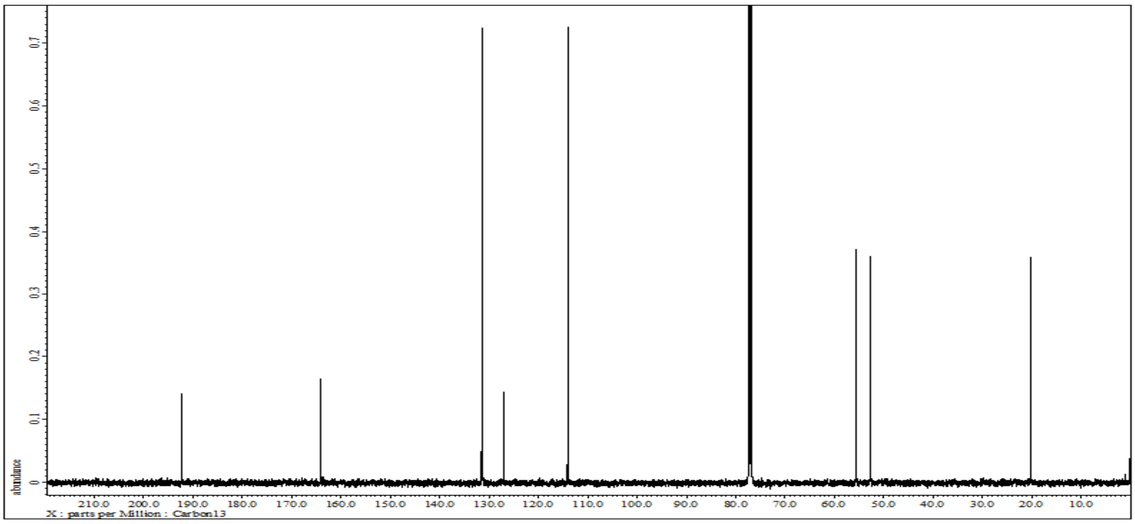

Supplementary Figure 113. ^{13}C NMR spectrum for α-chloroketone **2q**.

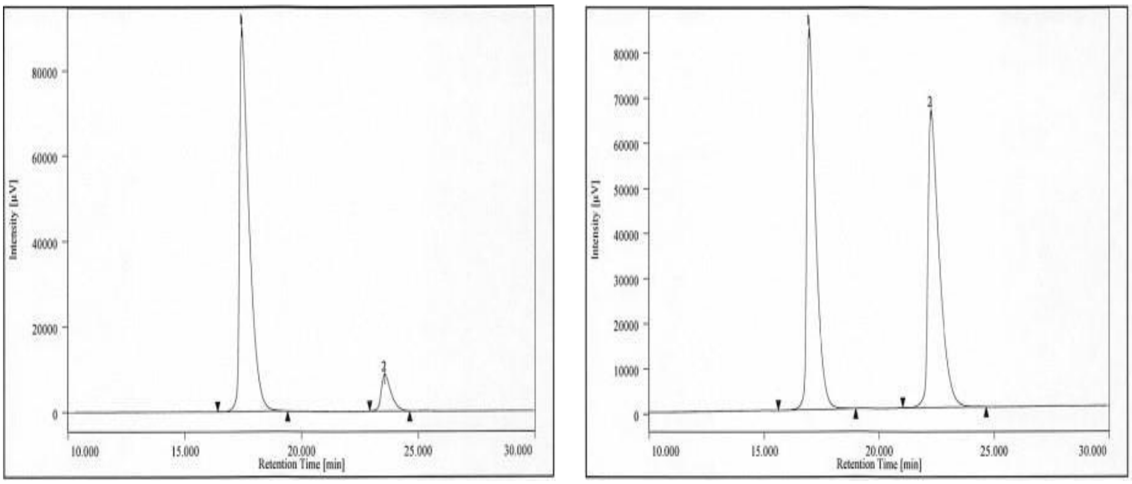

Peak	Retention Time [min]	Area [%]
1	17.5	91.339
2	23.6	8.661

Peak	Retention Time [min]	Area [%]
1	17.0	49.936
2	22.3	50.064

Supplementary Figure 114. HPLC spectra for α-chloroketone **2q**.

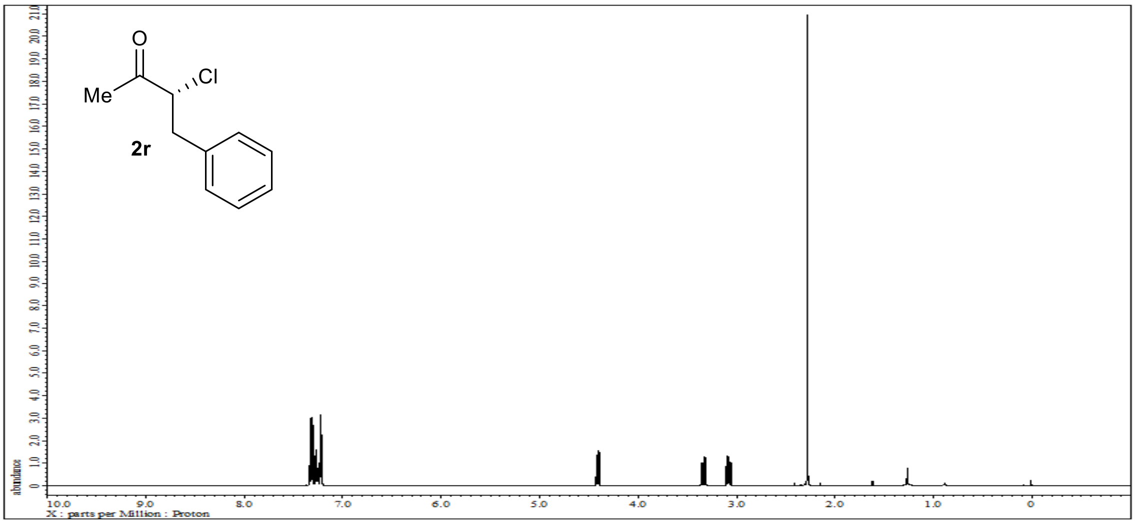

Supplementary Figure 115. ^{1}H NMR spectrum for α-chloroketone **2r**.

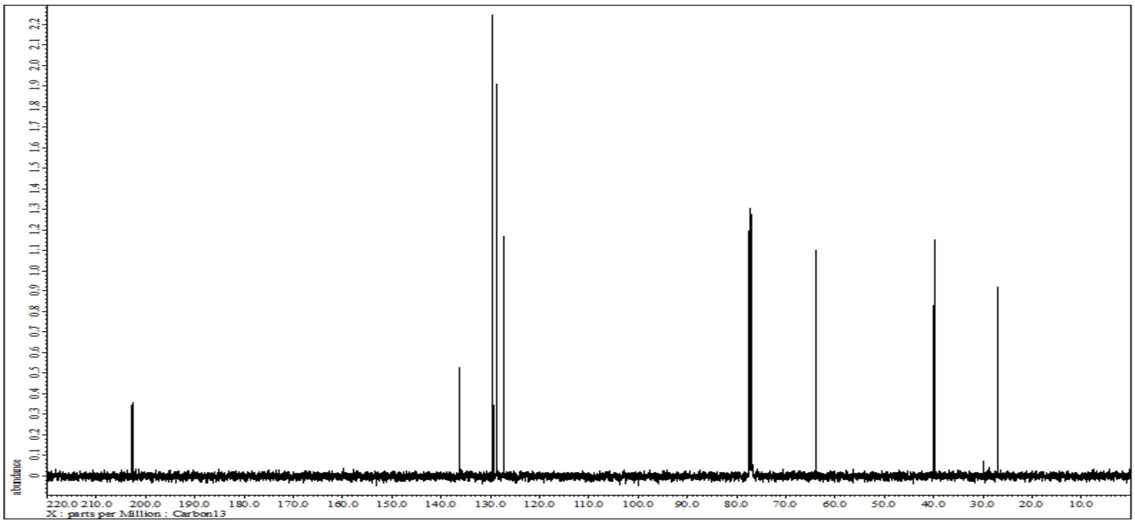

Supplementary Figure 116. ^{13}C NMR spectrum for α-chloroketone **2r**.

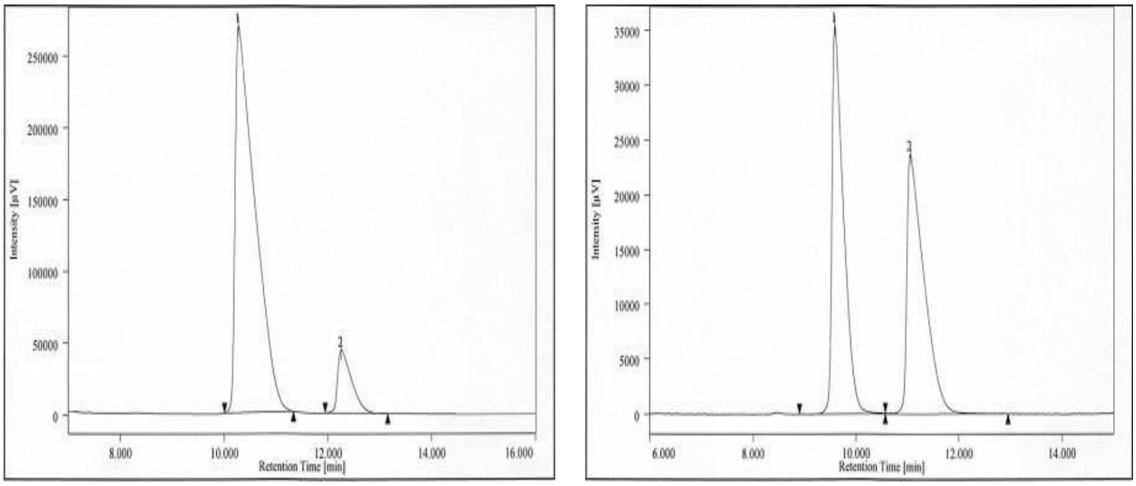

Peak	Retention Time [min]	Area [%]
1	10.3	89.723
2	12.3	10.277

Peak	Retention Time [min]	Area [%]
1	9.6	50.354
2	11.1	49.646

Supplementary Figure 117. HPLC spectra for α-chloroketone **2r**.

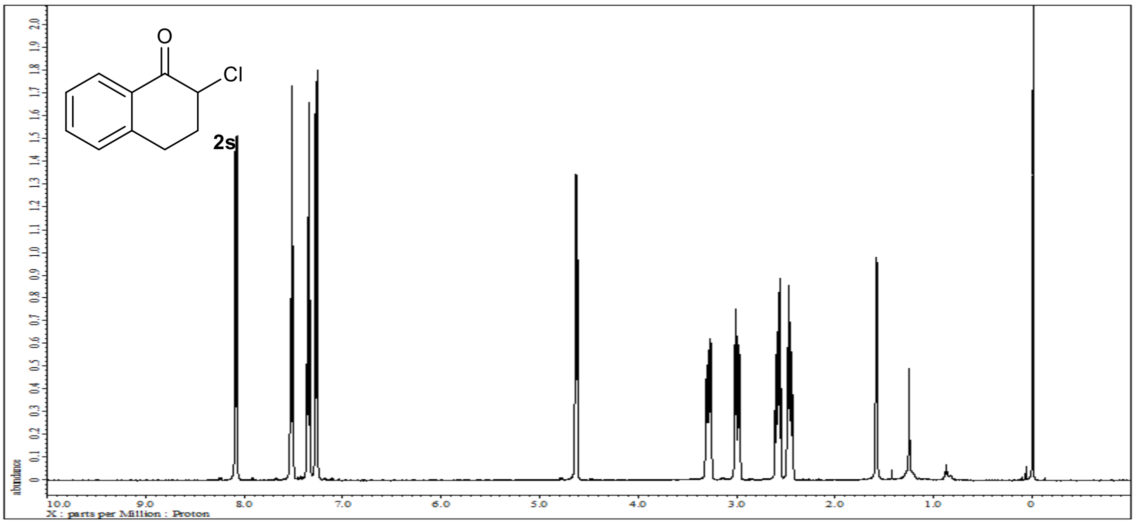

Supplementary Figure 118. ^{1}H NMR spectrum for α-chloroketone **2s**.

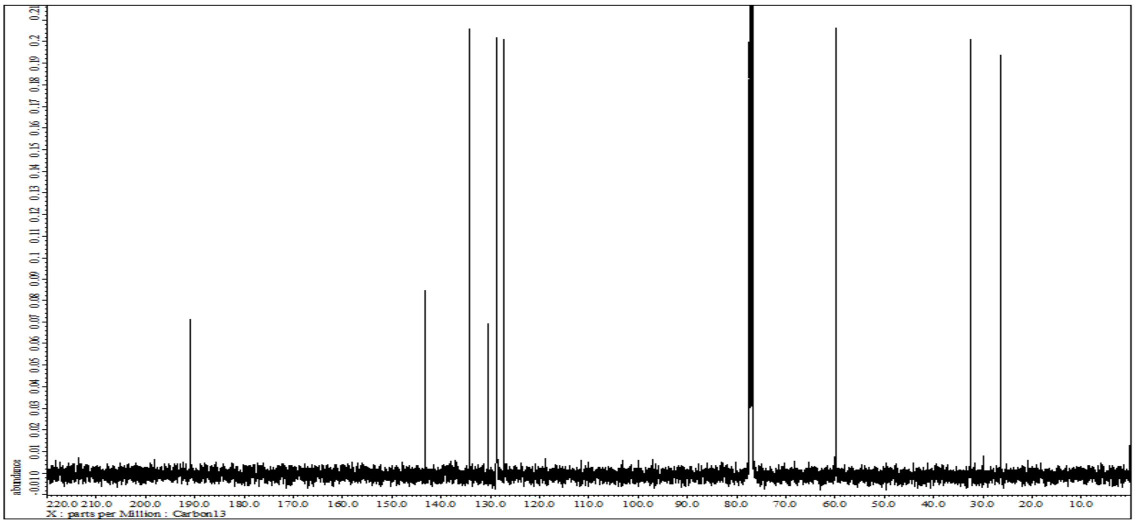

Supplementary Figure 119. ^{13}C NMR spectrum for α-chloroketone **2s**.

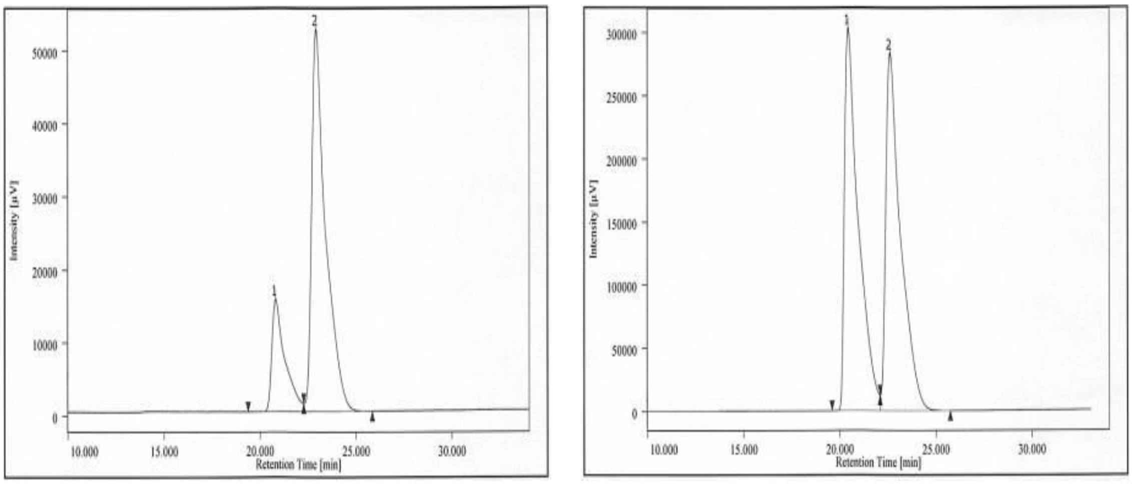

Peak	Retention Time [min]	Area [%]
1	20.8	21.666
2	22.9	78.334

Peak	Retention Time [min]	Area [%]
1	20.4	50.059
2	22.6	49.941

Supplementary Figure 120. HPLC spectra for α-chloroketone **2s**.

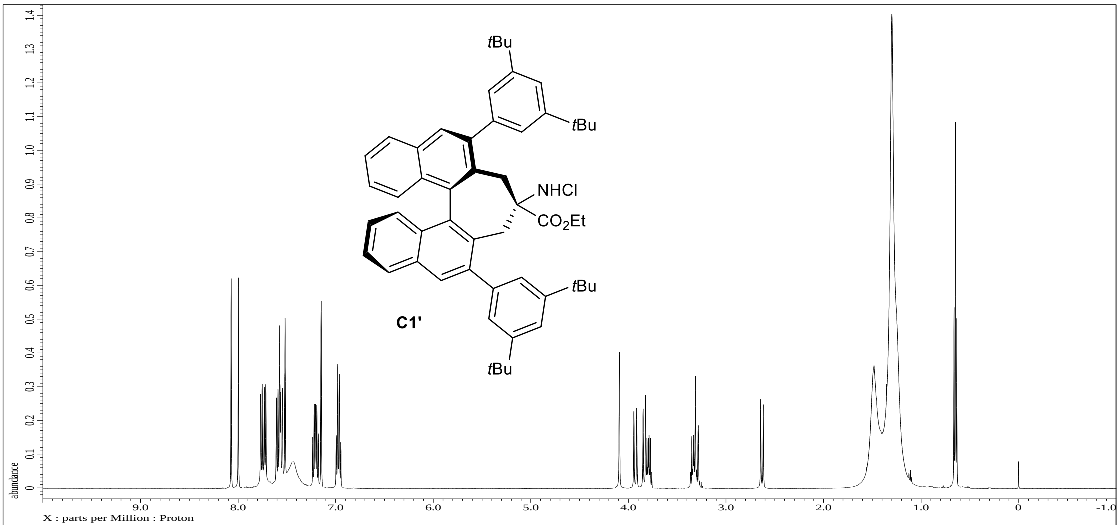

Supplementary Figure 121. ^{1}H NMR spectrum for **C1’**.

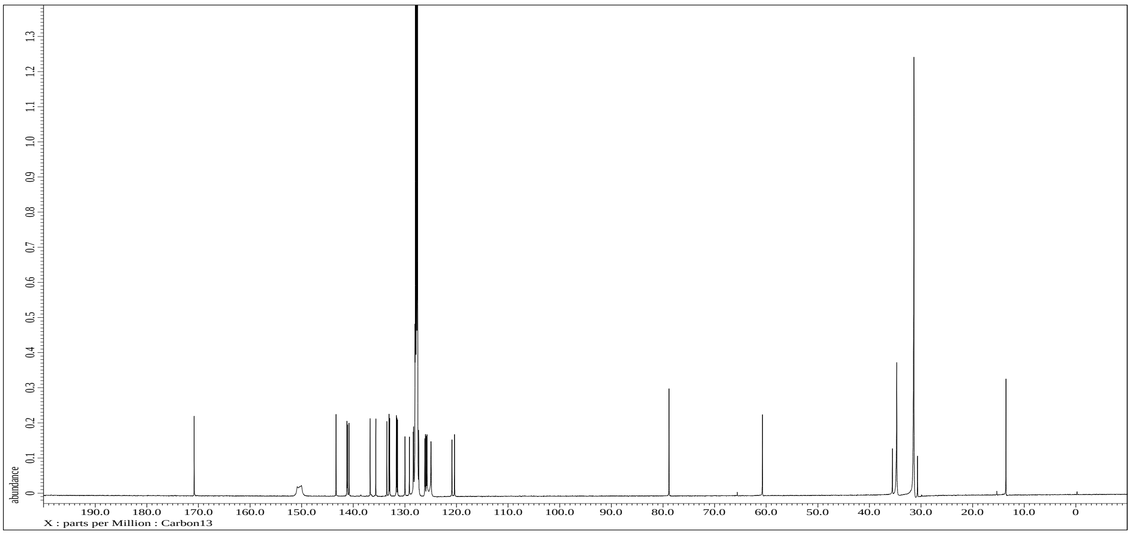

Supplementary Figure 122. ^{13}C NMR spectrum for **C1’**.

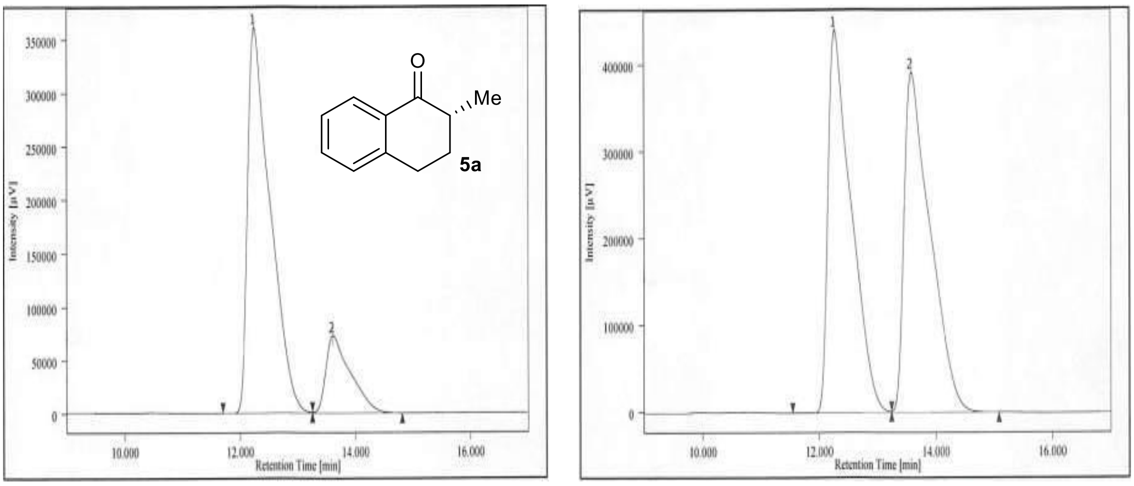

Peak	Retention Time [min]	Area [%]
1	12.3	82.130
2	13.6	17.870

Peak	Retention Time [min]	Area [%]
1	12.3	49.888
2	13.6	50.112

Supplementary Figure 123. HPLC spectra for α-methyltetralone **5a**.

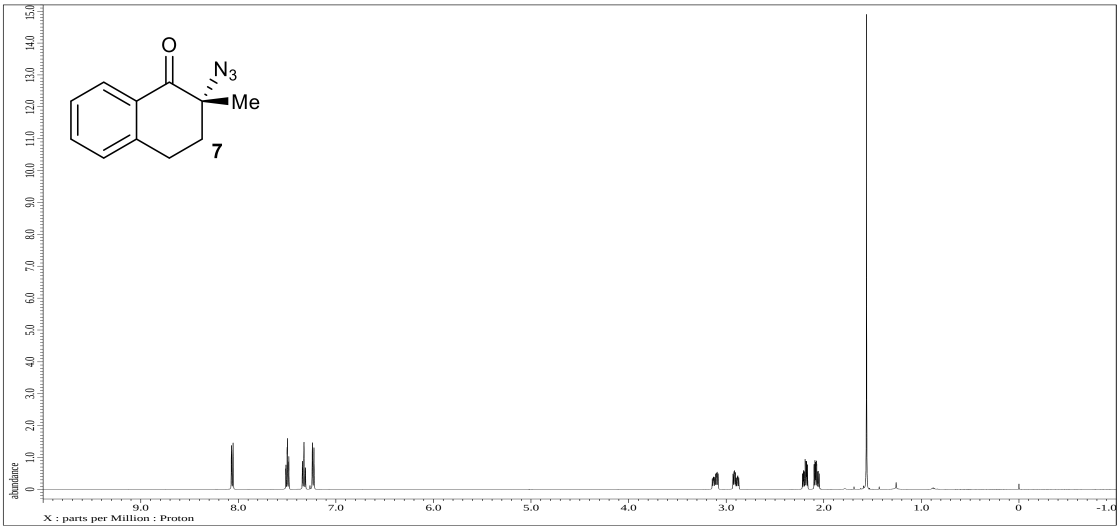

Supplementary Figure 124. ^{1}H NMR spectrum for α-azideketone **7**.

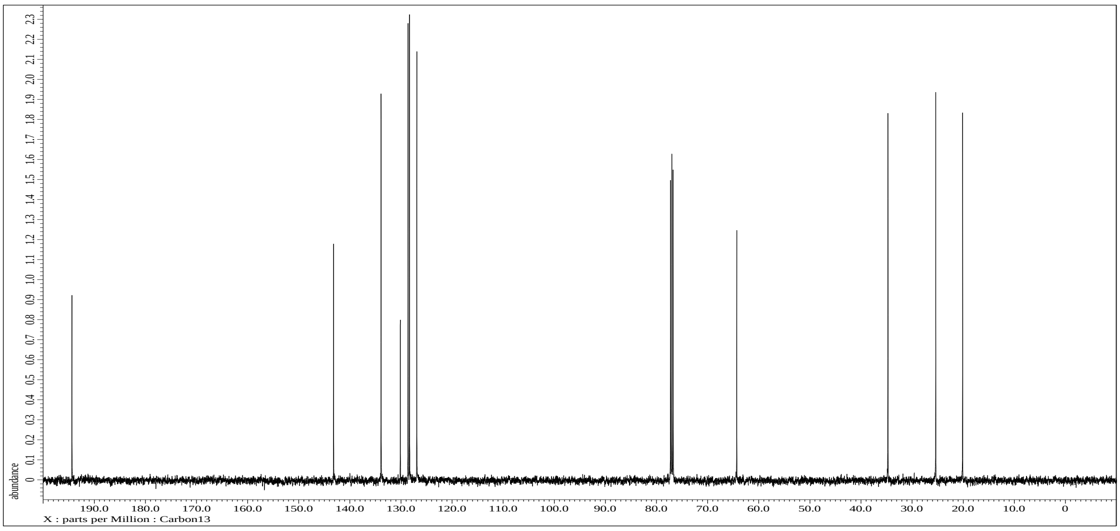

Supplementary Figure 125. ^{13}C NMR spectrum for α-azideketone **7**.

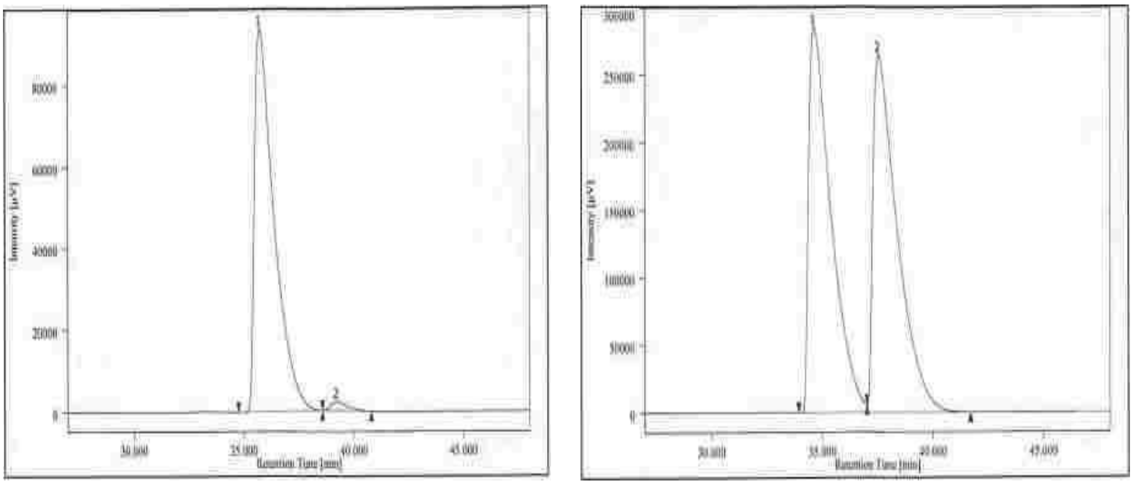

Peak	Retention Time [min]	Area [%]
1	35.7	98.028
2	39.2	1.972

Peak	Retention Time [min]	Area [%]
1	34.6	49.740
2	37.5	50.260

Supplementary Figure 126. HPLC spectra for α-azideketone **7**.

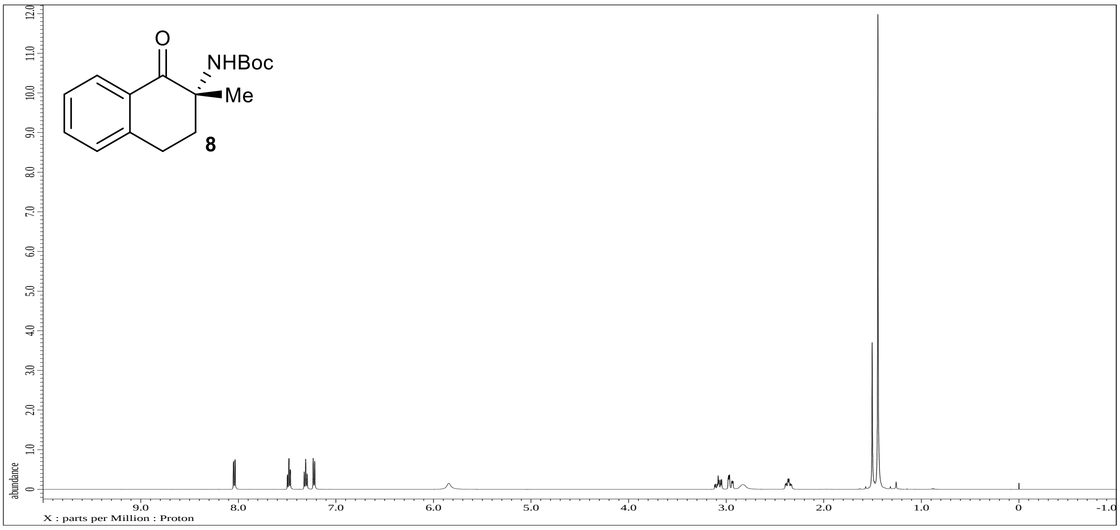

Supplementary Figure 127. ^{1}H NMR spectrum for *N*-Boc-protected α-aminoketone **8**.

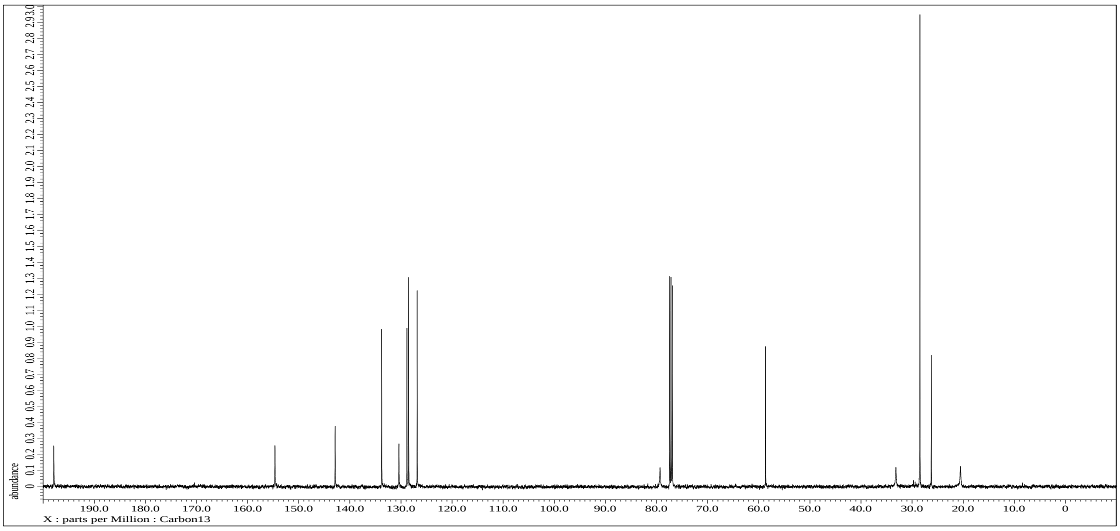

Supplementary Figure 128. ^{13}C NMR spectrum for *N*-Boc-protected α-aminoketone **8**.

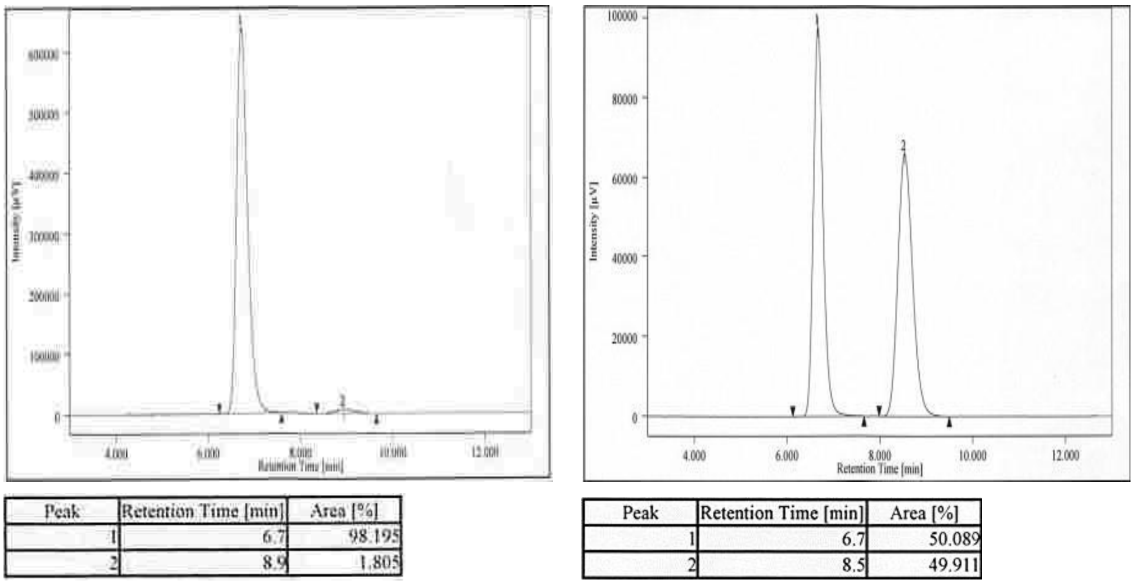

Peak	Retention Time [min]	Area [%]
1	6.7	98.195
2	8.9	1.805

Peak	Retention Time [min]	Area [%]
1	6.7	50.089
2	8.5	49.911

Supplementary Figure 129. HPLC spectra for *N*-Boc-protected α-aminoketone **8**.

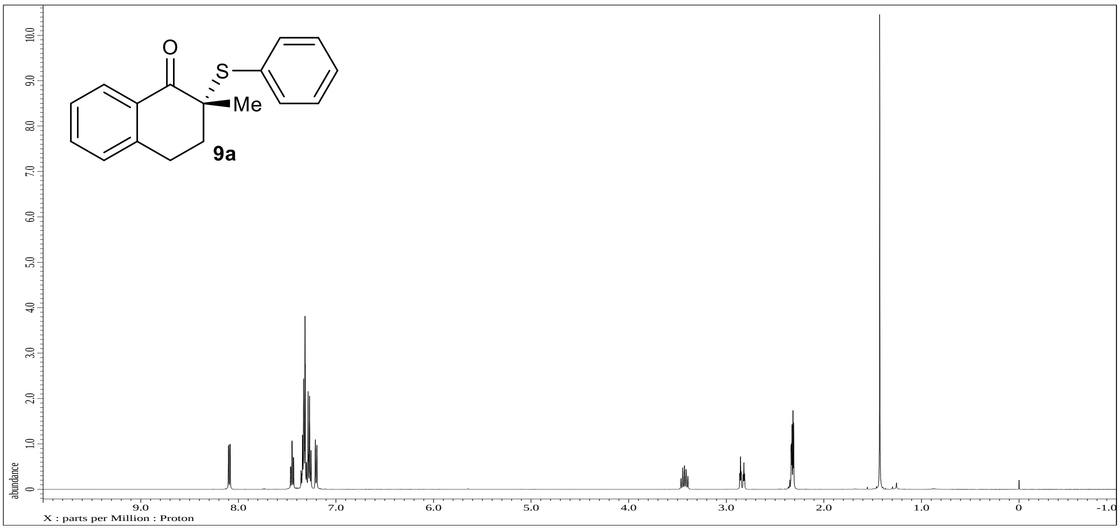

Supplementary Figure 130. ^{1}H NMR spectrum for α-sulfenylketone **9a**.

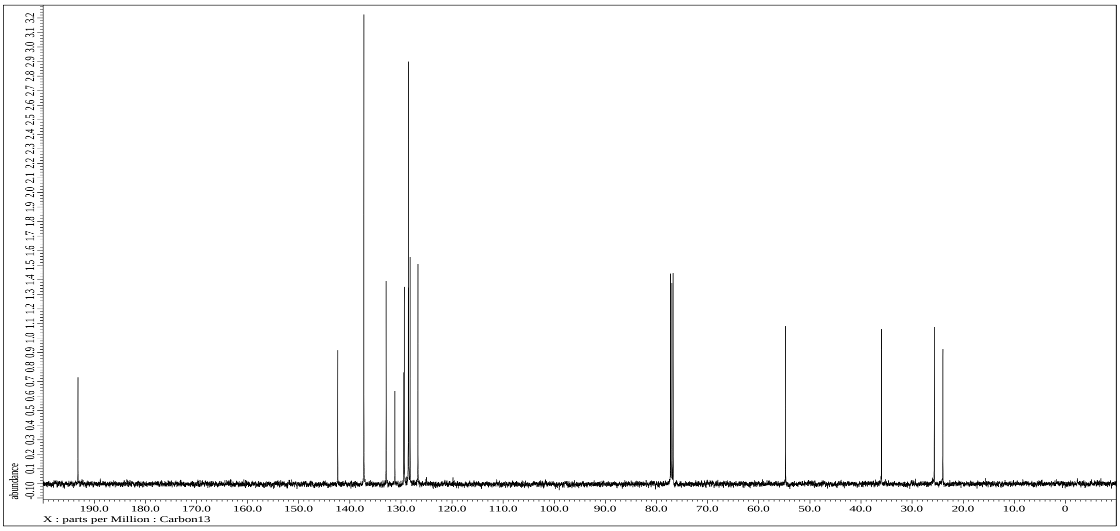

Supplementary Figure 131. ^{13}C NMR spectrum for α-sulfenylketone **9a**.

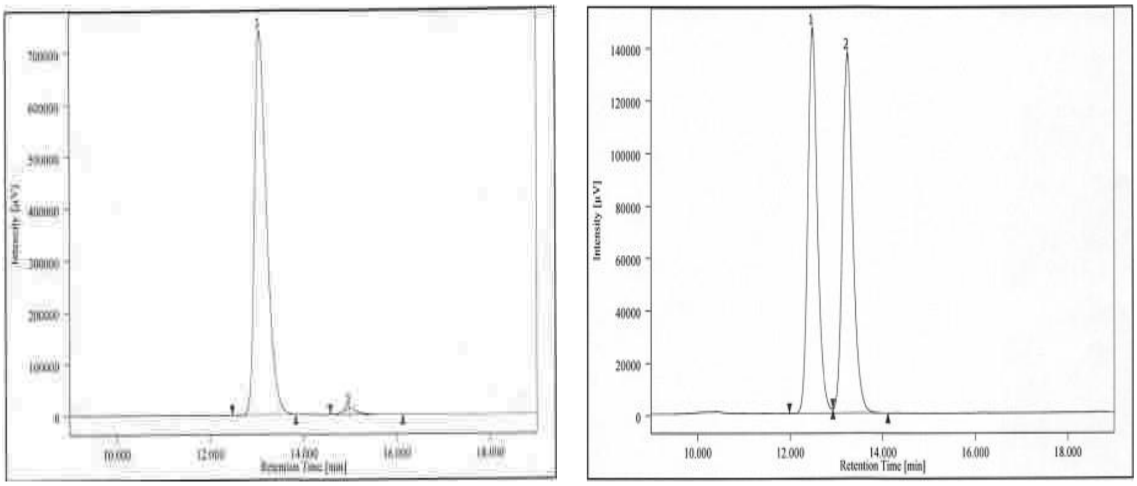

Peak	Retention Time [min]	Area [%]
1	13.1	98.170
2	15.0	1.830

Peak	Retention Time [min]	Area [%]
1	12.5	49.924
2	13.2	50.076

Supplementary Figure 132. HPLC spectra for α-sulfenylketone **9a**.

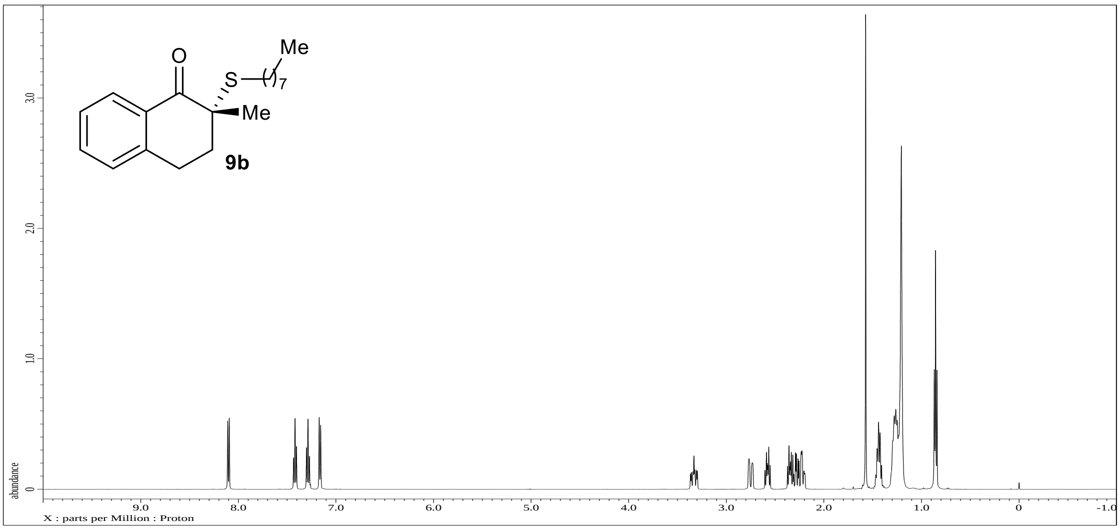

Supplementary Figure 133. ^{1}H NMR spectrum for α-sulfenylketone **9b**.

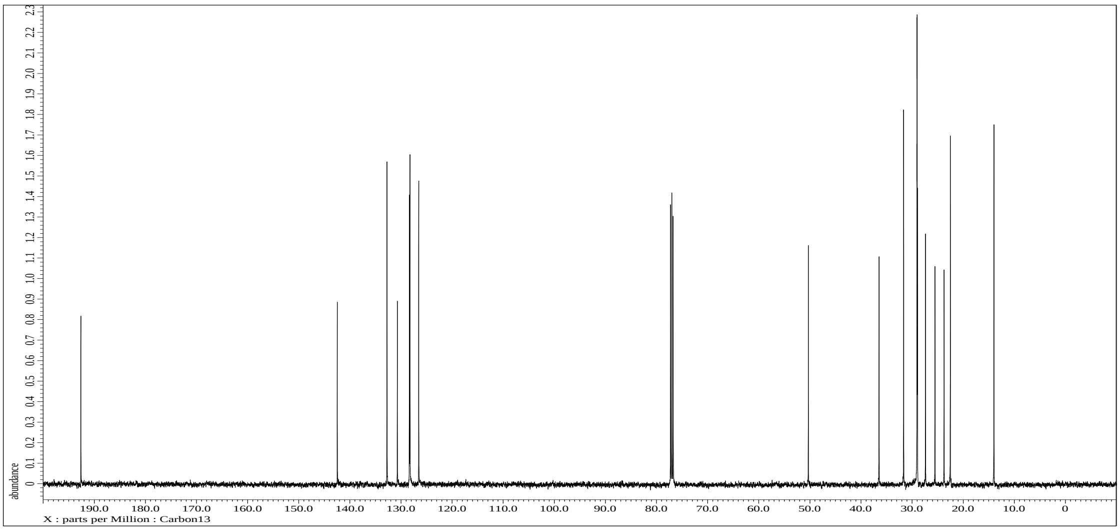

Supplementary Figure 134. ^{13}C NMR spectrum for α-sulfenylketone **9b**.

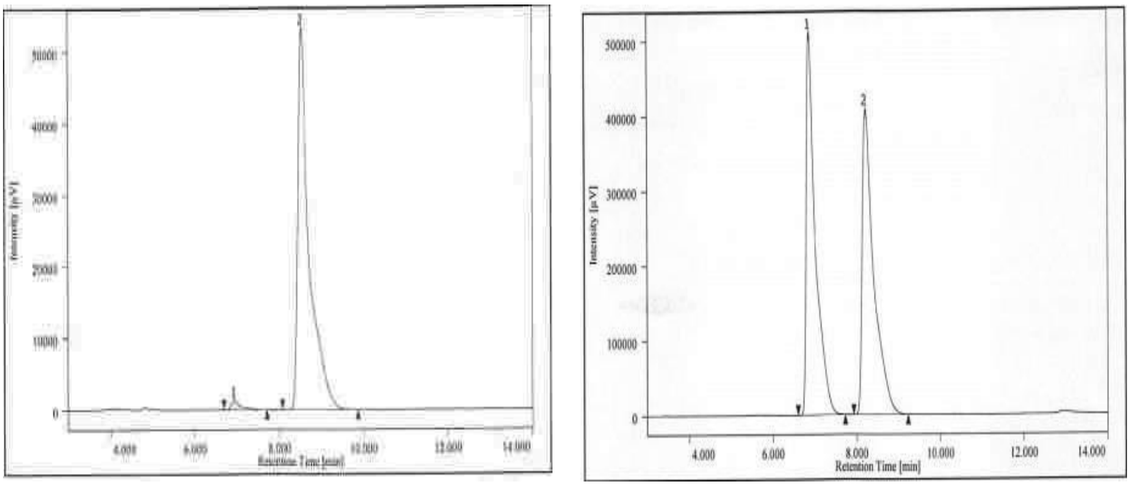

Peak	Retention Time [min]	Area [%]
1	6.9	1.866
2	8.5	98.134

Peak	Retention Time [min]	Area [%]
1	6.9	49.904
2	8.2	50.096

Supplementary Figure 135. HPLC spectra for α-sulfenylketone **9b**.

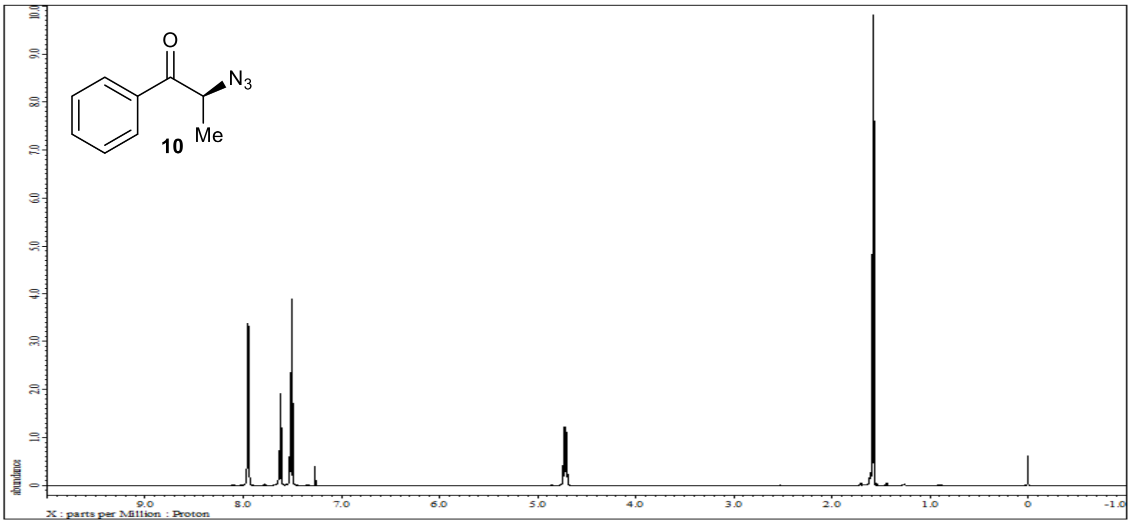

Supplementary Figure 136. ^{1}H NMR spectrum for α-azideketone **10**.

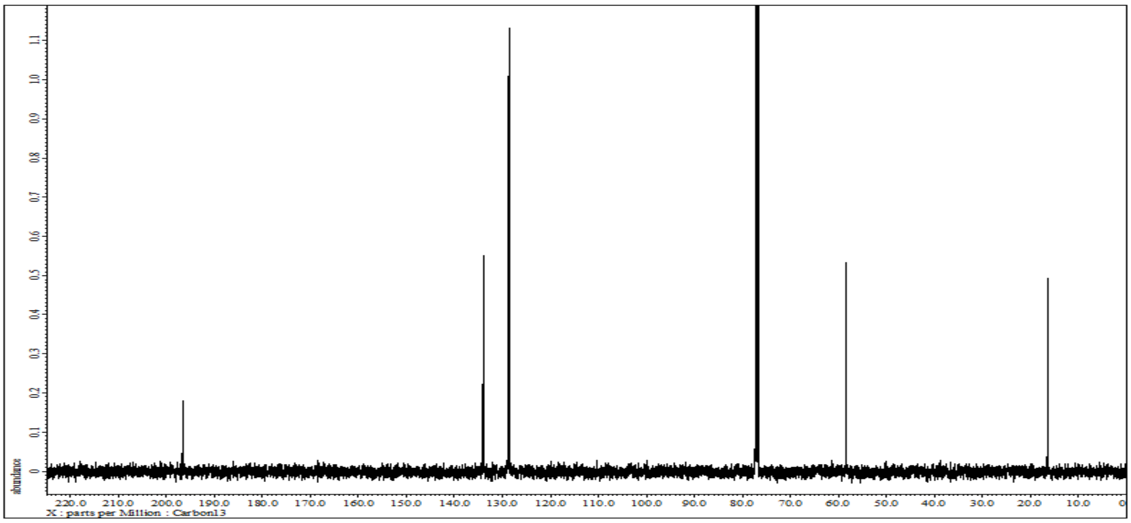

Supplementary Figure 137. ^{13}C NMR spectrum for α-azideketone **10**.

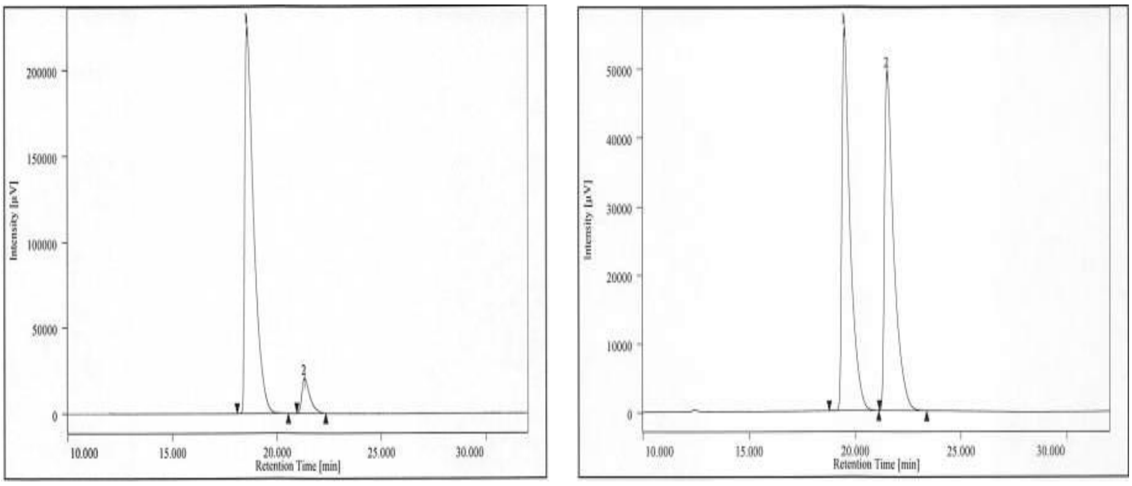

Peak	Retention Time [min]	Area [%]
1	18.6	92.391
2	21.3	7.609

Peak	Retention Time [min]	Area [%]
1	19.5	49.951
2	21.5	50.049

Supplementary Figure 138. HPLC spectra for α-azideketone **10**.

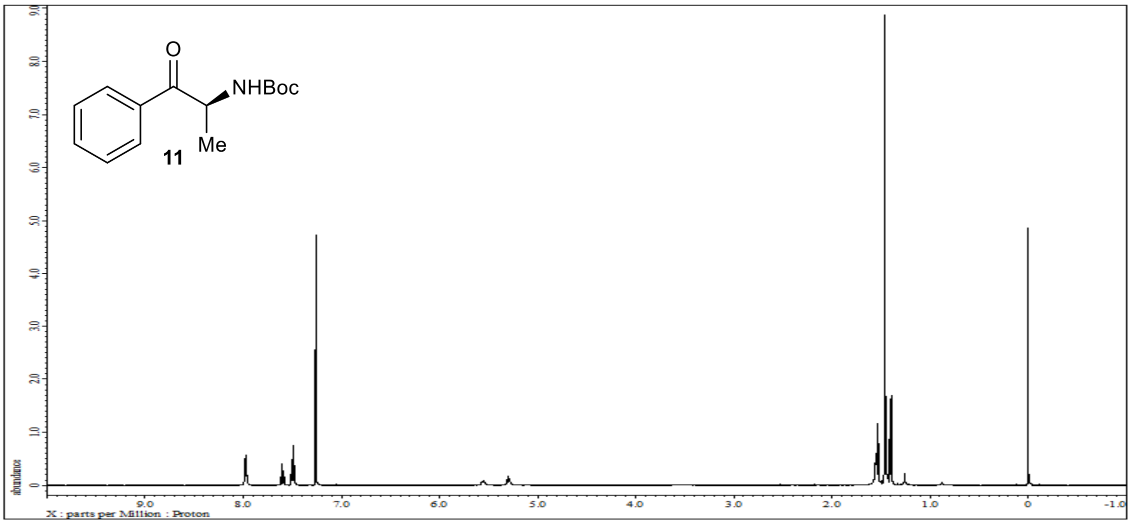

Supplementary Figure 139. ^{1}H NMR spectrum for *N*-Boc Cathinone **11**.

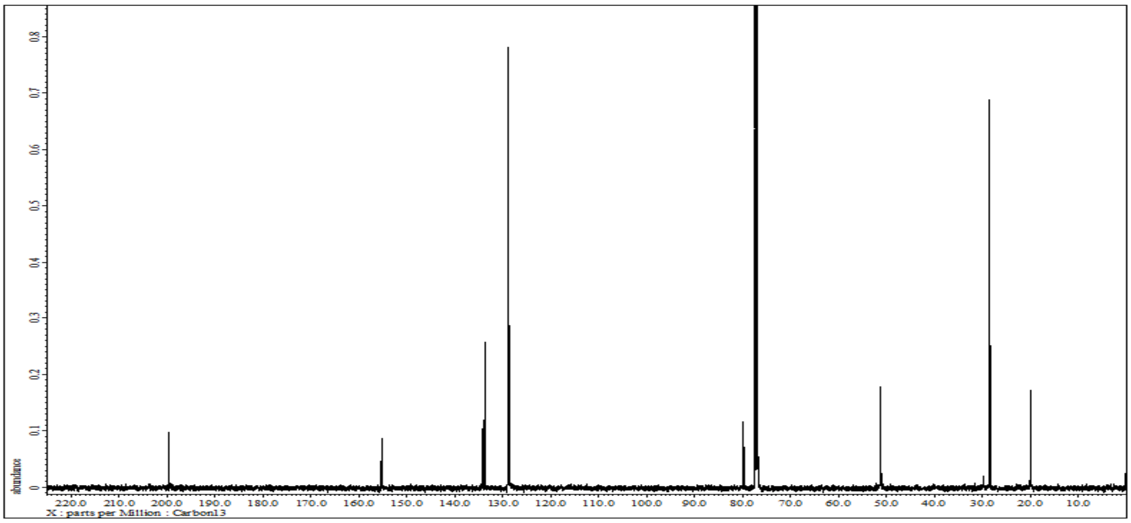

Supplementary Figure 140. ^{13}C NMR spectrum for *N*-Boc Cathinone **11**.

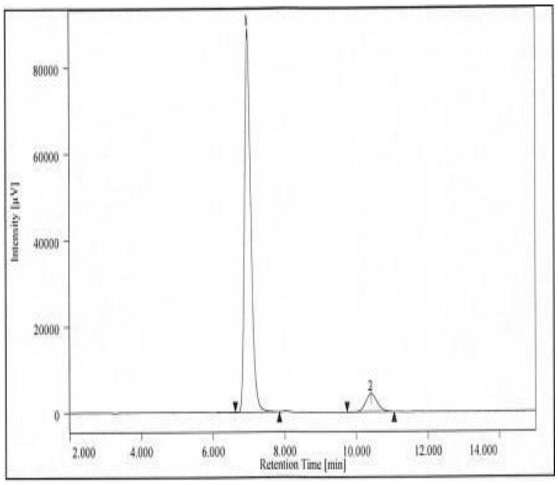

Peak	Retention Time [min]	Area [%]
1	7.0	92.212
2	10.4	7.788

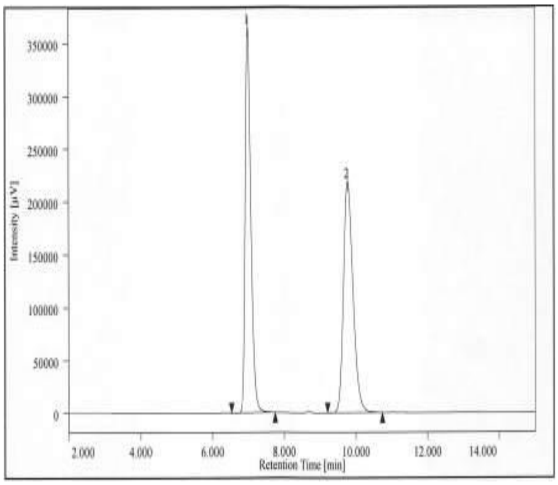

Peak	Retention Time [min]	Area [%]
1	7.0	49.789
2	9.8	50.211

Supplementary Figure 141. HPLC spectra for *N*-Boc Cathinone **11**.

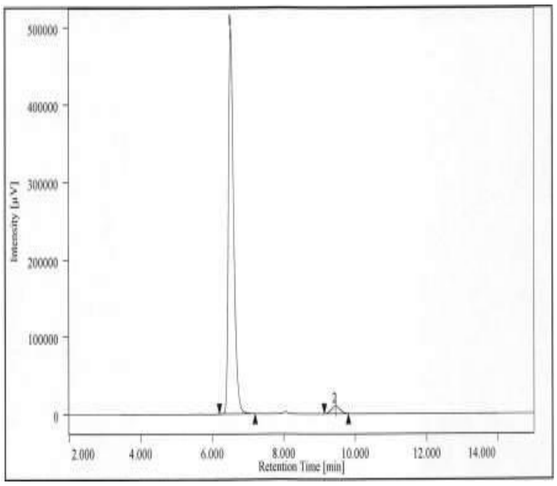

Peak	Retention Time [min]	Area [%]
1	6.5	96.863
2	9.5	3.137

Supplementary Figure 142. HPLC spectra for *N*-Boc Cathinone **11** (after recrystallization).

Supplementary Table 1. Optimization of reaction conditions

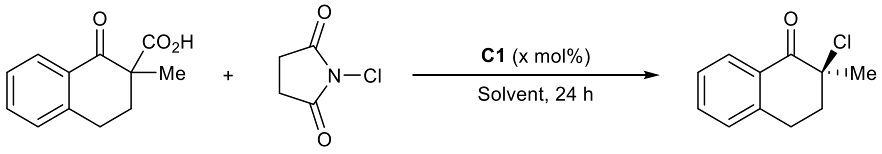

1a *Racemate*   NCS (y equiv.)   **2a**

Entry	x [mol%]	y [equiv.]	Solvent	Temp [°C]	Yield [%]*	e.e. [%]†
1	10	3.0	Toluene	25	99	94
2	10	3.0	Acetonitrile	25	85	84
3	10	3.0	Tetrahydrofuran	25	19	66
4	10	3.0	Dichloromethane	25	91	91
5	10	1.5	Toluene	15	94	96
6‡	5	1.5	Toluene	15	97	95
7‡	2.5	1.5	Toluene	15	79	94
8§	10	1.5	Toluene	15	81	96

* Isolated yield of the purified compound **2a**.

† Determined by chiral HPLC.

‡ The reactions time was 48 h.

§ The reaction performed in the presence of cyclohexane carboxylic acid (1.0 equiv.).

Supplementary Table 2. Optimization of addition rate of NCS

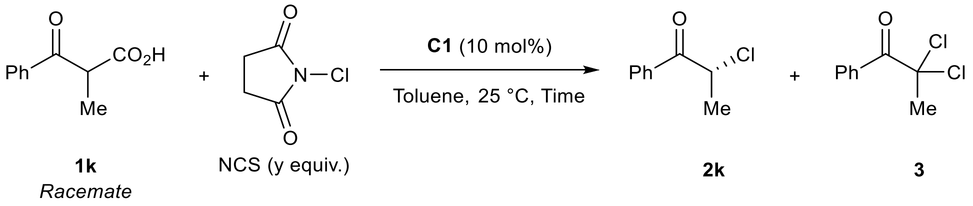

Entry	y [equiv.]	Time	Yield (**2k**) [%]	e.e. (**2k**) [%][*]	Yield (**3**) [%][†]
1	3.0	0.5	18	83	74
2	1.2	0.5	38	83	31
3[‡]	1.2	1	87	85	<5

[*] Isolated yield of the purified compound.

[†] Determined by chiral HPLC.

[‡] The solution of NCS in toluene was added slowly over 1 h using a syringe pump.

Supplementary Table 3. Crystal data and structure refinement for **2a**

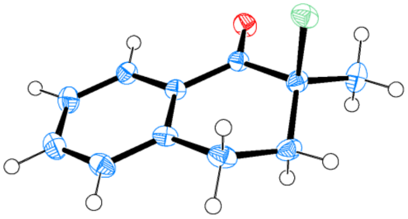

**Deposition number**	CCDC 1516051	
**Empirical formula**	C11 H11 Cl O	
**Formula weight**	194.65	
**Temperature**	120(2) K	
**Wavelength**	0.71069 Å	
**Crystal system**	Orthorhombic	
**Space group**	P $2_12_12_1$	
**Unit cell dimensions**	a = 6.2460(10) Å	α= 90°.
	b = 9.5642(16) Å	β= 90°.
	c = 16.163(3) Å	γ = 90°.
**Volume**	965.6(3) $Å^3$	
**Z**	4	
**Density (calculated)**	1.339 Mg/m^3	
**Absorption coefficient**	0.350 mm^{-1}	
**F(000)**	408	
**Crystal size**	0.50 x 0.30 x 0.30 mm^3	
**Theta range for data collection**	2.47 to 33.98°.	
**Index ranges**	-9<=h<=6, -14<=k<=12, -24<=l<=25	
**Reflections collected**	11097	

**Independent reflections**	3649 [R(int) = 0.0246]
**Completeness to theta = 33.98°**	97.6 %
**Max. and min. transmission**	0.9024 and 0.8446
**Refinement method**	Full-matrix least-squares on F^2
**Data / restraints / parameters**	3649 / 0 / 162
**Goodness-of-fit on F^2**	1.070
**Final R indices [I>2sigma(I)]**	R1 = 0.0281, wR2 = 0.0747
**R indices (all data)**	R1 = 0.0297, wR2 = 0.0765
**Absolute structure parameter**	0.05(4)
**Largest diff. peak and hole**	0.281 and -0.246 e.Å^{-3}
**Hydrogen treatment**	refine all parameters

Supplementary Table 4. Crystal data and structure refinement for **9a**

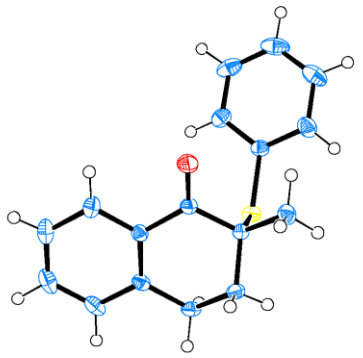

**Deposition number**	CCDC 1516052	
**Empirical formula**	C17 H16 O S	
**Formula weight**	268.36	
**Temperature**	120(2) K	
**Wavelength**	0.71069 Å	
**Crystal system**	Monoclinic	
**Space group**	P 2_1	
**Unit cell dimensions**	a = 8.1745(7) Å	α= 90°.
	b = 12.6892(7) Å	β= 96.208(3)°.
	c = 13.4055(9) Å	γ = 90°.
**Volume**	1382.37(17) Å^3	
**Z**	4	
**Density (calculated)**	1.289 Mg/m^3	
**Absorption coefficient**	0.223 mm^{-1}	
**F(000)**	568	
**Crystal size**	0.30 x 0.30 x 0.20 mm^3	
**Theta range for data collection**	1.53 to 34.00°.	
**Index ranges**	-10<=h<=12,	

	-19<=k<=19, -21<=l<=21
**Reflections collected**	33516
**Independent reflections**	10847 [R(int) = 0.0256]
**Completeness to theta = 33.98°**	99.0 %
**Max. and min. transmission**	0.9568 and 0.9362
**Refinement method**	Full-matrix least-squares on F^2
**Data / restraints / parameters**	10847 / 1 / 471
**Goodness-of-fit on F^2**	1.045
**Final R indices [I>2sigma(I)]**	R1 = 0.0347, wR2 = 0.0830
**R indices (all data)**	R1 = 0.0383, wR2 = 0.0861
**Absolute structure parameter**	0.01(3)
**Largest diff. peak and hole**	0.327 and -0.204 e.$Å^{-3}$
**Hydrogen treatment**	refine all parameters

Supplementary Methods

General. All non-aqueous reactions were carried out in dried glassware under an argon atmosphere and stirred using magnetic stir-plates. Thin-layer chromatography analyses were performed using pre-coated silica gel plates with a fluorescent indicator (F254) (Merck Millipore, Darmstadt, Germany). Visualization was accomplished by ultraviolet (UV) light (254 nm), phosphomolybdic acid, or *p*-anisaldehyde. Flash column chromatography was performed using silica gel 60 (mesh size 40–100) supplied by Kanto Chemical Co., Inc. (Tokyo, Japan). ^{1}H, ^{13}C, and ^{19}F nuclear magnetic resonance (NMR) spectra were recorded on a JNM-ECS400 (400 MHz ^{1}H, 100 MHz ^{13}C, 376 MHz ^{19}F) or a JNM-ECX500 (500 MHz ^{1}H, 126 MHz ^{13}C, 470 MHz ^{19}F) instrument (JEOL Ltd., Tokyo, Japan). Chemical shift values (δ) are reported in ppm (tetramethylsilane δ 0.00 ppm or residual acetone δ 2.05 for ^{1}H; hexafluorobenzene δ −162.2 ppm for ^{19}F; residual chloroform δ 77.0 ppm or acetone δ 29.8 ppm for ^{13}C). Infrared (IR) spectra were recorded on an FT/IR-4600 instrument (JASCO Co., Ltd., Tokyo, Japan). Direct analyses in real time (DART) mass (positive mode) analyses were performed on a JMS-T100TD time-of-flight mass spectrometer (JEOL Ltd.). Melting points were recorded on a YANACO MP-500P micro melting point apparatus (Japan). Optical rotations were measured on a P-1030 digital polarimeter (JASCO Co., Ltd.). Analytical high-performance liquid chromatography (HPLC) was performed on a PU1586 instrument with a MD-2018 plus diode array detector (JASCO Co., Ltd.) using a chiral column under the conditions described below. The enantiomeric purity of the compounds was determined by HPLC analyses using chiral stationary phase columns.

Materials. Commercial grade reagents and solvents were used without further purification unless otherwise noted. Anhydrous acetonitrile, ethyl acetate, dimethylformamide (DMF), and dimethyl sulfoxide (DMSO) were purchased from Sigma-Aldrich (St. Louis, MO). Anhydrous toluene, dichloromethane, tetrahydrofurane (THF) were purchased from Kanto Chemical Co., Inc. and used after purification by a Glass Contour solvent dispensing system (Pure Process Technology, Nashua, NH). Amine catalysts **C1**,[1] **C2**,[1] **C3**,[2] and **C7**[3] and chiral ligand **L1**[2] were prepared by following the reported procedure. Amine catalysts **C4** and **C8** were purchased from Sigma-Aldrich (St. Louis, MO), Amine catalysts **C5** and **C6** were purchased from Tokyo Chemical Industry Co., Ltd (Tokyo, Japan). β-Ketocarboxylic acids **1** were synthesized by acidolysis of the corresponding *tert*-butyl β-ketoesters **12**.

Synthesis of β-ketoesters 12.

β-Ketoesters **12** were synthesised by either **Method A** or **Method B** described below, except for

12i.

[Method A]: Alkylation of active methine/methylene compounds

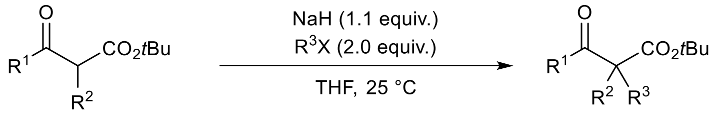

12

(X = Br or I, *t*Bu = *tert*-butyl group)

To a stirred suspension of NaH (60% in oil, washed with hexane, 1.1 equiv.) in THF (20 mL) was added a solution of β-ketoester in THF at 0 °C, and the mixture was stirred at 0 °C for 1 h. Then, alkyl halide (2.0 equiv.) was added, and the reaction mixture was stirred at 25 °C. The reaction mixture was quenched by adding saturated NH_4Cl aqueous solution at 0 °C, and then extracted with ethyl acetate. The combined organic layer was dried over anhydrous Na_2SO_4, concentrated, and then purified by flash column chromatography on silica gel to give alkylated β-ketoester **12**.

[Method B]: Aldol reaction and the following oxidation

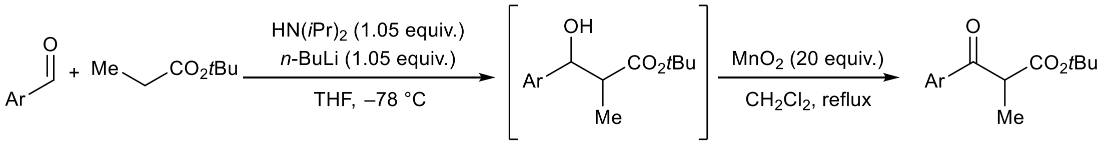

13 1.05 equiv. **14** **12**

(Ar = aryl group, *i*Pr = isopropyl group, *n*-Bu = *n*-butyl group)

To a stirred solution of diisopropyl amine (1.05 equiv.) in THF was added a solution of *n*-butyl lithium in 1.6 M hexane (1.05 equiv.) at −78 °C. The solution was stirred at −78 °C for 1 h. Then, *tert*-butyl ester **13** was added dropwise. After stirring for 30 min, aldehyde was added, and the reaction mixture was stirred at −78 °C. The reaction mixture was quenched by adding saturated NH_4Cl aqueous solution at 0 °C, and then extracted with diethyl ether. The combined organic layer was dried over anhydrous Na_2SO_4, concentrated, and then purified by short column chromatography on silica gel to give β-hydroxy ester **14**.

14 was dissolved in dichloromethane, and manganese (IV) oxide (20 equiv.) was added. The reaction mixture was stirred under reflux conditions. The reaction mixture was cooled to 0 °C, and filtered. The filtrate was concentrated, and then purified by flash column chromatography on silica gel to give β-ketoester **12**.

***tert*-butyl 2-methyl-1-oxo-1,2,3,4-tetrahydronaphthalene-2-carboxylate (12a)**

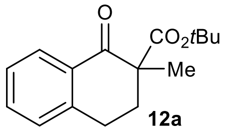

The title compound was prepared following **Method A**, using NaH (185 mg, 7.70 mmol), *tert*-butyl 1-oxo-1,2,3,4-tetrahydronaphthalene-2-carboxylate (1.73 g, 7.00 mmol), and iodomethane (1.99 g, 14.0 mmol) in THF (25 mL), and the reaction mixture was stirred for 1 h. The combined organic layer was dried over anhydrous $MgSO_4$. The crude product was purified by flash column chromatography (hexane : ethyl acetate = 10 : 1) to provide the title compound as a colourless oil (1.71 g, 94% yield).

TLC (hexane : ethyl acetate = 9 : 1): R_f = 0.43; **^{1}H NMR** (400 MHz, $CDCl_3$): δ 8.03 (d, *J* = 7.9 Hz, 1H), 7.44 (t, *J* = 7.3 Hz, 1H), 7.29 (t, *J* = 7. 6 Hz, 1H), 7.21 (d, *J* = 7.6 Hz, 1H), 3.10–3.02 (m, 1H), 2.94–2.88 (m, 1H), 2.56–2.51 (m, 1H), 2.05–1.96 (m, 1H), 1.45 (s, 3H), 1.34 (s, 9H); **^{13}C NMR** (100 MHz, $CDCl_3$): δ 196.2, 171.9, 142.7, 132.9, 132.0, 128.4, 127.5, 126.4, 81.5, 54.1, 34.0, 27.5, 25.9, 20.3; **IR** (neat): 2973, 2934, 1729, 1690, 1603, 1458, 1371, 1310, 1256, 1230, 1158, 1116, 740 cm^{-1}; **HRMS** (DART): $[M+H]^+$calcd. for $C_{16}H_{21}O_3$, 261.1491; found, 261.1492.

***tert*-butyl 2-allyl-1-oxo-1,2,3,4-tetrahydronaphthalene-2-carboxylate (12b)**

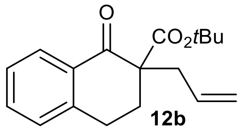

The title compound was prepared following **Method A**, using NaH (59.0 mg, 2.48 mmol), *tert*-butyl 1-oxo-1,2,3,4-tetrahydronaphthalene-2-carboxylate (555 mg, 2.25 mmol), and allyl bromide (327 mg, 2.70 mmol) in THF (8 mL), and the reaction mixture was stirred for 24 h. The combined organic layer was dried over anhydrous $MgSO_4$. The crude product was purified by flash column chromatography (hexane : ethyl acetate = 20 : 1) to provide the title compound as a colourless oil (523 mg, 81% yield).

TLC (hexane : ethyl acetate = 4 : 1): R_f = 0.50; **^{1}H NMR** (500 MHz, $CDCl_3$): δ 8.03 (dd, *J* = 7.6, 1.2 Hz, 1H), 7.44 (dt, *J* = 7.6, 1.5 Hz, 1H), 7.28 (t, *J* = 7.6 Hz, 1H), 7.20 (d, *J* = 7.6 Hz, 1H), 5.87 (ddt, *J* = 17.2, 9.9, 7.3 Hz, 1H), 5.16–5.11 (m, 1H), 5.10–5.07 (m, 1H), 3.09 (ddd, *J* = 16.4, 10.7, 4.6 Hz, 1H), 2.91 (dt, *J* = 17.6, 4.6 Hz, 1H), 2.67 (d, *J* = 7.3 Hz, 2H), 2.45 (dt, *J* = 13.8, 4.6 Hz, 1H), 2.11 (ddd, *J* = 13.9, 10.8, 5.0 Hz, 1H), 1.34 (s, 9H); **^{13}C NMR** (126 MHz, $CDCl_3$): δ 195.3, 170.6, 142.7, 133.6, 133.0, 132.4, 128.5, 127.6, 126.5, 118.4, 81.8, 57.5, 38.5, 30.7, 27.6, 25.8; **IR** (neat): 3075, 2977, 2931, 1729, 1694, 1599, 1454, 1367, 1249, 1154, 926, 740

cm^{-1}; **HRMS** (DART): $[M+H]^+$calcd. for $C_{18}H_{23}O_3$, 287.1647; found, 287.1649.

***tert*-butyl 2-benzyl-1-oxo-1,2,3,4-tetrahydronaphthalene-2-carboxylate (12c)**[4]

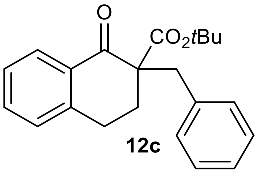

The title compound was prepared following **Method A**, using NaH (53.0 mg, 2.22 mmol), *tert*-butyl 1-oxo-1,2,3,4-tetrahydronaphthalene-2-carboxylate (496 mg, 2.01 mmol), and benzyl bromide (689 mg, 4.03 mmol) in THF (7.1 mL), and the reaction mixture was stirred for 13 h. The combined organic layer was dried over anhydrous $MgSO_4$. The crude product was purified by flash column chromatography (hexane : ethyl acetate = 10 : 1 to 5 : 1) to provide the title compound as a colourless oil (568 mg, 84% yield).

***tert*-butyl 2-(3-chloropropyl)-1-oxo-1,2,3,4-tetrahydronaphthalene-2-carboxylate (12d)**

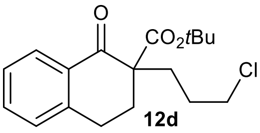

The title compound was prepared following **Method A**, using NaH (43.8 mg, 1.83 mmol), *tert*-butyl 1-oxo-1,2,3,4-tetrahydronaphthalene-2-carboxylate (300 mg, 1.22 mmol), and 1-chloro-3-iodopropane (374 mg, 1.83 mmol) in THF (4.3 mL), and the reaction mixture was stirred for 30 h. The reaction mixture was extracted with diethyl ether. The crude product was purified by flash column chromatography (hexane : diethyl ether = 95 : 5 to 80 : 20) to provide the title compound as a colourless oil (72.8 mg, 19% yield).

TLC (hexane : ethyl acetate = 4 : 1): R_f = 0.47; **^{1}H NMR** (500 MHz, $CDCl_3$): δ 8.01 (dd, *J* = 7.8, 1.5 Hz, 1H), 7.46 (dt, *J* = 7.5, 1.5 Hz, 1H), 7.30 (t, *J* = 7.5 Hz, 1H), 7.21 (d, *J* = 7.6 Hz, 1H), 3.61–3.54 (m, 2H), 3.09 (ddd, *J* = 16.9, 11.1, 4.8 Hz, 1H), 2.92 (dt, *J* = 17.6, 4.6 Hz, 1H), 2.50 (dt, *J* = 13.8, 4.6 Hz, 1H), 2.13–1.95 (m, 4H), 1.89–1.78 (m, 1H), 1.34 (s, 9H); **^{13}C NMR** (126 MHz, $CDCl_3$): δ 195.8, 170.9, 142.6, 133.1, 132.5, 128.5, 127.7, 126.6, 82.2, 57.4, 45.2, 31.6, 31.3, 28.1, 27.7, 26.0; **IR** (neat): 2973, 2934, 1729, 1690, 1599, 1454, 1371, 1253, 1230, 1154, 910, 842, 740 cm^{-1}; **HRMS** (DART): $[M+H]^+$calcd. for $C_{18}H_{24}Cl_1O_3$, 323.1414; found, 323.1414.

tert-butyl 2-(2-cyanoethyl)-1-oxo-1,2,3,4-tetrahydronaphthalene-2-carboxylate (12e)

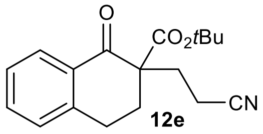

The title compound was prepared following **Method A**, using NaH (74.4 mg, 3.10 mmol), *tert*-butyl 1-oxo-1,2,3,4-tetrahydronaphthalene-2-carboxylate (382 mg, 1.55 mmol), and 3-bromopropionitrile (415 mg, 3.10 mmol) in THF (5.5 mL), and the reaction mixture was stirred for 6 days. The reaction mixture was extracted with diethyl ether. The crude product was purified by flash column chromatography (hexane : ethyl acetate = 4 : 1) to provide the title compound as a colourless oil (282 mg, 61% yield).

TLC (hexane : ethyl acetate = 4 : 1): R_f = 0.30; **^{1}H NMR** (500 MHz, $CDCl_3$): δ 7.99 (dd, *J* = 8.0, 1.2 Hz, 1H), 7.48 (dt, *J* = 7.6, 1.5 Hz, 1H), 7.31 (t, *J* = 7.5 Hz, 1H), 7.23 (d, *J* = 7.6 Hz, 1H), 3.08 (ddd, *J* = 17.1, 11.4, 4.8 Hz, 1H), 2.95 (dt, *J* = 17.6, 4.6 Hz, 1H), 2.75 (ddd, *J* = 13.8, 10.5, 5.7 Hz, 1H), 2.51–2.43 (m, 2H), 2.23 (dddd, *J* = 26.0, 14.1, 10.2, 5.7 Hz, 2H), 2.11 (ddd, *J* = 13.5, 11.0, 5.0 Hz, 1H), 1.33 (s, 9H); **^{13}C NMR** (126 MHz, $CDCl_3$): δ 195.0, 169.9, 142.1, 133.4, 132.0, 128.5, 127.4, 126.7, 119.5, 82.9, 56.6, 31.8, 30.1, 27.5, 25.7, 13.1; **IR** (neat): 3066, 2977, 2921, 2861, 2248, 1730, 1686, 1606, 1451, 1376, 1253, 1148, 1095, 905, 846, 742 cm^{-1}; **HRMS** (DART): $[M+H]^+$calcd. for $C_{18}H_{22}N_1O_3$, 300.1600; found, 300.1601.

tert-butyl 6-chloro-3-methyl-4-oxochromane-3-carboxylate (12f)

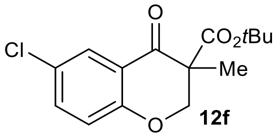

The title compound was prepared following **Method A**, using NaH (12.6 mg, 0.527 mmol), *tert*-butyl 6-chloro-4-oxochromane-3-carboxylate (135 mg, 0.479 mmol), and iodomethane (81.5 mg, 0.574 mmol) in THF (1.0 mL), and the reaction mixture was stirred for 21 h. The reaction mixture was extracted with diethyl ether. The crude product was purified by flash column chromatography (hexane : ethyl acetate = 20 : 1) to provide the title compound as a white solid (116 mg, 82% yield).

mp: 83 °C; **TLC** (hexane : ethyl acetate = 9 : 1): R_f = 0.50; **^{1}H NMR** (500 MHz, $CDCl_3$): δ 7.87 (d, *J* = 2.7 Hz, 1H), 7.41 (dd, *J* = 8.8, 2.7 Hz, 1H), 6.94 (d, *J* = 9.2 Hz, 1H), 4.74 (d, *J* = 11.5 Hz, 1H), 4.15 (d, *J* = 11.5 Hz, 1H), 1.39 (s, 3H), 1.35 (s, 9H); **^{13}C NMR** (126 MHz, $CDCl_3$): δ 189.6, 169.4, 159.4, 135.4, 127.0, 126.7, 121.2, 119.2, 82.9, 74.1, 53.6, 27.6, 15.3; **IR** (neat): 2981, 2935, 1731, 1704, 1607, 1476, 1422, 1372, 1283, 1249, 1133, 1029, 836, 821, 682, 643 cm^{-1}; **HRMS** (DART): $[M+H]^+$calcd. for $C_{15}H_{18}Cl_1O_4^{+1}$, 297.0894; found, 297.0894.

tert-butyl 2-methyl-1-oxo-2,3-dihydro-1_H_-indene-2-carboxylate (12g)[5]

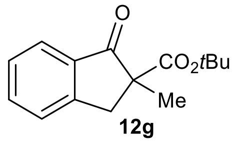

12g

The title compound was prepared following **Method A**, using NaH (114 mg, 4.74 mmol), *tert*-butyl 1-oxo-2,3-dihydro-1*H*-indene-2-carboxylate (1.00 g, 4.31 mmol), and iodomethane (1.22 g, 8.61 mmol) in THF (15 mL), and the reaction mixture was stirred for 1 h. The combined organic layer was dried over anhydrous $MgSO_4$. The crude product was purified by flash column chromatography (hexane : ethyl acetate = 20 : 1 to 5 : 1) to provide the title compound as a white solid (937 mg, 88% yield).

tert-butyl 5-bromo-2-methyl-1-oxo-2,3-dihydro-1_H_-indene-2-carboxylate (12h)

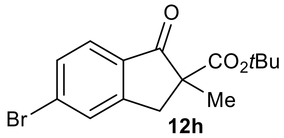

12h

The title compound was prepared following **Method A**, using NaH (89.0 mg, 3.70 mmol), *tert*-butyl 5-bromo-1-oxo-2,3-dihydro-1*H*-indene-2-carboxylate (960 mg, 3.09 mmol), and iodomethane (569 mg, 4.01 mmol) in THF (11 mL), and the reaction mixture was stirred for 13 h. The combined organic layer was dried over anhydrous $MgSO_4$. The crude product was purified by flash column chromatography (hexane : ethyl acetate = 20 : 1 to 5 : 1) to provide the title compound as a white solid (660 mg, 66% yield).

mp: 52 °C; **TLC** (hexane : ethyl acetate = 9 : 1): R_f = 0.40; **^{1}H NMR** (500 MHz, $CDCl_3$): δ 7.65–7.63 (m, 2H), 7.55–7.53 (m, 1H), 3.62 (d, *J* = 17.6 Hz, 1H), 2.94 (d, *J* = 17.6 Hz, 1H), 1.46 (s, 3H), 1.38 (s, 9H); **^{13}C NMR** (126 MHz, $CDCl_3$): δ 202.7, 170.7, 154.2, 133.9, 131.4, 130.5, 129.7, 126.0, 81.9, 56.9, 39.8, 27.8, 20.6; **IR** (neat): 2980, 2934, 1736, 1721, 1599, 1371, 1264, 1203, 1150, 975, 846 cm^{-1}; HRMS (DART): $[M+NH_4]^+$calcd. for $C_{15}H_{21}Br_1N_1O_3$, 342.0705; found, 342.0705.

tert-butyl 2-methyl-3-oxo-2,3-diphenylpropanoate (12i)

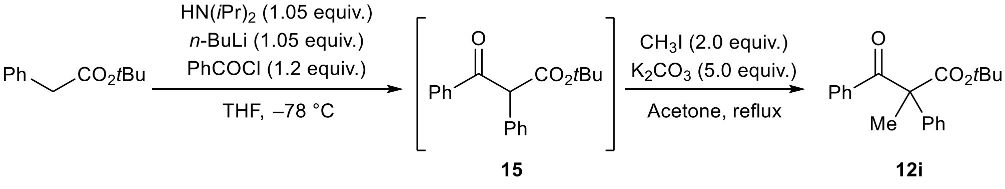

To a stirred solution of diisopropyl amine (1.18 g, 11.1 mmol) in THF (38 mL) was added a solution of *n*-butyl lithium in 1.6 M hexane (6.9 mL, 11.1 mmol) at −78 °C. The solution was

stirred at −78 °C for 1 h. Then, a solution of *tert*-butyl 2-phenylacetate (2.03 g, 10.6 mmol) in THF (15 mL) was added dropwise. After stirring for 30 min, benzoyl chloride (1.78 g, 12.7 mmol) was added, and the reaction mixture was stirred at −78 °C for 1 h. The reaction mixture was quenched by adding saturated NH_4Cl aqueous solution at 0 °C, and then extracted with diethyl ether. The combined organic layer was dried over anhydrous Na_2SO_4, concentrated, and then purified by column chromatography on silica gel (hexane : ethyl acetate = 30 : 1 to 10 : 1) to give *tert*-butyl 3-oxo-2,3-diphenylpropanoate **15** (2.12 g, 68% yield).

TLC (hexane : ethyl acetate = 10 : 1): R_f = 0.39; **^{1}H NMR** (500 MHz, $CDCl_3$): δ 7.97 (d, *J* = 7.6 Hz, 2H), 7.53 (t, *J* = 7.6 Hz, 1H), 7.43 (t, *J* = 7.6 Hz, 2H), 7.40–7.34 (m, 4H), 7.30 (t, *J* = 7.3 Hz, 1H), 5.50 (s, 1H), 1.42 (s, 9H).

To a stirred solution of **15** (1.53 g, 5.16 mmol) in acetone (52 mL) was added iodomethane (1.47 g, 10.3 mmol) and K_2CO_3 (3.57 g, 25.8 mmol). The reaction mixture was stirred under reflux conditions for 12 h. The reaction mixture was cooled to 25 °C and then filtered. The filtrate was concentrated and dissolved in diethyl ether. Then, the organic layer was washed with brine, dried over anhydrous Na_2SO_4, concentrated, and then purified by flash column chromatography on silica gel (hexane : diethyl ether = 20 : 1 to 10 : 1) to give the title compound as a colourless oil (1.32 g, 82% yield).

TLC (hexane : ethyl acetate = 10 : 1): R_f = 0.43; **^{1}H NMR** (500 MHz, $CDCl_3$): δ 7.71 (d, *J* = 7.3 Hz, 2H), 7.48 (d, *J* = 7.3 Hz, 2H), 7.42 (t, *J* = 7.3 Hz, 1H), 7.33–7.24 (m, 5H), 1.86 (s, 3H), 1.25 (s, 9H); **^{13}C NMR** (126 MHz, $CDCl_3$): δ 197.2, 171.2, 140.3, 136.2, 132.2, 129.4, 128.3, 128.0, 127.6, 127.1, 82.1. 62.6, 27.5, 26.1; **IR** (neat): 2978, 2935, 1735, 1692, 1449, 1372, 1264, 1164, 1133, 1094, 967, 693 cm^{-1}; **HRMS** (DART): $[M+H]^+$calcd. for $C_{20}H_{23}O_3$, 311.1647; found, 311.1645.

***tert*-butyl 1-benzyl-2-oxocyclohexane-1-carboxylate (12j)**[5]

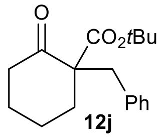

12j

The title compound was prepared following **Method A**, using NaH (19.0 mg, 0.792 mmol), *tert*-butyl 2-oxocyclohexane-1-carboxylate (151 mg, 0.762 mmol), and benzyl bromide (156 mg, 0.910 mmol) in THF (2.7 mL), and the reaction mixture was stirred for 7 h. The reaction mixture was extracted with diethyl ether. The crude product was purified by flash column chromatography (hexane : ethyl acetate = 10 : 1) to provide the title compound as a colourless oil (147 mg, 67% yield).

tert-butyl 2-methyl-3-oxo-3-phenylpropanoate (12k)[6]

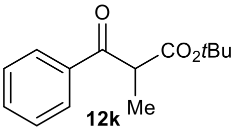

12k

The title compound was prepared following **Method B**, using diisopropyl amine (2.65 g, 26.3 mmol), *n*-butyl lithium in 1.6 M hexane (16.3 mL, 26.3 mmol), *tert*-butyl propionate **13** (3.42 g, 26.3 mmol), and benzaldehyde (2.66 g, 25.0 mmol) in THF (50 mL), and the reaction mixture was stirred for 4 h. The crude product was purified by flash column chromatography (hexane : ethyl acetate = 10 : 1 to 5 : 1) to provide *tert*-butyl 3-hydroxy-2-methyl-3-phenylpropanoate **14k**[7] (4.32 g, 73% yield, dr = 3 : 2).

Oxidation of **14k** (4.32 mg, 18.3 mmol) was carried out with manganese(IV) oxide (31.8 g, 365 mmol) in dichloromethane (61 mL), and the reaction mixture was stirred for 6 h. The crude product was purified by flash column chromatography (hexane : ethyl acetate = 12 : 1 to 5 : 1) to provide the title compound as a colourless oil (2.44 g, 57% yield).

tert-butyl 2-benzoylpent-4-enoate (12l)[8]

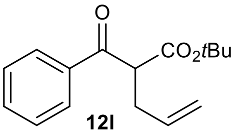

12l

The title compound was prepared following **Method A**, using NaH (46.1 mg, 1.92 mmol), *tert*-butyl 3-oxo-3-phenylpropanoate (352 mg, 1.60 mmol), and allyl bromide (232 mg, 1.92 mmol) in THF (8 mL), and the reaction mixture was stirred for 13 h. The crude product was purified by flash column chromatography (hexane : diethyl ether = 15 : 1 to 10 : 1) to provide the title compound as a colourless oil (247 mg, 59% yield).

tert-butyl 2-benzyl-3-oxo-3-phenylpropanoate (12m)[9]

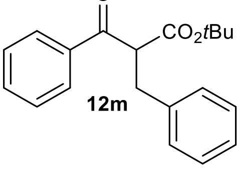

12m

The title compound was prepared following **Method A**, using NaH (52.3 mg, 2.18 mmol), *tert*-butyl 3-oxo-3-phenylpropanoate (400 mg, 1.82 mmol), and benzyl bromide (466 mg, 2.72 mmol) in THF (9 mL), and the reaction mixture was stirred for 13 h. The crude product was purified by flash column chromatography (hexane : diethyl ether = 10 : 1 to 6 : 1) to provide the title compound as a colourless oil (427 mg, 71% yield).

***tert*-butyl 2-methyl-3-oxo-3-(*p*-tolyl)propanoate (12n)**

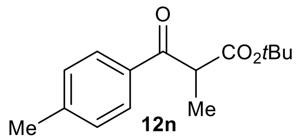

The title compound was prepared following **Method B**, using diisopropyl amine (1.77 g, 17.5 mmol), *n*-butyl lithium in 1.6 M hexane (11 mL, 17.5 mmol), *tert*-butyl propionate **13** (2.28 g, 17.5 mmol), and *p*-tolualdehyde (2.00 g, 16.7 mmol) in THF (33 mL), and the reaction mixture was stirred for 16 h. The crude product was purified by flash column chromatography (hexane : ethyl acetate = 20 : 1 to 10 : 1) to provide *tert*-butyl 3-hydroxy-3-(*p*-tolyl)-2- methylpropanoate **14n**[10] (2.74 g, 61% yield, dr = 3 : 2).

Oxidation of **14n** (2.74 g, 10.2 mmol) was carried out with manganese(IV) oxide (17.6 g, 203 mmol) in dichloromethane (34 mL), and the reaction mixture was stirred for 24 h. The crude product was purified by flash column chromatography (hexane : ethyl acetate = 20 : 1) to provide the title compound as a colourless oil (1.50 g, 55% yield).

TLC (hexane : ethyl acetate = 10 : 1): R_f = 0.36; **^{1}H NMR** (500 MHz, $CDCl_3$): δ 7.89–7.87 (m, 2H), 7.27–7.26 (m, 2H), 4.24 (q, *J* = 7.3 Hz, 1H), 2.41 (s, 3H), 1.44 (d, *J* = 7.3 Hz, 3H), 1.36 (s, 9H); **^{13}C NMR** (126 MHz, $CDCl_3$): δ 195.7, 170.1, 144.0, 133.6, 129.2, 128.6, 81.5, 49.3, 27.7, 21.5, 13.5; **IR** (neat): 2978, 2939, 1731, 1685, 1607, 1453, 1372, 1241, 1148, 963, 851, 736 cm^{-1}; **HRMS** (DART): $[M+H]^+$calcd. for $C_{15}H_{21}O_3$, 249.1491; found, 249.1490.

***tert*-butyl 3-(4-fluorophenyl)-2-methyl-3-oxopropanoate (12o)**

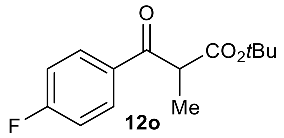

The title compound was prepared following **Method B**, using diisopropyl amine (1.71 g, 16.9 mmol), *n*-butyl lithium in 1.6 M hexane (11 mL, 16.9 mmol), *tert*-butyl propionate **13** (2.20 g, 16.9 mmol), and 4-fluorobenzaldehyde (2.00 g, 16.1 mmol) in THF (32 mL), and the reaction mixture was stirred for 19 h. The crude product was purified by flash column chromatography (hexane : ethyl acetate = 15 : 1 to 10 : 1) to provide *tert*-butyl 3-hydroxy-3-(4-fluorophenyl)-2-methylpropanoate **14o** (2.94 g, 72% yield, dr = 1 : 1).

TLC (hexane : ethyl acetate = 3 : 1): R_f = 0.41; **^{1}H NMR** (500 MHz, $CDCl_3$): δ 7.34–7.30 (m, 2H), 7.06–7.00 (m, 2H), 5.01 (t, *J* = 3.4 Hz, 0.5H), 4.69 (dd, *J* = 5.0, 8.0 Hz, 0.5H), 3.29 (d, *J* = 5.0 Hz, 0.5H), 3.15 (d, *J* = 2.7 Hz, 0.5H), 2.70–2.61 (m, 1H), 1.44 (s, 4.5H), 1.41 (s, 4.5H), 1.09 (d, *J* = 7.3 Hz, 1.5H), 1.02 (d, *J* = 7.3 Hz, 1.5H); **^{19}F NMR** (470 MHz, $CDCl_3$): δ –115.2, –115.8.

Oxidation of **14o** (2.94 g, 11.5 mmol) was carried out with manganese(IV) oxide (20.0 g, 231 mmol) in dichloromethane (38 mL), and the reaction mixture was stirred for 16 h. The crude product was purified by flash column chromatography (hexane : ethyl acetate = 20 : 1) to provide the title compound as a colourless oil (1.65 g, 57% yield).

TLC (hexane : ethyl acetate = 10 : 1): R_f = 0.38; **^{1}H NMR** (500 MHz, $CDCl_3$): δ 8.02 (dd, *J* = 8.8, 5.4 Hz, 2H), 7.15 (d, *J* = 8.8 Hz, 2H), 4.23 (q, *J* = 7.3 Hz, 1H), 1.46 (d, *J* = 7.3 Hz, 3H), 1.36 (s, 9H); **^{13}C NMR** (126 MHz, $CDCl_3$): δ 194.4, 169.8, 165.7 (d, *J* = 255.5 Hz), 132.6 (d, *J* = 2.4 Hz), 131.1 (d, *J* = 9.6 Hz), 115.6 (d, *J* = 21.6 Hz), 81.8, 49.5, 27.7, 13.3; **^{19}F NMR** (470 MHz, $CDCl_3$): δ –105.2; **IR** (neat): 2978, 2939, 1735, 1685, 1604, 1511, 1364, 1233, 1160, 963, 848 cm^{-1}; **HRMS** (DART): $[M+H]^+$calcd. for $C_{14}H_{18}F_1O_3$, 253.1240; found, 253.1240.

***tert*-butyl 3-(3-chlorophenyl)-2-methyl-3-oxopropanoate (12p)**

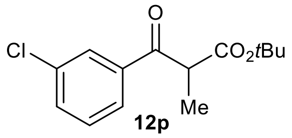

12p

The title compound was prepared following **Method B**, using diisopropyl amine (1.51 g, 14.9 mmol), *n*-butyl lithium in 1.6 M hexane (9.7 mL, 14.9 mmol), *tert*-butyl propionate **13** (1.95 g, 14.9 mmol), and 3-chlorobenzaldehyde (2.00 g, 14.2 mmol) in THF (28 mL), and the reaction mixture was stirred for 22 h. The crude product was purified by flash column chromatography (hexane : ethyl acetate = 12 : 1 to 8 : 1) to provide *tert*-butyl 3-hydroxy-3-(3-chlorophenyl)-2-methylpropanoate **14p** (2.91 g, 76% yield, dr = 1 : 1).

TLC (hexane : ethyl acetate = 8 : 1): R_f = 0.25; **^{1}H NMR** (500 MHz, $CDCl_3$): δ 7.36–7.35 (m, 1H), 7.29–7.21 (m, 3H), 5.03 (t, *J* = 3.4 Hz, 0.5H), 4.68 (dd, *J* = 5.4, 7.6 Hz, 0.5H), 3.36 (d, *J* = 5.4 Hz, 0.5H), 3.24 (d, *J* = 3.4 Hz, 0.5H), 2.71–2.63 (m, 1H), 1.43 (s, 9H), 1.08–1.06 (m, 3H).

Oxidation of **14p** (2.91 g, 10.7 mmol) was carried out with manganese(IV) oxide (18.7 g, 215 mmol) in dichloromethane (36 mL), and the reaction mixture was stirred for 5 h. The crude product was purified by flash column chromatography (hexane : ethyl acetate = 15 : 1) to provide the title compound as a colourless oil (1.86 g, 65% yield).

TLC (hexane : ethyl acetate = 10 : 1): R_f = 0.42; **^{1}H NMR** (500 MHz, $CDCl_3$): δ 7.96 (t, *J* = 1.9 Hz, 1H), 7.86 (ddd, *J* = 8.0, 1.9, 1.2 Hz, 1H), 7.54 (ddd, *J* = 8.0, 1.9, 1.2 Hz, 1H), 7.42 (t, *J* = 8.0 Hz, 1H), 4.22 (q, *J* = 6.9 Hz, 1H), 1.45 (d, *J* = 6.9 Hz, 3H), 1.36 (s, 9H); **^{13}C NMR** (126 MHz, $CDCl_3$): δ 194.7, 169.5, 137.6, 134.8, 133.0, 129.8, 128.5, 126.5, 81.9, 49.5, 27.6, 13.2; **IR** (neat): 2978, 2943, 1735, 1692, 1573, 1457, 1372, 1233, 1148, 1075, 967, 848, 747, 682 cm^{-1}; **HRMS** (DART): $[M+H]^+$calcd. for $C_{14}H_{18}Cl_1O_3$, 269.0945; found, 269.0943.

***tert*-butyl 3-(4-methoxyphenyl)-2-methyl-3-oxopropanoate (12q)**

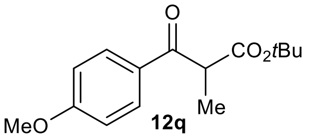

The title compound was prepared following **Method B**, using diisopropyl amine (1.56 g, 15.4 mmol), *n*-butyl lithium in 1.6 M hexane (9.6 mL, 15.4 mmol), *tert*-butyl propionate **13** (2.01 g, 15.4 mmol), and 4-methoxybenzaldehyde (2.00 g, 14.7 mmol) in THF (31 mL), and the reaction mixture was stirred for 20 h. The crude product was purified by flash column chromatography (hexane : ethyl acetate = 10 : 1) to provide *tert*-butyl 3-hydroxy-3-(4-methoxyphenyl)-2-methylpropanoate **14q** (1.45 g, 34% yield, dr = 1 : 1).

TLC (hexane : ethyl acetate = 3 : 1): R_f = 0.34; **^{1}H NMR** (500 MHz, $CDCl_3$): δ 7.28–7.26 (m, 2H), 6.89–6.86 (m, 2H), 4.96 (t, *J* = 3.8 Hz, 0.5H), 4.66 (dd, *J* = 4.2, 8.2 Hz, 0.5H), 3.81 (s, 3H), 3.06 (d, *J* = 4.2 Hz, 0.5H), 2.96 (d, *J* = 3.1 Hz, 0.5H), 2.71–2.63 (m, 1H), 1.46 (s, 4.5H), 1.39 (s, 4.5H), 1.12 (d, *J* = 7.3 Hz, 1.5H), 0.99 (d, *J* = 7.3 Hz, 1.5H).

Oxidation of **14q** (1.45 g, 5.44 mmol) was carried out with manganese(IV) oxide (9.46 g, 109 mmol) in dichloromethane (18 mL), and the reaction mixture was stirred for 5 h. The crude product was purified by flash column chromatography (hexane : ethyl acetate = 10 : 1) to provide the title compound as a white solid (773 mg, 54% yield).

mp: 58–59 °C; **TLC** (hexane : ethyl acetate = 10 : 1): R_f = 0.40; **^{1}H NMR** (500 MHz, $CDCl_3$): δ 7.97 (dt, *J* = 8.8, 1.9 Hz, 2H), 6.95 (dt, *J* = 8.8, 1.9 Hz, 2H), 4.22 (q, *J* = 7.3 Hz, 1H), 3.88 (s, 3H), 1.44 (d, *J* = 7.3 Hz, 3H), 1.36 (s, 9H); **^{13}C NMR** (126 MHz, $CDCl_3$): δ 194.6, 170.2, 163.5, 130.8, 129.1, 113.7, 81.5, 55.4, 49.1, 27.7, 13.5; **IR** (neat): 2978, 2943, 1735, 1681, 1604, 1507, 1368, 1310, 1260, 1148, 1029, 959, 840 cm^{-1}; **HRMS** (DART): $[M+H]^+$calcd. for $C_{15}H_{21}O_4$, 265.1440; found, 265.1439.

***tert*-butyl 2-benzyl-3-oxobutanoate (12r)**[6]

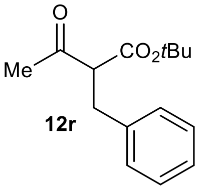

The title compound was prepared following **Method A**, using NaH (792 mg, 33.0 mmol), *tert*-butyl 3-oxobutanoate (4.75 g, 30.0 mmol), and benzyl bromide (6.16 g, 36.0 mmol) in THF (107 mL), and the reaction mixture was stirred for 4 h. The crude product was purified by flash column chromatography (hexane : ethyl acetate = 10 : 1) to provide the title compound as a colourless oil (2.06 g, 28% yield).

Synthesis of β-ketocarboxylic acids 1.

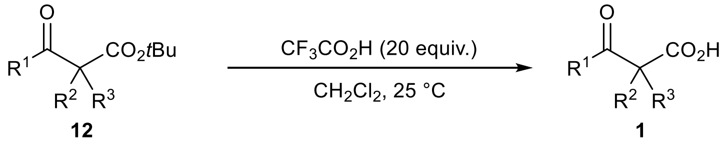

General procedure: To a stirred solution of β-ketoester **12** in dichloromethane was added trifluoroacetic acid (20 equiv.) at 0 °C, and the reaction mixture was stirred at 25 °C. The mixture was concentrated, and then purified by flash column chromatography on silica gel to give β-ketocarboxylic acid **1**.

2-methyl-1-oxo-1,2,3,4-tetrahydronaphthalene-2-carboxylic acid (1a)[11]

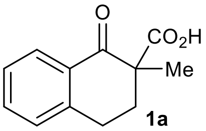

1a

The title compound was prepared following **General procedure**, using **12a** (437 mg, 1.68 mmol) and trifluoroacetic acid (3.83 g, 33.6 mmol) in dichloromethane (8.4 mL), and the reaction mixture was stirred for 30 min. The crude product was purified by flash column chromatography (hexane : diethyl ether = 4 : 1 to 1 : 10) to provide the title compound as a white solid (318 mg, 93% yield) including 2% of a decarboxylated product.

TLC (dichloromethane : methanol = 9 : 1): R_f = 0.41; **^{1}H NMR** (500 MHz, $CDCl_3$): δ 8.06 (dd, *J* = 8.0, 1.2 Hz, 1H), 7.49 (dt, *J* = 7.5, 1.5 Hz, 1H), 7.32 (t, *J* = 7.6 Hz, 1H), 7.23 (d, *J* = 7.6 Hz, 1H), 3.09 (ddd, *J* = 17.4, 8.6, 4.6 Hz, 1H), 3.00–2.94 (m, 1H), 2.57 (ddd, *J* = 13.7, 6.8, 5.0 Hz, 1H), 2.14 (ddd, *J* = 13.4, 8.8, 4.6 Hz, 1H), 1.53 (s, 3H); **^{13}C NMR** (126 MHz, $CDCl_3$): δ 196.5, 178.5, 143.3, 133.9, 131.1, 128.8, 128.2, 126.9, 53.2, 33.2, 25.7, 20.6; **HRMS** (DART): $[M+H]^+$calcd. for $C_{12}H_{13}O_3$, 205.0865; found, 205.0867.

2-allyl-1-oxo-1,2,3,4-tetrahydronaphthalene-2-carboxylic acid (1b)

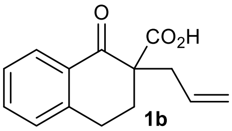

1b

The title compound was prepared following **General procedure**, using **12b** (523 mg, 1.83 mmol) and trifluoroacetic acid (4.17 g, 36.5 mmol) in dichloromethane (9.0 mL), and the reaction mixture was stirred for 30 min. The crude product was purified by flash column chromatography (hexane : diethyl ehter = 4 : 1 to 1 : 1) to provide the title compound as a colourless oil (336 mg, 80% yield) including 4% of a decarboxylated product. Carboxylic acid

was crystallized at −20 °C, filtered, and washed with hexane.

TLC (dichloromethane : methanol = 9 : 1): R_f = 0.45; **^{1}H NMR** (500 MHz, $CDCl_3$): δ 8.09 (dd, *J* = 8.0, 1.2 Hz, 1H), 7.54 (dt, *J* = 7.6, 1.5 Hz, 1H), 7.35 (t, *J* = 7.3 Hz, 1H), 7.26 (d, *J* = 7.6 Hz, 1H), 5.79 (ddt, *J* = 17.2, 10.0, 7.2 Hz, 1H), 5.17–5.12 (m, 2H), 3.05 (t, *J* = 6.1 Hz, 2H), 2.70 (d, *J* = 7.6 Hz, 2H), 2.48–2.36 (m, 2H); **^{13}C NMR** (126 MHz, $CDCl_3$): δ 195.9, 176.6, 143.4, 134.0, 132.5, 131.2, 128.8, 128.2, 126.9, 119.5, 56.7, 38.4, 29.7, 25.3; **HRMS** (DART): $[M+H]^+$ calcd. for $C_{14}H_{15}O_3$, 231.1021; found, 231.1020.

2-benzyl-1-oxo-1,2,3,4-tetrahydronaphthalene-2-carboxylic acid (1c)

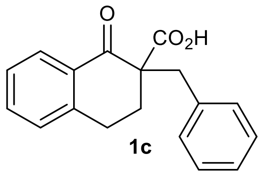

The title compound was prepared following **General procedure**, using **12c** (189 mg, 0.562 mmol) and trifluoroacetic acid (1.28 g, 11.2 mmol) in dichloromethane (2.8 mL), and the reaction mixture was stirred for 40 min. The crude product was purified by flash column chromatography (hexane : diethyl ether = 4 : 1 to 1 : 1) to provide the title compound as a colourless oil (129 mg, 82% yield) including 9% of a decarboxylated product.

TLC (dichloromethane : methanol = 9 : 1): R_f = 0.34; **^{1}H NMR** (500 MHz, $CDCl_3$): δ 8.10 (dd, *J* = 7.6, 1.2 Hz, 1H), 7.54 (dt, *J* = 7.5, 1.2 Hz, 1H), 7.36 (t, *J* = 7.3 Hz, 1H), 7.27–7.21 (m, 5H), 7.11 (d, *J* = 7.3 Hz, 1H), 3.36 (d, *J* = 13.8 Hz, 1H), 3.26 (d, *J* = 13.8 Hz, 1H), 3.15–3.05 (m, 2H), 2.45–2.31 (m, 2H); **^{13}C NMR** (126 MHz, $CDCl_3$): δ 195.8, 176.6, 143.5, 135.7, 134.2, 131.7, 130.6, 128.9, 128.4, 128.3, 127.1, 127.0, 58.1, 40.2, 29.9, 25.7; **HRMS** (DART): $[M+NH_4]^+$ calcd. for $C_{18}H_{20}N_1O_3$, 298.1443; found, 298.1445.

2-(3-chloropropyl)-1-oxo-1,2,3,4-tetrahydronaphthalene-2-carboxylic acid (1d)

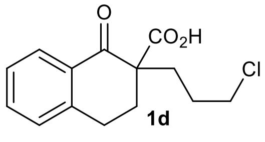

The title compound was prepared following **General procedure**, using **12d** (160 mg, 0.497 mmol) and trifluoroacetic acid (1.13 g, 9.94 mmol) in dichloromethane (2.5 mL), and the reaction mixture was stirred for 20 min. The crude product was purified by flash column chromatography (hexane : diethyl ether = 4 : 1 to 1 : 2) to provide the title compound as a white solid (115 mg, 87% yield) including 3% of a decarboxylated product.

TLC (dichloromethane : methanol = 9 : 1): R_f = 0.45; **^{1}H NMR** (500 MHz, $CDCl_3$): δ 9.62 (bs,

1H), 8.05 (d, J = 7.6 Hz, 1H), 7.50 (t, J = 7.3 Hz, 1H), 7.32 (t, J = 7.6 Hz, 1H), 7.25 (d, J = 7.6 Hz, 1H), 3.59–3.50 (m, 2H), 3.11–2.98 (m, 2H), 2.55–2.50 (m, 1H), 2.30–2.24 (m, 1H), 2.08–2.05 (m, 2H), 1.98–1.81 (m, 2H); **^{13}C NMR** (126 MHz, $CDCl_3$): δ 196.4, 176.5, 143.2, 134.2, 131.1, 128.8, 128.3, 127.0, 56.3, 44.8, 31.3, 29.9, 27.8, 25.4; **HRMS** (DART): $[M+H]^+$calcd. for $C_{14}H_{16}Cl_1O_3$, 267.0788; found, 267.0787.

2-(2-cyanoethyl)-1-oxo-1,2,3,4-tetrahydronaphthalene-2-carboxylic acid (1e)

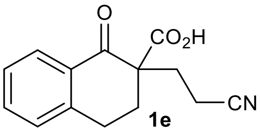

The title compound was prepared following **General procedure**, using **12e** (165 mg, 0.551 mmol) and trifluoroacetic acid (1.25 g, 11.0 mmol) in dichloromethane (2.8 mL), and the reaction mixture was stirred for 1.5 h. The crude product was purified by flash column chromatography (hexane : diethyl ether = 4 : 1 to 1 : 5) to provide the title compound as a pale yellow oil (98.6 mg, 73% yield) including 9% of a decarboxylated product.

TLC (dichloromethane : methanol = 9 : 1): R_f = 0.16; **^{1}H NMR** (500 MHz, $CDCl_3$): δ 8.06 (dd, J = 7.8, 2.5 Hz, 1H), 7.56–7.53 (m, 1H), 7.36 (t, J = 7.5 Hz, 1H), 7.27 (d, J = 6.9 Hz, 1H), 3.17–3.00 (m, 2H), 2.68–2.55 (m, 3H), 2.36–2.24 (m, 3H); **^{13}C NMR** (126 MHz, $CDCl_3$): δ 194.9, 174.5, 142.7, 134.1, 131.4, 128.8, 128.1, 127.0, 119.3, 56.1, 31.3, 29.9, 25.6, 13.3; **HRMS** (DART): $[M+NH_4]^+$calcd. for $C_{14}H_{17}N_2O_3$, 261.1239; found, 261.1236.

6-chloro-3-methyl-4-oxochromane-3-carboxylic acid (1f)

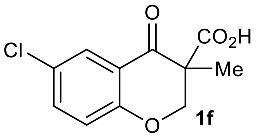

The title compound was prepared following **General procedure**, using **12f** (116 mg, 0.391 mmol) and trifluoroacetic acid (891 mg, 7.81 mmol) in dichloromethane (2.0 mL), and the reaction mixture was stirred for 2 h. The crude product was purified by flash column chromatography (hexane : diethyl ether = 4 : 1 to 1 : 5) to provide the title compound as a white solid (75.9 mg, 84% yield).

TLC (dichloromethane : methanol = 9 : 1): R_f = 0.20; **^{1}H NMR** (500 MHz, Acetone-d_6): δ 7.78 (d, J = 2.7 Hz, 1H), 7.55 (d, J = 8.8 Hz, 1H), 7.06 (d, J = 9.2 Hz, 1H), 4.82 (d, J = 11.9 Hz, 1H), 4.41 (d, J = 11.9 Hz, 1H), 1.41 (s, 3H); **^{13}C NMR** (126 MHz, Acetone-d_6): δ 190.0, 171.9, 160.8, 136.3, 127.3, 127.0, 122.2, 120.8, 74.8, 53.6, 15.8; **HRMS** (DART): $[M+H]^+$calcd. for $C_{11}H_{10}Cl_1O_4$, 241.0268; found, 241.0270.

2-methyl-1-oxo-2,3-dihydro-1*H*-indene-2-carboxylic acid (1g)

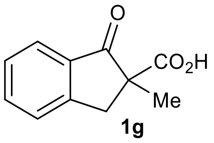

1g

The title compound was prepared following **General procedure**, using **12g** (190 mg, 0.771 mmol) and trifluoroacetic acid (1.76 g, 15.4 mmol) in dichloromethane (3.9 mL), and the reaction mixture was stirred for 30 min. The crude product was purified by flash column chromatography (hexane : diethyl ether = 4 : 1 to 1 : 2) to provide the title compound as a white solid (120 mg, 82% yield) including 9% of a decarboxylated product

TLC (dichloromethane : methanol = 9 : 1): R_f = 0.41; **^{1}H NMR** (500 MHz, $CDCl_3$): δ 7.79 (d, *J* = 8.0 Hz, 1H), 7.64 (dt, *J* = 7.5, 1.2 Hz, 1H), 7.49–7.47 (m, 1H), 7.41 (dt, *J* = 7.6, 0.8 Hz, 1H), 3.77 (d, *J* = 17.2 Hz, 1H), 3.03 (d, *J* = 17.2 Hz, 1H), 1.52 (s, 3H); **^{13}C NMR** (126 MHz, $CDCl_3$): δ 203.5, 177.3, 152.7, 135.8, 134.4, 128.1, 126.6, 125.2, 56.1, 40.0, 21.4; **HRMS** (DART): $[M+NH_4]^+$calcd. for $C_{11}H_{14}N_1O_3$, 208.0974; found, 208.0973.

5-bromo-2-methyl-1-oxo-2,3-dihydro-1*H*-indene-2-carboxylic acid (1h)

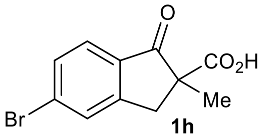

1h

The title compound was prepared following **General procedure**, using **12h** (149 mg, 0.458 mmol) and trifluoroacetic acid (1.04 g, 9.16 mmol) in dichloromethane (2.3 mL), and the reaction mixture was stirred for 30 min. The crude product was purified by flash column chromatography (hexane : diethyl ether = 4 : 1 to 1: 1) to provide the title compound as a white solid (103 mg, 84% yield) including 7% of a decarboxylated product.

TLC (dichloromethane : methanol = 9 : 1): R_f = 0.36; **^{1}H NMR** (500 MHz, Acetone-d_6): δ 7.82 (t, *J* = 1.2 Hz, 1H), 7.64 (d, *J* = 0.8 Hz, 2H), 3.73 (dd, *J* = 17.6, 0.8 Hz, 1H), 3.11 (dd, *J* = 17.8, 0.8 Hz, 1H), 1.45 (s, 3H); **^{13}C NMR** (126 MHz, Acetone-d_6): δ 202.9, 172.8, 155.9, 134.8, 132.1, 130.8, 130.7, 126.5, 56.6, 40.2, 20.7; **HRMS** (DART): $[M+NH_4]^+$ calcd. for $C_{11}H_{13}Br_1N_1O_3$, 286.0079; found, 286.0077.

2-methyl-3-oxo-2,3-diphenylpropanoic acid (1i)

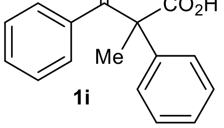

The title compound was prepared following **General procedure**, using **12i** (85.8 mg, 0.276 mmol) and trifluoroacetic acid (630 mg, 5.53 mmol) in dichloromethane (1.4 mL), and the reaction mixture was stirred for 30 min. The crude product was purified by flash column chromatography (hexane : diethyl ether = 4 : 1 to 1 : 1) to provide the title compound as a white solid (66.9 mg, 95% yield) including 1% of a decarboxylated product.

TLC (dichloromethane : methanol = 5 : 1): R_f = 0.40; ^{1}**H NMR** (500 MHz, $CDCl_3$): δ 7.63–7.62 (m, 2H), 7.44–7.40 (m, 3H), 7.35–7.24 (m, 5H), 1.97 (s, 3H); ^{13}**C NMR** (126 MHz, $CDCl_3$): δ 197.8, 177.6, 138.5, 134.9, 132.7, 129.8, 128.6, 128.2, 127.8, 127.6, 62.7, 24.1; **HRMS** (DART): $[M+H]^+$calcd. for $C_{16}H_{15}O_3$, 255.1021; found, 255.1023.

1-benzyl-2-oxocyclohexane-1-carboxylic acid (1j)

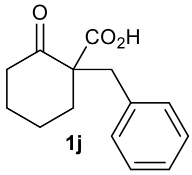

The title compound was prepared following **General procedure**, using **12j** (750 mg, 2.60 mmol) and trifluoroacetic acid (5.93 g, 52.0 mmol) in dichloromethane (13 mL), and the reaction mixture was stirred for 20 min. The crude product was purified by flash column chromatography (hexane : diethyl ether = 4 : 1 to 1 : 1) to provide the title compound as a white solid (444 mg, 74% yield) including 2% of a decarboxylated product.

TLC (dichloromethane : methanol = 9 : 1): R_f = 0.60; ^{1}**H NMR** (500 MHz, $CDCl_3$): δ 7.28–7.23 (m, 3H), 7.14 (dd, J = 7.3, 1.2 Hz, 2 H), 3.34 (d, J = 14.1 Hz, 1H), 3.06 (d, J = 13.8 Hz, 1H), 2.61–2.48 (m, 2H), 2.31–2.27 (m, 1H), 2.02–1.96 (m, 1H), 1.85–1.68 (m, 4H); ^{13}**C NMR** (126 MHz, $CDCl_3$): δ 207.6, 176.9, 135.9, 130.2, 128.1, 126.9, 61.8, 41.0, 40.3, 35.3, 27.3, 22.2; **HRMS** (DART): $[M+H]^+$calcd. for $C_{14}H_{17}O_3$, 233.1178; found, 233.1177.

2-methyl-3-oxo-3-phenylpropanoic acid (1k)[12]

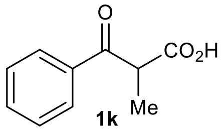

The title compound was prepared following **General procedure**, using **12k** (437 mg, 1.86 mmol) and trifluoroacetic acid (4.25 g, 37.3 mmol) in dichloromethane (9.3 mL), and the reaction mixture was stirred for 1 h. The crude product was purified by flash column chromatography (hexane : diethyl ether = 4 : 1 to 1 : 1) to provide the title compound as a white solid (276 mg, 83% yield).

TLC (dichloromethane : methanol = 10 : 1): R_f = 0.32; **^{1}H NMR** (500 MHz, $CDCl_3$): δ 8.00 (d, *J* = 7.3 Hz, 2H), 7.63 (t, *J* = 7.3 Hz, 1H), 7.51 (t, *J* = 7.3 Hz, 2H), 4.46 (q, *J* = 7.3 Hz, 1H), 1.56 (d, *J* = 7.3 Hz, 3H); **^{13}C NMR** (126 MHz, $CDCl_3$): δ 195.9, 176.4, 135.4, 133.8, 128.8, 128.7, 47.6, 14.1; **HRMS** (DART): $[M+H]^+$calcd. for $C_{10}H_{11}O_3$, 179.0708; found, 179.0708.

2-benzoylpent-4-enoic acid (1l)

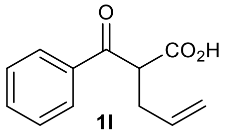

1l

The title compound was prepared following **General procedure**, using **12l** (91.1 mg, 0.350 mmol) and trifluoroacetic acid (798 mg, 7.00 mmol) in dichloromethane (1.8 mL), and the reaction mixture was stirred for 30 min. The crude product was purified by flash column chromatography (hexane : diethyl ether = 4 : 1 to 1 : 1) to provide the title compound as a pale yellow oil (67.6 mg, 94% yield).

TLC (dichloromethane : methanol = 10 : 1): R_f = 0.19; **^{1}H NMR** (500 MHz, $CDCl_3$): δ 9.96 (s, 1H), 7.99 (d, *J* = 7.6 Hz, 2H), 7.59 (t, *J* = 7.6 Hz, 1H), 7.48 (t, *J* = 7.6 Hz, 2H), 5.79 (ddt, *J* = 17.0, 10.3, 6.9 Hz, 1H), 5.11 (dd, *J* = 17.0, 1.2 Hz, 1H), 5.04 (d, *J* = 10.3 Hz, 1H), 4.47 (t, *J* = 6.9 Hz, 1H), 2.75 (t, *J* = 6.9 Hz, 2H); **^{13}C NMR** (126 MHz, $CDCl_3$): δ 194.5, 174.9, 135.8, 133.8, 128.8, 128.7, 117.8, 53.1, 33.1; **HRMS** (DART): $[M+H]^+$calcd. for $C_{12}H_{13}O_3$, 205.0865; found, 205.0865.

2-benzyl-3-oxo-3-phenylpropanoic acid (1m)

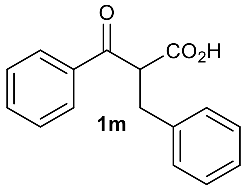

1m

The title compound was prepared following **General procedure**, using **12m** (160 mg, 0.515 mmol) and trifluoroacetic acid (1.18 g, 10.3 mmol) in dichloromethane (2.6 mL), and the reaction mixture was stirred for 30 min. The crude product was purified by flash column chromatography (hexane : diethyl ether = 4 : 1 to 1 : 1) to provide the title compound as a white solid (121 mg, 92% yield).

TLC (dichloromethane : methanol = 10 : 1): R_f = 0.21; **^{1}H NMR** (500 MHz, $CDCl_3$): δ 8.83 (s, 1H), 7.93 (d, *J* = 7.6 Hz, 2H), 7.56 (t, *J* = 7.6 Hz, 1H), 7.44 (t, *J* = 7.6 Hz, 2H), 7.26–7.16 (m, 5H), 4.69 (t, *J* = 7.3 Hz, 1H), 3.32 (d, *J* = 7.3 Hz, 2H); **^{13}C NMR** (126 MHz, $CDCl_3$): δ 194.6, 174.5, 137.8, 135.8, 133.8, 128.8, 128.8, 128.7, 128.6, 126.8, 55.3, 35.0; **HRMS** (DART): [M

+H]$^+$calcd. for $C_{16}H_{15}O_3$, 255.1021; found, 255.1019.

2-methyl-3-oxo-3-(*p*-tolyl)propanoic acid (1n)

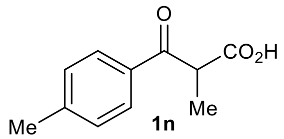

The title compound was prepared following **General procedure**, using **12n** (621 mg, 2.50 mmol) and trifluoroacetic acid (5.7 g, 50.0 mmol) in dichloromethane (13 mL), and the reaction mixture was stirred for 30 min. The crude product was purified by flash column chromatography (hexane : diethyl ether = 3 : 1 to 1 : 1) to provide the title compound as a white solid (462 mg, 96% yield) including 2% of a decarboxylated product.

TLC (dichloromethane : methanol = 10 : 1): R_f = 0.24; **^{1}H NMR** (500 MHz, $CDCl_3$): δ 7.88 (d, *J* = 7.6 Hz, 2H), 7.27 (d, *J* = 7.6 Hz, 2H), 4.42 (q, *J* = 6.9 Hz, 1H), 2.41 (s, 3H), 1.49 (d, *J* = 6.9 Hz, 3H); **^{13}C NMR** (126 MHz, $CDCl_3$): δ 195.5, 176.6, 144.8, 132.8, 129.5, 128.8, 47.4, 21.6, 14.1; **HRMS** (DART): [M+H]$^+$calcd. for $C_{11}H_{13}O_3$, 193.0865; found, 193.0864.

3-(4-fluorophenyl)-2-methyl-3-oxopropanoic acid (1o)

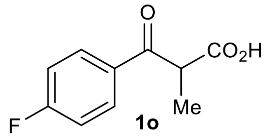

The title compound was prepared following **General procedure**, using **12o** (654 mg, 2.59 mmol) and trifluoroacetic acid (5.91 g, 51.8 mmol) in dichloromethane (13 mL), and the reaction mixture was stirred for 30 min. The crude product was purified by flash column chromatography (hexane : diethyl ether = 3 : 1 to 1 : 1) to provide the title compound as a white solid (474 mg, 93% yield).

TLC (dichloromethane : methanol = 10 : 1): R_f = 0.24; **^{1}H NMR** (500 MHz, $CDCl_3$): δ 8.03 (dd, *J* = 8.8, 5.4 Hz, 2H), 7.17 (d, *J* = 8.8 Hz, 2H), 4.41 (q, *J* = 7.3 Hz, 1H), 1.53 (d, *J* = 7.3 Hz, 3H); **^{13}C NMR** (126 MHz, $CDCl_3$): δ 194.1, 176.5, 166.1 (d, *J* = 256.7 Hz), 131.8 (d, *J* = 2.4 Hz), 131.4 (d, *J* = 9.6 Hz), 116.0 (d, *J* = 21.6 Hz), 47.6, 13.8; **^{19}F NMR** (376 MHz, $CDCl_3$): δ –103.9; **HRMS** (DART): [M+H]$^+$calcd. for $C_{10}H_{10}F_1O_3$, 194.0614; found, 194.0614.

3-(3-chlorophenyl)-2-methyl-3-oxopropanoic acid (1p)

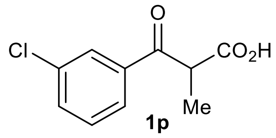

The title compound was prepared following **General procedure**, using **12p** (646 mg, 2.40 mmol) and trifluoroacetic acid (5.48 g, 48.0 mmol) in dichloromethane (12 mL), and the reaction mixture was stirred for 30 min. The crude product was purified by flash column chromatography (hexane : diethyl ether = 3 : 1 to 1 : 1) to provide the title compound as a white solid (460 mg, 90% yield).

TLC (dichloromethane : methanol = 10 : 1): R_f = 0.19; **^{1}H NMR** (500 MHz, $CDCl_3$): δ 7.95 (t, *J* = 1.9 Hz, 1H), 7.85 (dt, *J* = 8.0, 1.2 Hz, 1H), 7.57 (ddd, *J* = 8.0, 1.9, 1.2 Hz, 1H), 7.43 (t, *J* = 8.0 Hz, 1H), 4.41 (q, *J* = 6.9 Hz, 1H), 1.51 (d, *J* = 6.9 Hz, 3H); **^{13}C NMR** (126 MHz, $CDCl_3$): δ 194.4, 176.3, 137.0, 135.2, 133.7, 130.1, 128.7, 126.7, 47.7, 13.7; **HRMS** (DART): $[M+H]^+$ calcd. for $C_{10}H_{10}Cl_1O_3$, 213.0319; found, 213.0319.

3-(4-methoxyphenyl)-2-methyl-3-oxopropanoic acid (1q)

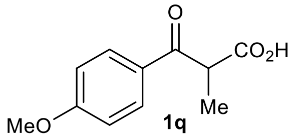

The title compound was prepared following **General procedure**, using **12q** (222 mg, 0.838 mmol) and trifluoroacetic acid (1.91 g, 16.8 mmol) in dichloromethane (4.2 mL), and the reaction mixture was stirred for 30 min. The crude product was purified by flash column chromatography (hexane : diethyl ether = 3 : 1 to 1 : 1) to provide the title compound as a white solid (160 mg, 92% yield) including 2% of a decarboxylated product.

TLC (dichloromethane : methanol = 10 : 1): R_f = 0.18; **^{1}H NMR** (500 MHz, $CDCl_3$): δ 7.98 (d, *J* = 8.8 Hz, 2H), 6.96 (d, *J* = 8.8 Hz, 2H), 4.41 (q, *J* = 7.3 Hz, 1H), 3.88 (s, 3H), 1.51 (d, *J* = 7.3 Hz, 3H); **^{13}C NMR** (126 MHz, $CDCl_3$): δ 194.8, 176.1, 164.2, 131.2, 128.2, 114.0, 55.5, 47.1, 14.6; **HRMS** (DART): $[M+H]^+$calcd. for $C_{11}H_{13}O_4$, 209.0814; found, 209.0815.

2-benzyl-3-oxobutanoic acid (1r)[13]

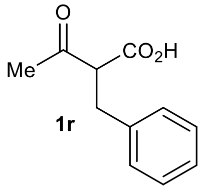

The title compound was prepared following **General procedure**, using **12r** (265 mg, 1.07 mmol) and trifluoroacetic acid (2.42 g, 21.3 mmol) in dichloromethane (5.3 mL), and the reaction mixture was stirred for 30 min. The crude product was purified by flash column chromatography (hexane : diethyl ether = 4 : 1 to 1 : 1) to provide the title compound as a white solid (162 mg, 79% yield) including 2% of a decarboxylated product.

TLC (dichloromethane : methanol = 10 : 1): R_f = 0.19; **^{1}H NMR** (500 MHz, $CDCl_3$): [keto form]: δ 7.30–7.18 (m, 5H), 3.85 (t, *J* = 7.6 Hz, 1H), 3.18 (dd, *J* = 14.1, 7.6 Hz, 2H), 2.21 (s, 3H); [enol form]: δ 12.58 (s, 1H), 7.30–7.18 (m, 5H), 3.6 (s, 2H), 2.08 (s, 3H); **^{13}C NMR** (126 MHz, $CDCl_3$): [keto and enol form] δ 202.4, 177.4, 177.1, 174.6, 140.2, 137.6, 128.7, 128.7, 128.4, 127.7, 126.8, 126.0, 98.6, 60.7, 33.9, 31.6, 30.0, 19.5; **HRMS** (DART): $[M+H]^+$calcd. for $C_{11}H_{13}O_3$, 193.0865; found, 193.0865.

1-oxo-1,2,3,4-tetrahydronaphthalene-2-carboxylic acid (1s)

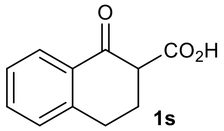

The title compound was prepared following **General procedure**, using **12s** (266 mg, 1.08 mmol) and trifluoroacetic acid (2.46 g, 21.6 mmol) in dichloromethane (5.4 mL), and the reaction mixture was stirred for 40 min. The crude product was purified by flash column chromatography (hexane : diethyl ether = 4 : 1 to 1 : 1) to provide the title compound as a white solid (134 mg, 63% yield) including 2% of a decarboxylated product.

TLC (dichloromethane : methanol = 9 : 1): R_f = 0.30; **^{1}H NMR** (500 MHz, $CDCl_3$): [keto form]: δ 8.11 (d, *J* = 7.6 Hz, 1H), 7.56 (t, *J* = 7.6 Hz, 1H), 7.36 (t, *J* = 7.6 Hz, 1H), 7.30 (t, *J* = 7.6 Hz, 1H), 3.54 (dd, *J* = 11.9, 4.6 Hz, 1H), 3.14–3.03 (m, 2H), 2.69–2.65 (m, 1H), 2.41–2.33 (m, 1H); [enol form]: δ 12.19 (s, 1H), 7.83 (d, *J* = 7.6 Hz, 1H), 7.36 (t, *J* = 7.6 Hz, 1H), 7.30 (t, *J* = 7.6 Hz, 1H), 7.20 (d, *J* = 7.6 Hz, 1H), 2.85 (t, *J* = 7.8 Hz, 2H), 2.63 (t, *J* = 7.8 Hz, 2H); **^{13}C NMR** (126 MHz, $CDCl_3$): [keto and enol form]: δ 194.4, 177.2, 174.5, 167.4, 144.1, 140.0, 134.5, 131.3, 131.1, 129.6, 128.9, 127.9, 127.5, 127.0, 126.6, 124.7, 96.1, 53.1, 27.8, 27.6, 26.0, 20.5; **HRMS** (DART): $[M+H]^+$calcd. for $C_{11}H_{11}O_3$, 191.0708; found, 191.0708.

General procedure for enantioselective decarboxylative chlorination (Fig. 3).

Enantioselective decarboxylative chlorination of **1** was performed by either **Method A** or **Method B** described below. When the reactions were performed, some starting compounds **1** contained 1–9% of decarboxylated by-product as an impurity, as noted in the description of the synthesis of each compound, because some of **1** decomposed slowly while standing at ambient temperature.

[Method A]: Chlorination of α,α-dialkyl-β-ketocarboxylic acids 1a-1j

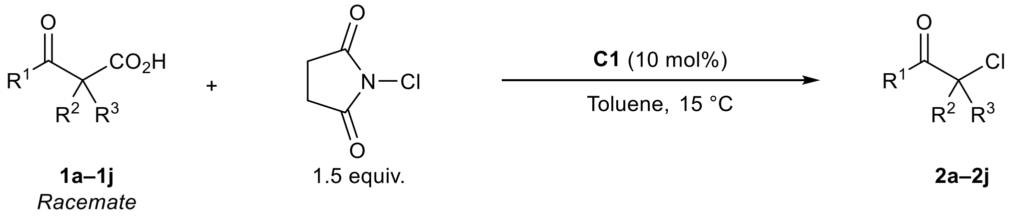

To a stirred solution of amine catalyst **C1** (10 mol%) and *N*-chlorosuccinimide (1.5 equiv.) in toluene was added α,α-dialkyl-β-ketocarboxylic acid **1**. Then, the reaction mixture was stirred in the dark. The mixture was purified by flash column chromatography on silica gel to give α-chloroketone **2**.

[Method B]: Chlorination of α-monoalkyl-β-ketocarboxylic acids 1k-1s

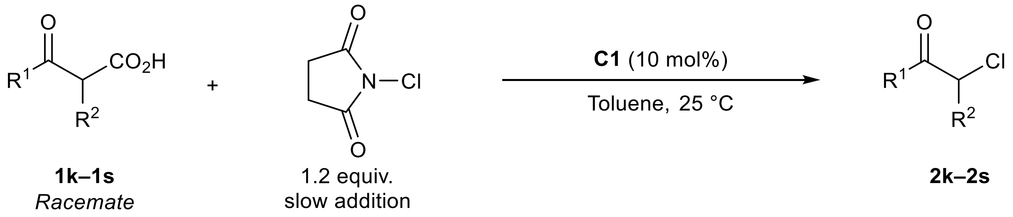

To a stirred solution of amine catalyst **C1** (10 mol%), *N*-chlorosuccinimide (0.1 equiv.) and α-alkyl-β-ketocarboxylic acid **1** in toluene was added slowly a solution of *N*-chlorosuccinimide (1.1 equiv.) in toluene over 1 h (0.5 h for the synthesis of **2r**) using a syringe pump in the dark. Then, the reaction mixture was stirred at 25 °C for another 5 min (2 h for the synthesis of **2s**). The mixture was purified by flash column chromatography on silica gel to give α-chloroketone **2**.

(*S*)-2-chloro-2-methyl-3,4-dihydronaphthalen-1(2*H*)-one (2a)[14]

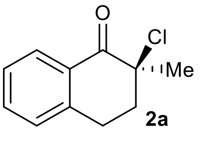

The title compound was prepared following **Method A**, using **C1** (18.6 mg, 0.0245 mmol), *N*-chlorosuccinimide (49.1 mg, 0.368 mmol), and **1a** (50.1 mg, 0.245 mmol) in toluene (1.2 mL), and the reaction mixture was stirred for 24 h. The crude product was purified by flash column chromatography (hexane : dichloromethane = 2 : 1 to 1 : 2) to provide the title compound as a white solid (44.9 mg, 94% yield, 96% e.e.).

mp: 32 °C; **TLC** (hexane : dichloromethane = 2 : 1): R_f = 0.34; $[\alpha]_D^{25}$ = +51.6 (*c* 1.4, $CHCl_3$); **^{1}H NMR** (500 MHz, $CDCl_3$): δ 8.11 (dd, *J* = 7.8, 1.2 Hz, 1H), 7.51 (dt, *J* = 7.5, 1.2 Hz, 1H),

7.34 (t, J = 7.6 Hz, 1H), 7.25 (d, J = 7.6 Hz, 1H), 3.39 (ddd, J = 17.2, 11.3, 4.8 Hz, 1H), 2.89 (ddd, J = 17.2, 4.4, 3.4 Hz, 1H), 2.50 (ddd, J = 14.5, 4.6, 3.1 Hz, 1H), 2.34 (ddd, J = 14.5, 11.3, 4.8 Hz, 1H), 1.83 (s, 3H); **^{13}C NMR** (126 MHz, $CDCl_3$): δ 191.5, 143.2, 133.9, 129.9, 129.1, 128.8, 127.1, 67.7, 38.6, 26.8, 26.1; **IR** (neat): 2932, 1695, 1601, 1458, 1307, 1234, 1074, 905, 804, 740, 606, 481 cm^{-1}; **HRMS** (DART): $[M+H]^+$calcd. for $C_{11}H_{12}Cl_1O_1$, 195.0577; found, 195.0575.

The enantiomeric purity of the title compound was determined by HPLC analyses (DAICEL CHIRALCEL OD-H (0.46 cmϕ × 25 cm), hexane : 2-propanol = 500 : 1, flow rate = 1.0 mL/min, retention time; 8.9 min (major) and 10.0 min (minor)).

The absolute configuration of **2a** was determined to be *S* by X-ray crystallographic analysis (see Supplementary Table 3).

2-allyl-2-chloro-3,4-dihydronaphthalen-1(2*H*)-one (2b)[15]

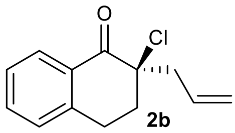

The title compound was prepared following **Method A**, using **C1** (9.3 mg, 0.012 mmol), *N*-chlorosuccinimide (24.6 mg, 0.184 mmol), and **1b** (28.3 mg, 0.123 mmol) in toluene (0.62 mL), and the reaction mixture was stirred for 18 h. The crude product was purified by flash column chromatography (hexane : dichloromethane = 4 : 1 to 1 : 2) to provide the title compound as a colourless oil (26.1 mg, 96% yield, 96% e.e.).

TLC (hexane : ethyl acetate = 9 : 1): R_f = 0.45; $[\alpha]_D^{24}$ = +50.5 (*c* 0.7, $CHCl_3$); **^{1}H NMR** (500 MHz, $CDCl_3$): δ 8.12 (dd, J = 8.0, 1.2 Hz, 1H), 7.51 (dt, J = 7.5, 1.2 Hz, 1H), 7.35 (t, J = 7.6 Hz, 1H), 7.25 (d, J = 6.9 Hz, 1H), 5.88 (ddt, J = 16.8, 10.4, 7.2 Hz, 1H), 5.23–5.21 (m, 1H), 5.19 (t, J = 1.2 Hz, 1H), 3.34 (ddd, J = 18.1, 11.1, 5.0 Hz, 1H), 2.98 (ddt, J = 14.1, 6.9, 1.2 Hz, 1H), 2.93–2.86 (m, 2H), 2.43 (ddd, J = 14.5, 4.6, 3.4 Hz, 1H), 2.32 (ddd, J = 14.8, 11.0, 4.7 Hz, 1H); **^{13}C NMR** (126 MHz, $CDCl_3$): δ 190.9, 143.2, 134.0, 132.4, 130.1, 129.1, 128.8, 127.1, 120.0, 69.9, 43.0, 35.2, 25.9; **IR** (neat): 3075, 2953, 2935, 1690, 1599, 1454, 1429, 1287, 1241, 1225, 922, 745 cm^{-1}; **HRMS** (DART): $[M+NH_4]^+$calcd. for $C_{13}H_{17}Cl_1N_1O_1$, 238.0999; found, 238.0999.

The enantiomeric purity of the title compound was determined by HPLC analyses (DAICEL CHIRALCEL OD-H (0.46 cmϕ × 25 cm), hexane : 2-propanol = 500 : 1, flow rate = 1.0 mL/min, retention time; 8.7 min (major) and 10.5 min (minor)).

2-benzyl-2-chloro-3,4-dihydronaphthalen-1(2*H*)-one (2c)

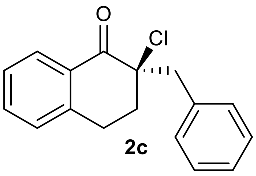

The title compound was prepared following **Method A**, using **C1** (7.8 mg, 0.010 mmol), and *N*-chlorosuccinimide (20.6 mg, 0.155 mmol), and **1c** (28.8 mg, 0.103 mmol) in toluene (1.0 mL), and the reaction mixture was stirred for 6 h. The crude product was purified by flash column chromatography (hexane : dichloromethane = 2 : 1 to 1 : 2) to provide the title compound as a as a colourless oil (25.2 mg, 91% yield, 93% e.e.).

TLC (hexane : ethyl acetate = 9 : 1): R_f = 0.48; $[\alpha]_D^{27}$ = −22.7 (*c* 1.0, $CHCl_3$); **^{1}H NMR** (400 MHz, $CDCl_3$): δ 8.15 (dd, *J* = 7.9, 0.9 Hz, 1H), 7.49 (dt, *J* = 7.5, 1.2 Hz, 1H), 7.34 (t, *J* = 7.6 Hz, 1H), 7.31–7.23 (m, 5H), 7.21 (d, *J* = 7.9 Hz, 1H), 3.60 (d, *J* = 13.7 Hz, 1H), 3.47 (d, *J* = 14.0 Hz, 1H), 3.30 (ddd, *J* = 16.6, 11.3, 4.9 Hz, 1H), 2.83 (dt, *J* = 17.1, 3.7 Hz, 1H), 2.31 (ddd, *J* = 14.7, 4.9, 3.1 Hz, 1H), 2.23 (ddd, *J* = 14.7, 11.3, 4.6 Hz, 1H); **^{13}C NMR** (100 MHz, $CDCl_3$): δ 190.6, 143.0, 135.5, 133.8, 131.2, 130.0, 129.0, 128.6, 128.1, 127.0, 127.0, 70.4, 44.1, 34.7, 25.6; **IR** (neat): 3063, 3031, 2933, 1690, 1603, 1495, 1453, 1436, 1295, 1238, 739, 703, 584, 534 cm^{-1}; **HRMS** (DART): $[M+NH_4]^+$calcd. for $C_{17}H_{19}Cl_1N_1O_1$, 288.1155; found, 288.1153.

The enantiomeric purity of the title compound was determined by HPLC analyses (DAICEL CHIRALCEL OD-H (0.46 cmϕ × 25 cm), hexane : 2-propanol = 500 : 1, flow rate = 1.0 mL/min, retention time; 13.5 min (major) and 17.9 min (minor)).

2-chloro-2-(3-chloropropyl)-3,4-dihydronaphthalen-1(2*H*)-one (2d)

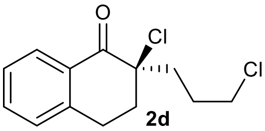

The title compound was prepared following **Method A**, using **C1** (8.4 mg, 0.011 mmol), *N*-chlorosuccinimide (22.2 mg, 0.166 mmol), and **1d** (29.6 mg, 0.111 mmol) in toluene (0.56 mL), and the reaction mixture was stirred for 8 h. The crude product was purified by flash column chromatography (hexane : dichloromethane = 2 : 1 to 1 : 1) to provide the title compound as a colourless oil (25.6 mg, 90% yield, 97% e.e.).

TLC (hexane : ethyl acetate = 9 : 1): R_f = 0.38; $[\alpha]_D^{28}$ = +29.4 (*c* 1.2, $CHCl_3$); **^{1}H NMR** (500 MHz, $CDCl_3$): δ 8.11 (dd, *J* = 8.0, 1.2 Hz, 1H), 7.52 (dt, *J* = 7.5, 1.5 Hz, 1H), 7.36 (t, *J* = 7.6 Hz, 1H), 7.26 (d, *J* = 7.6 Hz, 1H), 3.67–3.58 (m, 2H), 3.36 (ddd, *J* = 16.3, 10.7, 4.6 Hz, 1H), 2.94 (dt, *J* = 17.2, 4.2 Hz, 1H), 2.49 (ddd, *J* = 14.5, 4.6, 3.8 Hz, 1H), 2.41–2.30 (m, 2H), 2.22 (ddd, *J*

= 14.0, 11.7, 4.2 Hz, 1H), 2.13–2.04 (m, 1H), 1.97–1.89 (m, 1H); **^{13}C NMR** (126 MHz, $CDCl_3$): δ 190.9, 142.7, 133.9, 129.9, 129.0, 128.7, 127.1, 70.6, 44.8, 35.9, 35.4, 27.6, 25.8; **IR** (neat): 2957, 2931, 2851, 1690, 1604, 1459, 1294, 1238, 918, 819, 743 cm^{-1}; **HRMS** (DART): $[M+H]^+$calcd. for $C_{13}H_{15}Cl_2O_1$, 257.0500; found, 257.0503.

The enantiomeric purity of the title compound was determined by HPLC analyses (DAICEL CHIRALPAK IB-3 (0.46 cmϕ × 25 cm), hexane : 2-propanol = 100 : 1, flow rate = 1.0 mL/min, retention time; 8.3 min (major) and 8.8 min (minor)).

3-(2-chloro-1-oxo-1,2,3,4-tetrahydronaphthalen-2-yl)propanenitrile (2e)

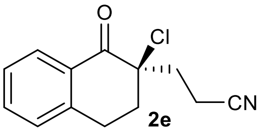

The title compound was prepared following **Method A**, using **C1** (7.7 mg, 0.010 mmol), *N*-chlorosuccinimide (20.4 mg, 0.153 mmol), and **1e** (25.1 mg, 0.102 mmol) in toluene (1.0 mL), and the reaction mixture was stirred for 6 h. The crude product was purified by flash column chromatography (hexane : dichloromethane = 1 : 1) to provide the title compound as a white solid (19.6 mg, 83% yield, 98% e.e.).

mp: 57–58 °C; **TLC** (hexane : ethyl acetate = 9 : 1): R_f = 0.09; $[\alpha]_D^{30}$ = +44.9 (*c* 1.0, $CHCl_3$); **^{1}H NMR** (500 MHz, $CDCl_3$): δ 8.07 (d, *J* = 7.6 Hz, 1H), 7.54 (dt, *J* = 7.5, 1.2 Hz, 1H), 7.36 (t, *J* = 7.6 Hz, 1H), 7.27 (d, *J* = 7.6 Hz, 1H), 3.39 (ddd, *J* = 16.4, 11.1, 4.6 Hz, 1H), 2.96 (dt, *J* = 17.6, 3.8 Hz, 1H), 2.83–2.76 (m, 1H), 2.71–2.63 (m, 2H), 2.51 (dt, *J* = 14.3, 3.9 Hz, 1H), 2.39–2.30 (m, 2H); **^{13}C NMR** (126 MHz, $CDCl_3$): δ 190.4, 142.4, 134.3, 129.5, 128.9, 128.7, 127.2, 119.3, 68.9, 35.7, 34.3, 25.5, 13.0; **IR** (neat): 2954, 2928, 2250, 1688, 1603, 1457, 1431, 1304, 1238, 818, 745 cm^{-1}; **HRMS** (DART): $[M+H]^+$calcd. for $C_{13}H_{13}Cl_1N_1O_1$, 234.0686; found, 234.0684.

The enantiomeric purity of the title compound was determined by HPLC analyses (DAICEL CHIRALCEL OD-H (0.46 cmϕ × 25 cm), hexane : 2-propanol = 20 : 1, flow rate = 1.0 mL/min, retention time; 29.3min (major) and 24.0 min (minor)).

3,6-dichloro-3-methylchroman-4-one (2f)

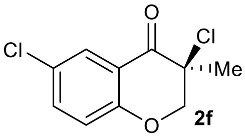

The title compound was prepared following **Method A**, using **C1** (9.0 mg, 0.012 mmol), *N*-chlorosuccinimide (23.9 mg, 0.179 mmol), and **1f** (28.6 mg, 0.119 mmol) in toluene (0.60

mL), and the reaction mixture was stirred for 16 h. The crude product was purified by flash column chromatography (hexane : ethyl acetate = 20 : 1) to provide the title compound as a white solid (25.5 mg, 93% yield, 93% e.e.).

mp: 59 °C; **TLC** (hexane : ethyl acetate = 9 : 1): R_f = 0.39; $[\alpha]_D^{28}$ = +29.5 (*c* 1.3, $CHCl_3$); **^{1}H NMR** (500 MHz, $CDCl_3$): δ 7.90 (d, *J* = 2.7 Hz, 1H), 7.47 (dd, *J* = 8.8, 2.7 Hz, 1H), 7.00 (d, *J* = 9.2 Hz, 1H), 4.53 (d, *J* = 12.6 Hz, 1H), 4.34 (d, *J* = 12.6 Hz, 1H), 1.75 (s, 3H); **^{13}C NMR** (126 MHz, $CDCl_3$): δ 185.5, 159.0, 136.3, 127.8, 127.6, 119.6, 119.0, 75.6, 63.0, 21.0; **IR** (neat): 3387, 3072, 2984, 2927, 2855, 1709, 1604, 1477, 1421, 1287, 1268, 1158, 1116, 1039, 822, 697, 615, 519 cm^{-1}; **HRMS** (DART): $[M+H]^+$calcd. for $C_{10}H_9Cl_2O_2$, 230.9980; found, 230.9978.

The enantiomeric purity of the title compound was determined by HPLC analyses (DAICEL CHIRALCEL OD-H (0.46 cmϕ × 25 cm), hexane : 2-propanol = 500 : 1, flow rate = 1.0 mL/min, retention time; 11.0 min (major) and 14.4 min (minor)).

2-chloro-2-methyl-2,3-dihydro-1*H*-inden-1-one (2g)[14]

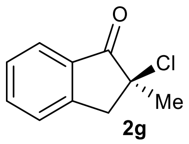

2g

The title compound was prepared following **Method A**, using **C1** (8.6 mg, 0.011 mmol), *N*-chlorosuccinimide (22.7 mg, 0.170 mmol), and **1g** (19.0 mg, 0.113 mmol) in toluene (0.57 mL), and the reaction mixture was stirred at –20 °C for 48 h. The crude product was purified by flash column chromatography (hexane : dichloromethane = 2 : 1) to provide the title compound as a white solid (17.6 mg, 97% yield, 90% e.e.).

mp: 75–76 °C; **TLC** (hexane : ethyl acetate = 9 : 1): R_f = 0.36; $[\alpha]_D^{25}$ = −27.1 (*c* 1.0, $CHCl_3$); **^{1}H NMR** (500 MHz, $CDCl_3$): δ 7.84 (d, *J* = 7.3 Hz, 1H), 7.67 (dt, *J* = 14.9, 1.2 Hz, 1H), 7.45–7.43 (m, 2H), 3.65 (d, *J* = 18.0 Hz, 1H), 3.46 (d, *J* = 18.0 Hz, 1H), 1.80 (s, 3H); **^{13}C NMR** (126 MHz, $CDCl_3$): δ 200.5, 149.7, 136.1, 133.0, 128.4, 126.5, 125.7, 66.8, 45.6, 26.3; **IR** (neat): 2980, 2930, 1724, 1603, 1465, 1331, 1286, 1218, 1055, 977, 797, 749, 624, 529 cm^{-1}; **HRMS** (DART): $[M+NH_4]^+$calcd. for $C_{10}H_{13}Cl_1N_1O_1$, 198.0686; found, 198.0684.

The enantiomeric purity of the title compound was determined by HPLC analyses (DAICEL CHIRALCEL OD-H (0.46 cmϕ × 25 cm), hexane : 2-propanol = 500 : 1, flow rate = 1.0 mL/min, retention time; 10.7 min (major) and 12.4 min (minor)).

5-bromo-2-chloro-2-methyl-2,3-dihydro-1*H*-inden-1-one (2h)

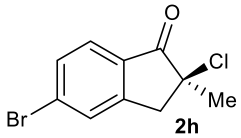

The title compound was prepared following **Method A**, using **C1** (8.6 mg, 0.011 mmol), *N*-chlorosuccinimide (22.7 mg, 0.170 mmol), and **1h** (30.4 mg, 0.113 mmol) in toluene (0.57 mL), and the reaction mixture was stirred at –30 °C for 72 h. The crude product was purified by flash column chromatography (hexane : dichloromethane = 2 : 1) to provide the title compound as a white solid (28.4 mg, 97% yield, 89% e.e.).

mp: 50–51 °C; **TLC** (hexane : ethyl acetate = 9 : 1): R_f = 0.43; $[\alpha]_D^{28}$ = −55.9 (*c* 1.2, $CHCl_3$); **^{1}H NMR** (500 MHz, $CDCl_3$): δ 7.71 (d, *J* = 8.4 Hz, 1H), 7.62 (s, 1H), 7.60–7.58 (m, 1H), 3.63 (d, *J* = 18.0 Hz, 1H), 3.43 (d, *J* = 18.0 Hz, 1H), 1.80 (s, 3H); **^{13}C NMR** (126 MHz, $CDCl_3$): δ 199.2, 151.1, 132.0, 132.0, 131.4, 129.7, 126.8, 66.4, 45.0, 26.0; **IR** (neat): 2969, 2927, 2859, 1732, 1595, 1427, 1322, 1264, 1215, 1056, 975, 865, 830, 654 cm^{-1}; **HRMS** (DART): $[M+NH_4]^+$calcd. for $C_{10}H_{12}Br_1Cl_1N_1O_1$, 275.9791; found, 275.9790.

The enantiomeric purity of the title compound was determined by HPLC analyses (DAICEL CHIRALCEL OD-H (0.46 cmϕ × 25 cm), hexane : 2-propanol = 500 : 1, flow rate = 1.0 mL/min, retention time; 14.1 min (major) and 15.6 min (minor)).

2-chloro-1,2-diphenylpropan-1-one (2i)[16]

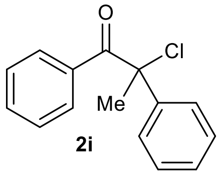

The title compound was prepared following **Method A**, using **C1** (8.1 mg, 0.011 mmol), *N*-chlorosuccinimide (21.4 mg, 0.160 mmol), and **1i** (27.2 mg, 0.107 mmol) in toluene (0.54 mL), and the reaction mixture was stirred for 2.5 h. The crude product was purified by flash column chromatography (hexane : diethyl ether = 99 : 1 to 90 : 10) to provide the title compound as a white solid (25.1 mg, 96% yield, 48% e.e.).

mp: 41–42 °C; **TLC** (hexane : dichloromethane = 2 : 1): R_f = 0.44; $[\alpha]_D^{28}$ = −120.6 (*c* 0.73, $CHCl_3$); **^{1}H NMR** (500 MHz, $CDCl_3$) : δ 7.76–7.74 (m, 2H), 7.51–7.48 (m, 2H), 7.42–7.35 (m, 3H), 7.33–7.29 (m, 1H), 7.27–7.24 (m, 2H), 2.04 (s, 3H); **^{13}C NMR** (126 MHz, $CDCl_3$): δ 194.3, 141.6, 133.6, 132.6, 130.8, 128.9, 128.1, 127.8, 125.3, 73.6, 33.2; **IR** (neat): 3063, 3026, 2996, 2931, 1687, 1596, 1492, 1446, 1373, 1249, 1184, 1055, 958, 838, 761, 701, 599 cm^{-1}; **HRMS** (DART): $[M+NH_4]^+$calcd. for $C_{15}H_{17}Cl_1N_1O_1$, 262.0999; found, 262.0999.

The enantiomeric purity of the title compound was determined by HPLC analyses (DAICEL CHIRALPAK IB-3 (0.46 cmϕ × 25 cm), hexane : 2-propanol = 500 : 1, flow rate = 0.5 mL/min, retention time; 11.4 min (major) and 12.4 min (minor)).

2-benzyl-2-chlorocyclohexan-1-one (2j)

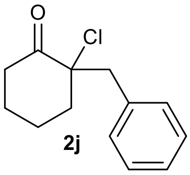

2j

The title compound was prepared following **Method A**, using **C1** (9.3 mg, 0.012 mmol), *N*-chlorosuccinimide (24.6 mg, 0.185 mmol), and **1j** (28.5 mg, 0.123 mmol) in toluene (0.62 mL), and the reaction mixture was stirred at 25 °C for 1.5 h. The crude product was purified by flash column chromatography (hexane : ethyl acetate = 20 : 1) to provide the title compound as a colourless oil (26.6 mg, 97% yield, 40% e.e.).

TLC (hexane : ethyl acetate = 9 : 1): R_f = 0.38; $[\alpha]_D^{30}$ = −53.8 (*c* 0.50, $CHCl_3$); **^{1}H NMR** (500 MHz, $CDCl_3$): δ 7.31–7.23 (m, 5H), 3.31 (d, *J* = 14.5 Hz, 1H), 3.23 (d, *J* = 14.5 Hz, 1H), 3.07 (dt, *J* = 14.0, 6.1 Hz, 1H), 2.41–2.36 (m, 1H), 2.08 (dq, *J* = 14.7, 3.1 Hz, 1H), 2.05–1.91 (m, 2H), 1.81 (ddd, *J* = 14.7, 12.0, 3.8 Hz, 1H), 1.71–1.54 (m, 2H); **^{13}C NMR** (126 MHz, $CDCl_3$): δ 204.7, 135.6, 131.3, 127.9, 126.9, 73.1, 43.7, 39.3, 37.3, 26.5, 20.8; **IR** (neat): 3030, 2942, 2863, 1721, 1494, 1451, 1434, 1124, 1082, 735, 699, 602 cm^{-1}; **HRMS** (DART): $[M+H]^+$calcd. for $C_{13}H_{16}Cl_1O_1$, 223.0890; found, 223.0890.

The enantiomeric purity of the title compound was determined by HPLC analyses (DAICEL CHIRALPAK IC-3 (0.46 cmϕ × 25 cm), hexane : 2-propanol = 500 : 1, flow rate = 1.0 mL/min, retention time; 19.0 min (major) and 20.7 min (minor)).

(*R*)-2-chloro-1-phenylpropan-1-one (2k)[17]

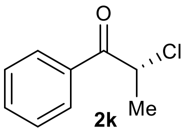

2k

The title compound was prepared following **Method B**, using **C1** (45.0 mg, 0.0594 mmol), *N*-chlorosuccinimide (7.9 + 87.2 mg, 0.059 + 0.653 mmol), and **1k** (105.8 mg, 0.594 mmol) in toluene (3.0 + 9.0 mL). The crude product was purified by flash column chromatography (hexane : dichloromethane = 2 : 1) to provide the title compound as a colourless oil (87.1 mg, 87% yield, 85% e.e.), along with a 5% yield of α,α-dichloroketone **3**.

TLC (hexane : dichloromethane = 2 : 1): R_f = 0.24; $[\alpha]_D^{29}$ = −11.0 (*c* 0.85, $CHCl_3$); **^{1}H NMR**

(500 MHz, $CDCl_3$): δ 8.03 (dd, *J* = 7.6, 1.2 Hz, 2H), 7.61 (dt, *J* = 7.6, 1.2 Hz, 1H), 7.50 (t, *J* = 7.6 Hz, 2H), 5.26 (q, *J* = 6.5 Hz, 1H), 1.75 (d, *J* = 6.5 Hz, 3H); **^{13}C NMR** (126 MHz, $CDCl_3$): δ 193.5, 134.0, 133.7, 128.9, 128.7, 52.7, 19.9; **IR** (neat): 2931, 1698, 1599, 1443, 1340, 1249, 1200, 998, 959, 720, 689 cm^{-1}; **HRMS** (DART): $[M+H]^+$calcd. for $C_9H_{10}Cl_1O_1$, 169.0420; found, 169.0421.

The enantiomeric purity of the title compound was determined by HPLC analyses (DAICEL CHIRALPAK IA-3 (0.46 cmϕ × 25 cm), hexane : 2-propanol = 500 : 1, flow rate = 1.0 mL/min, retention time; 9.7 min (major) and 11.9 min (minor)).

The absolute configuration of the major enantiomer of **2k** was estimated to be *R* by comparison with the specific rotation and the retention time on HPLC analyses of its derivative *N*-Boc-Cathinone **11**.

2,2-dichloro-1-phenylpropan-1-one (3)[18]

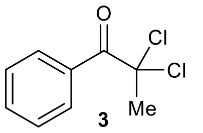

3

TLC (hexane : dichloromethane = 2 : 1): R_f = 0.66; **^{1}H NMR** (500 MHz, $CDCl_3$): δ 8.32 (d, *J* = 7.6 Hz, 2H), 7.60 (t, *J* = 7.6 Hz, 1H), 7.48 (t, *J* = 7.6 Hz, 2H), 2.36 (s, 3H); **^{13}C NMR** (126 MHz, $CDCl_3$): δ 188.0, 133.6, 131.2, 131.1, 128.1, 82.7, 34.2; **HRMS** (DART): $[M+H]^+$calcd. for $C_9H_9Cl_2O_1$, 203.0031; found, 203.0031.

2-chloro-1-phenylpent-4-en-1-one (2l)

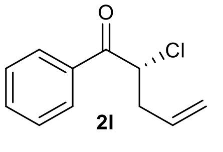

2l

The title compound was prepared following **Method B**, using **C1** (7.8 mg, 0.010 mmol), *N*-chlorosuccinimide (1.4 + 15.1 mg, 0.011 + 0.113 mmol), and **1l** (21.0 mg, 0.103 mmol) in toluene (0.52 + 1.6 mL). The crude product was purified by flash column chromatography (hexane : dichloromethane = 2 : 1) to provide the title compound as a colourless oil (14.8 mg, 74% yield, 83% e.e.).

TLC (hexane : dichloromethane = 2 : 1): R_f = 0.29; $[\alpha]_D^{30} = -11.5$ (*c* 0.70, $CHCl_3$); **^{1}H NMR** (500 MHz, $CDCl_3$): δ 8.01 (d, *J* = 7.3 Hz, 2H), 7.61 (t, *J* = 7.3 Hz, 1H), 7.50 (t, *J* = 7.3 Hz, 2H), 5.88 (ddt, *J* = 17.0, 10.3, 6.9 Hz, 1H), 5.20 (dq, *J* = 17.0, 1.2 Hz, 1H), 5.16 (dq, *J* = 10.3, 1.2 Hz, 1H), 5.12 (dd, *J* = 7.6, 6.5 Hz, 1H), 2.92 (dt, *J* = 14.5, 6.5 Hz, 1H), 2.76 (dt, *J* = 14.5, 7.6 Hz, 1H); **^{13}C NMR** (126 MHz, $CDCl_3$): δ 193.0, 134.4, 133.8, 132.9, 128.9, 128.8, 119.0, 56.2,

37.7; **IR** (neat): 3078, 2926, 1698, 1592, 1452, 1278, 1248, 1210, 1179, 998, 923, 824, 685 cm^{-1}; **HRMS** (DART): $[M+H]^+$calcd. for $C_{11}H_{12}Cl_1O_1$, 195.0577; found, 195.0577.

The enantiomeric purity of the the title compound was determined by HPLC analyses (DAICEL CHIRALPAK IA-3 (0.46 cmϕ × 25 cm), hexane : 2-propanol = 500 : 1, flow rate = 1.0 mL/min, retention time; 9.1 min (major) and 10.1 min (minor)).

2-chloro-1,3-diphenylpropan-1-one (2m)

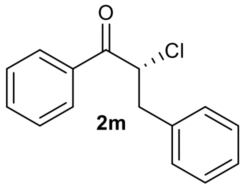

The title compound was prepared following **Method B**, using **C1** (8.6 mg, 0.011 mmol), *N*-chlorosuccinimide (1.6 + 16.6 mg, 0.012 + 0.124 mmol), and **1m** (28.8 mg, 0.113 mmol) in toluene (0.55 + 1.7 mL). The crude product was purified by flash column chromatography (hexane : dichloromethane = 2 : 1) to provide the title compound as a colourless oil (22.1 mg, 79% yield, 86% e.e.).

TLC (hexane : dichloromethane = 2 : 1): R_f = 0.30; $[\alpha]_D^{29}$ = −33.1 (*c* 0.95, $CHCl_3$); **^{1}H NMR** (500 MHz, $CDCl_3$): δ 7.97 (d, *J* = 7.3 Hz, 2H), 7.58 (t, *J* = 7.3 Hz, 1H), 7.46 (t, *J* = 7.3 Hz, 2H), 7.31–7.21 (m, 5H), 5.28 (dd, *J* = 7.6, 6.9 Hz, 1H), 3.55 (dd, *J* = 14.1, 6.9 Hz, 1H), 3.25 (dd, *J* = 14.1, 7.6 Hz, 1H); **^{13}C NMR** (126 MHz, $CDCl_3$): δ 193.0, 136.7, 134.4, 133.8, 129.5, 128.9, 128.8, 128.6, 127.1, 57.2, 39.5; **IR** (neat): 3062, 3025, 2930, 1690, 1600, 1584, 1490, 1444, 1281, 1240, 1179, 976, 752, 696, 685, 661 cm^{-1}; **HRMS** (DART): $[M+H]^+$calcd. for $C_{15}H_{14}Cl_1O_1$, 245.0733; found, 245.0733.

The enantiomeric purity of the title compound was determined by HPLC analyses (DAICEL CHIRALPAK IA-3 (0.46 cmϕ × 25 cm), hexane : 2-propanol = 500 : 1, flow rate = 1.0 mL/min, retention time; 11.6 min (major) and 12.7 min (minor)).

2-chloro-1-(*p*-tolyl)propan-1-one (2n)[17]

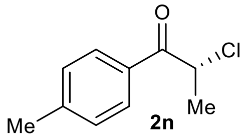

The title compound was prepared following **Method B**, using **C1** (8.3 mg, 0.011 mmol), *N*-chlorosuccinimide (1.4 + 16.1 mg, 0.011 + 0.120 mmol), and **1n** (21.0 mg, 0.109 mmol) in toluene (0.55 + 1.65 mL). The crude product was purified by flash column chromatography (hexane : dichloromethane = 2 : 1) to provide the title compound as a white solid (15.2 mg, 76%

yield, 82% e.e.).

mp: 47 °C; **TLC** (hexane : dichloromethane = 2 : 1): R_f = 0.27; $[\alpha]_D^{30}$ = −23.3 (*c* 0.67, $CHCl_3$); **^{1}H NMR** (500 MHz, $CDCl_3$): δ 7.92 (d, *J* = 8.0 Hz, 2H), 7.29 (d, *J* = 8.0 Hz, 2H), 5.24 (q, *J* = 6.5 Hz, 1H), 2.43 (s, 3H), 1.74 (d, *J* = 6.5 Hz, 3H); **^{13}C NMR** (126 MHz, $CDCl_3$): δ 193.3, 144.8, 131.6, 129.5, 129.0, 52.8, 21.7, 20.0; **IR** (neat): 2934, 1683, 1603, 1451, 1348, 1249, 1180, 957, 830, 750, 609 cm^{-1}; **HRMS** (DART): $[M+H]^+$calcd. for $C_{10}H_{12}$ Cl_1O_1, 183.0577; found, 183.0576.

The enantiomeric purity of the title compound was determined by HPLC analyses (DAICEL CHIRALPAK IA-3 (0.46 cmϕ × 25 cm), hexane : 2-propanol = 500 : 1, flow rate = 1.0 mL/min, retention time; 12.0 min (major) and 14.7 min (minor)).

2-chloro-1-(4-fluorophenyl)propan-1-one (2o)[19]

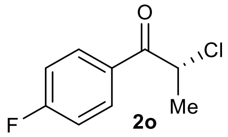

2o

The title compound was prepared following **Method B**, using **C1** (9.3 mg, 0.012 mmol), *N*-chlorosuccinimide (1.6 + 18.0 mg, 0.012 + 0.135 mmol), and **1o** (24.1 mg, 0.123 mmol) in toluene (0.61 + 1.8 mL). The crude product was purified by flash column chromatography (hexane : dichloromethane = 2 : 1) to provide the title compound as a colourless oil (17.9 mg, 78% yield, 78% e.e.).

TLC (hexane : dichloromethane = 2 : 1): R_f = 0.40; $[\alpha]_D^{29}$ = −14.0 (*c* 0.70, $CHCl_3$); **^{1}H NMR** (500 MHz, $CDCl_3$): δ 8.06 (dd, *J* = 9.2, 5.4 Hz, 2H), 7.17 (dt, *J* = 9.2, 1.9 Hz, 2H), 5.19 (q, *J* = 6.9 Hz, 1H), 1.75 (d, *J* = 6.9 Hz, 3H); **^{13}C NMR** (126 MHz, $CDCl_3$): δ 192.0, 166.0 (d, *J* = 256.7 Hz), 131.8 (d, *J* = 8.4 Hz), 130.5 (d, *J* = 2.4 Hz), 116.0 (d, *J* = 22.8 Hz), 52.6, 19.8; **^{19}F NMR** (470 MHz, $CDCl_3$): δ −104.2; **IR** (neat): 2930, 1698, 1596, 1511, 1346, 1244, 1165, 953, 843, 764, 590 cm^{-1}; **HRMS** (DART): $[M+H]^+$calcd. for $C_9H_9Cl_1F_1O_1$, 187.0326; found, 187.0325.

The enantiomeric purity of the title compound was determined by HPLC analyses (DAICEL CHIRALPAK IA-3 (0.46 cmϕ × 25 cm), hexane : 2-propanol = 500 : 1, flow rate = 1.0 mL/min, retention time; 9.8 min (major) and 11.1 min (minor)).

2-chloro-1-(3-chlorophenyl)propan-1-one (2p)

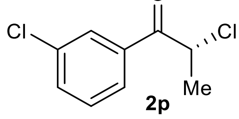

2p

The title compound was prepared following **Method B**, using **C1** (8.4 mg, 0.011 mmol), *N*-chlorosuccinimide (1.5 + 16.3 mg, 0.011 + 0.122 mmol), and **1p** (23.6 mg, 0.111 mmol) in toluene (0.56 + 1.7 mL). The crude product was purified by flash column chromatography (hexane : dichloromethane = 4 : 1) to provide the title compound as a colourless oil (17.8 mg, 79% yield, 79% e.e.).

TLC (hexane : dichloromethane = 2 : 1): R_f = 0.41; $[\alpha]_D^{30} = -15.6$ (*c* 0.84, $CHCl_3$); **^{1}H NMR** (500 MHz, $CDCl_3$): δ 7.99 (t, *J* = 1.9 Hz, 1H), 7.89 (dd, *J* = 8.0, 1.2 Hz, 1H), 7.58 (ddd, *J* = 8.0, 1.9, 1.2 Hz, 1H), 7.44 (t, *J* = 8.0 Hz, 1H), 5.18 (q, *J* = 6.5 Hz, 1H), 1.75 (d, *J* = 6.5 Hz, 3H); **^{13}C NMR** (126 MHz, $CDCl_3$): δ 192.4, 135.7, 135.1, 133.6, 130.0, 129.1, 127.0, 52.6, 19.7; **IR** (neat): 2930, 1698, 1573, 1417, 1338, 1240, 1190, 976, 805, 752, 696, 673 cm^{-1}; **HRMS** (DART): $[M+H]^+$calcd. for $C_9H_9Cl_2O_1$, 203.0031; found, 203.0031.

The enantiomeric purity of the title compound was determined by HPLC analyses (DAICEL CHIRALPAK IA-3 (0.46 cmϕ × 25 cm), hexane : 2-propanol = 500 : 1, flow rate = 1.0 mL/min, retention time; 10.2 min (major) and 10.9 min (minor)).

2-chloro-1-(4-methoxyphenyl)propan-1-one (2q)[17]

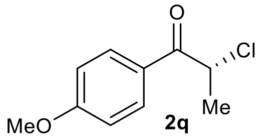

The title compound was prepared following **Method B**, using **C1** (7.8 mg, 0.010 mmol), *N*-chlorosuccinimide (1.4 + 15.1 mg, 0.011 + 0.113 mmol), and **1q** (21.4 mg, 0.103 mmol) in toluene (0.51 + 1.5 mL). The crude product was purified by flash column chromatography (hexane : dichloromethane = 1 : 1) to provide the title compound as a colourless oil (15.6 mg, 76% yield, 83% e.e.).

TLC (hexane : dichloromethane = 2 : 1): R_f = 0.25; $[\alpha]_D^{28} = -47.1$ (*c* 0.68, $CHCl_3$); **^{1}H NMR** (500 MHz, $CDCl_3$): δ 8.01 (dt, *J* = 9.9, 3.1 Hz, 2H), 6.96 (dt, *J* = 9.9, 3.1 Hz, 2H), 5.22 (q, *J* = 6.9 Hz, 1H), 3.89 (s, 3H), 1.73 (d, *J* = 6.9 Hz, 3H); **^{13}C NMR** (126 MHz, $CDCl_3$): δ 192.2, 164.0, 131.4, 126.9, 114.0, 55.5, 52.6, 20.1; **IR** (neat): 2930, 2840, 1686, 1606, 1512, 1342, 1248, 1179, 1033, 957, 840, 608 cm^{-1}; **HRMS** (DART): $[M+H]^+$calcd. for $C_{10}H_{12}Cl_1O_2$, 199.0526; found, 199.0526.

The enantiomeric purity of the title compound was determined by HPLC analyses (DAICEL CHIRALPAK IA-3 (0.46 cmϕ × 25 cm), hexane : 2-propanol = 500 : 1, flow rate = 1.5 mL/min, retention time; 17.5 min (major) and 23.6 min (minor)).

3-chloro-4-phenylbutan-2-one (2r)[20]

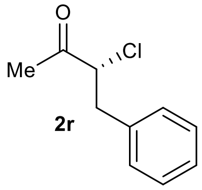

The title compound was prepared following **Method B**, using **C1** (9.3 mg, 0.012 mmol), *N*-chlorosuccinimide (1.6 + 18.0 mg, 0.012 + 0.135 mmol, added slowly over 30 min), and **1r** (23.6 mg, 0.123 mmol) in toluene (0.61 + 1.8 mL). The crude product was purified by flash column chromatography (hexane : ethyl acetate = 20 : 1) to provide the title compound as a colourless oil (16.4 mg, 73% yield, 79% e.e.).

TLC (hexane : ethyl acetate = 10 : 1): R_f = 0.39; $[\alpha]_D^{28}$ = +55.5 (*c* 0.37, $CHCl_3$); **^{1}H NMR** (500 MHz, $CDCl_3$): δ 7.31 (t, *J* = 7.3 Hz, 2H), 7.25 (t, *J* = 7.3 Hz, 1H), 7.21 (d, *J* = 7.3 Hz, 2H), 4.40 (dd, *J* = 8.0, 6.1 Hz, 1H), 3.33 (dd, *J* = 14.3, 6.1 Hz, 1H), 3.07 (dd, *J* = 14.3, 8.0 Hz, 1H), 2.27 (s, 3H); **^{13}C NMR** (126 MHz, $CDCl_3$): δ 202.5, 136.1, 129.3, 128.6, 127.2, 63.7, 39.7, 26.8; **IR** (neat): 3029, 2923, 1724, 1501, 1456, 1433, 1358, 1236, 1157, 748, 699 cm^{-1}; **HRMS** (DART): $[M+H]^+$calcd. for $C_{10}H_{12}Cl_1O_1$, 183.0577; found, 183.0576.

The enantiomeric purity of the title compound was determined by HPLC analyses (DAICEL CHIRALPAK IA-3 (0.46 cmϕ × 25 cm), hexane : 2-propanol = 500 : 1, flow rate = 1.0 mL/min, retention time; 10.3 min (major) and 12.3 min (minor)).

2-chloro-3,4-dihydronaphthalen-1(2*H*)-one (2s)[21]

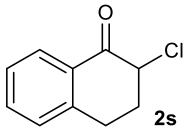

The title compound was prepared following **Method B**, using **C1** (12.6 mg, 0.0166 mmol), *N*-chlorosuccinimide (2.2 + 24.4 mg, 0.017 + 0.182 mmol), and **1s** (31.6 mg, 0.166 mmol) in toluene (0.83 + 2.5 mL). After the completion of adding *N*-chlorosuccinimide, the reaction mixture was stirred for another 2 h. The crude product was purified by flash column chromatography (hexane : dichloromethane = 2 : 1) to provide the title compound as a white solid (27.6 mg, 92% yield, 56% e.e.).

mp: 42 °C; **TLC** (hexane : ethyl acetate = 9 : 1): R_f = 0.34; $[\alpha]_D^{29}$ = −1.5 (*c* 0.41, $CHCl_3$); **^{1}H NMR** (500 MHz, $CDCl_3$): δ 8.09 (d, *J* = 8.0 Hz, 1H), 7.53 (dd, *J* = 8.0, 1.2 Hz, 1H), 7.35 (d, *J* = 8.0 Hz, 1H), 7.28 (d, *J* = 8.0 Hz, 1H), 4.64 (dd, *J* = 7.6, 3.8 Hz, 1H), 3.29 (ddd, *J* = 16.8, 8.0, 4.6 Hz, 1H), 3.00 (ddd, *J* = 17.2, 6.9, 4.6 Hz, 1H), 2.59 (dddd, *J* = 16.8, 6.9, 4.6, 3.8 Hz, 1H), 2.46 (dddd, *J* = 17.2, 8.0, 7.6, 4.6 Hz, 1H); **^{13}C NMR** (126 MHz, $CDCl_3$): δ 190.8, 143.1, 134.1,

130.4, 128.7, 128.5, 127.1, 59.8, 32.4, 26.3; **IR** (neat): 3067, 2942, 1691, 1599, 1451, 1435, 1307, 1218, 895, 800, 740, 621, 523 cm^{-1}; **HRMS** (DART): $[M+H]^+$calcd. for $C_{10}H_{10}Cl_1O_1$, 181.0420; found, 181.0418.

The enantiomeric purity of the title compound was determined by HPLC analyses (DAICEL CHIRALCEL OD-H (0.46 cmϕ × 25 cm), hexane : 2-propanol = 500 : 1, flow rate = 0.9 mL/min, retention time; 22.9 min (major) and 20.8 min (minor)).

Experimental procudure for Fig. 4c and 4d.

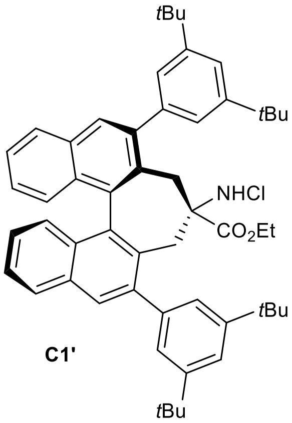

C1'

Enantioselective decarboxylative chlorination of **1a** (104.6 mg, 0.512 mmol) was performed by **Method A** with 10 mol% **C1** (0.0512 mmol). After the reaction completed, 24% yield of **C1** (9.4 mg) was recovered by flash column chromatography (hexane : dichloromethane = 3 : 2 to 1 : 4), along with a mixture of **2a** and **C1'**. Then, the mixture was purified again by flash column chromatography (hexane : dichloromethane = 4 : 1 to 1 : 1) to give **C1'** as a white solid (27.6 mg, 68% yield based on **C1**).

mp: 168–169 °C; **TLC** (hexane : dichloromethane = 2 : 1): R_f = 0.50; $[\alpha]_D^{13}$ = +58.5 (*c* 3.10, $CHCl_3$); **^{1}H NMR** (500 MHz, $CDCl_3$): δ 8.03 (d, *J* = 36.7 Hz, 2H), 7.74 (dd, *J* = 19.1, 8.0 Hz, 2H), 7.58 (dd, *J* = 22.4, 8.6 Hz, 2H), 7.55 (d, *J* = 27.5 Hz, 2H), 7.44 (bs, 4H), 7.21 (quint., *J* = 6.5 Hz, 2H), 6.97 (q, *J* = 7.6 Hz, 2H), 4.09 (s, 1H), 3.93 (d, *J* = 14.5 Hz, 1H), 3.84 (d, *J* = 13.0 Hz, 1H), 3.82–3.76 (m, 1H), 3.36–3.29 (m, 2H), 2.63 (d, *J* = 13.0 Hz, 1H), 1.48 (s, 9H), 1.30 (s, 27H); **^{13}C NMR** (126 MHz, $CDCl_3$): δ 171.1, 151.1–150.3, 143.6, 141.5, 141.4, 141.1, 137.0, 135.9, 133.8, 133.3, 133.2, 131.9, 131.8, 131.7, 130.2, 129.4, 128.6, 128.6, 128.3, 127.6, 126.4, 126.3, 126.1, 126.0, 125.2, 121.1, 120.6, 79.1, 61.0, 35.8, 35.0, 31.6, 30.9, 13.8; **IR** (neat): 2966, 2900, 2866, 1746, 1592, 1476, 1445, 1395, 1364, 1272, 1249, 1175, 1052, 882, 747, 716 cm^{-1}; **HRMS** (DART): $[M+H]^+$calcd. for for $C_{54}H_{63}Cl_1N_1O_2$, 792.4547; found, 792.4549.

(*R*)-2-methyl-3,4-dihydronaphthalen-1(2*H*)-one (5a)[22]

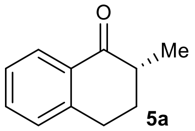

To a stirred solution of amine catalyst **C1** (24.2 mg, 0.0319 mmol) in toluene (1.6 mL) was added **1a** (65.1 mg, 0.319 mmol). Then, the reaction mixture was stirred at 15 °C for 24 h in the dark. The crude product was purified by flash column chromatography (hexane : ethyl acetate = 20 : 1 to 4 : 1) to provide the title compound as a colourless oil (48.3 mg, 78% yield, 64% e.e.).
The enantiomeric purity of the title compound was determined by HPLC analyses (DAICEL CHIRALCEL OD-H (0.46 cmϕ × 25 cm), hexane : 2-propanol = 100 : 1, flow rate = 0.5 mL/min, retention time; 12.3 min (major) and 13.6 min (minor)).
The absolute configuration of the major enantiomer of **5a** was determined to be *R* by comparing the retention times on chiral HPLC with those in the literature.[22]

S_N2 Reaction of α-chloroketones 2 with sodium azide (Fig. 5).

2-azido-2-methyl-3,4-dihydronaphthalen-1(2*H*)-one (7)[23]

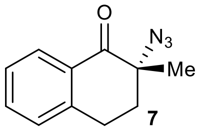

To a stirred solution of **2a** (122.7 mg, 0.630 mmol, 96% e.e.) in DMSO (2.5 mL) was added NaN_3 (81.9 mg, 1.26 mmol), and the reaction mixture was stirred at 80 °C for 20 min. Diethyl ether and water were added to the mixture, and the mixture was washed with water. The combined organic layer was dried over anhydrous Na_2SO_4, concentrated, and then purified by flash column chromatography on silica gel (hexane : ethyl acetate = 10 : 1) to give 7 as a colourless (106.7 mg, 84% yield, 96% e.e.).
TLC (hexane : dichloromethane = 2 : 1): R_f = 0.32; $[\alpha]_D^{27}$ = +255.1 (*c* 1.0, $CHCl_3$); **^{1}H NMR** (500 MHz, $CDCl_3$): δ 8.09 (dd, *J* = 8.0, 1.2 Hz, 1H), 7.52 (dt, *J* = 7.5, 1.4 Hz, 1H), 7.35 (dt, *J* = 7.3, 0.8 Hz, 1H), 7.25 (d, *J* = 8.0 Hz, 1H), 3.13 (ddd, *J* = 17.2, 8.0, 5.0 Hz, 1H), 2.91 (ddd, *J* = 17.1, 7.1, 4.9 Hz, 1H), 2.21 (ddd, *J* = 13.8, 7.3, 4.6 Hz, 1H), 2.09 (ddd, *J* = 13.7, 7.9, 4.9 Hz, 1H), 1.58 (s, 3H); **^{13}C NMR** (126 MHz, $CDCl_3$): δ 194.7, 143.4, 134.2, 130.4, 128.8, 128.7, 127.2, 64.6, 35.0, 25.7, 20.4; **IR** (neat): 2977, 2934, 2107, 1688, 1600, 1458, 1430, 1378, 1308, 1228, 895, 800, 743, 700 cm^{-1}; **HRMS** (DART): $[M+NH_4]^+$calcd. for $C_{11}H_{15}N_4O_1$, 219.1246; found, 219.1244.
The enantiomeric purity of the title compound was determined by HPLC analyses (DAICEL

CHIRALPAK IC-3 (0.46 cmϕ × 25 cm), hexane : 2-propanol = 500 : 1, flow rate = 1.0 mL/min, retention time; 35.7 min (major) and 39.2 min (minor)).

***tert*-butyl (2-methyl-1-oxo-1,2,3,4-tetrahydronaphthalen-2-yl)carbamate (8)**

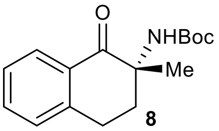

8

To a stirred suspension of Pd/C (Palladium assay 10%, 2.9 mg, 0.027 mmol) in ethyl acetate (6.1 mL) was added a solution of **7** (110.3 mg, 0.548 mmol, 96% e.e.) and di-*tert*-butyl dicarbonate (143.6 mg, 0.658 mmol) in ethyl acetate (12.2 mL) under a hydrogen atmosphere. Then, the reaction mixture was stirred at 25 °C for 15 min. The mixture was filtered, and the filtrate was concentrated, and then purified by flash column chromatography on silica gel (hexane : ethyl acetate = 9 : 1) to give **8** as a white solid (139.4 mg, 92% yield, 96% e.e.).
mp: 95–96 °C; **TLC** (hexane : ethyl acetate = 9 : 1): R_f = 0.24; $[\alpha]_D^{30}$ = –41.3 (*c* 0.61, $CHCl_3$); **^{1}H NMR** (500 MHz, $CDCl_3$): δ 8.05 (dd, *J* = 7.6, 1.2 Hz, 1H), 7.49 (dt, *J* = 7.5, 1.3 Hz, 1H), 7.32 (t, *J* = 7.5 Hz, 1H), 7.23 (d, *J* = 8.0 Hz, 1H), 5.84 (bs, 1H), 3.09 (ddd, *J* = 17.8, 12.8, 5.0 Hz, 1H), 2.96 (ddd, *J* = 17.7, 5.3, 2.3 Hz, 1H), 2.84 (bs, 1H), 2.36 (dt, *J* = 13.2, 5.4 Hz, 1H), 1.51 (s, 3H), 1.45 (s, 9H); **^{13}C NMR** (126 MHz, $CDCl_3$): δ 197.9, 154.6, 142.8, 133.7, 130.3, 128.7, 128.4, 126.7, 79.3, 58.6, 33.1, 28.4, 26.2, 20.4; **IR** (neat): 3410, 2977, 2931, 1717, 1692, 1606, 1491, 1453, 1313, 1170, 1066, 975, 740 cm^{-1}; **HRMS** (DART): $[M+H]^+$calcd. for $C_{16}H_{22}N_1O_3$, 276.1600; found, 276.1602.
The enantiomeric purity of the title compound was determined by HPLC analyses (DAICEL CHIRALCEL OJ-H (0.46 cmϕ × 25 cm), hexane : 2-propanol = 20 : 1, flow rate = 1.0 mL/min, retention time; 6.7 min (major) and 8.9 min (minor)).

S_N2 Reaction of α-chloroketones 2 with alkyl thiolate (Fig. 5).

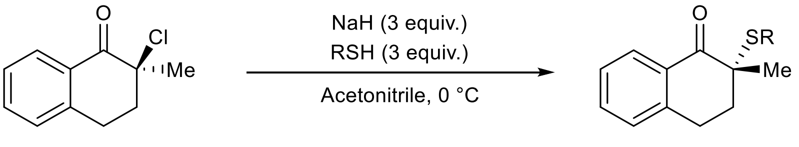

(*S*)-**2a** **9**

General procedure: To a stirred suspension of NaH (60% in oil, washed with hexane, 3.0 equiv.) in acetonitrile was added thiols (3.0 equiv.) at 0 °C, and the mixture was stirred at 0 °C for 1 h. Then, a solution of **2a** (96% e.e.) in acetonitrile was added, and the reaction mixture was attired at 0 °C. The reaction mixture was quenched by adding saturated NH_4Cl aqueous solution at 0 °C, and then extracted with diethyl ether. The combined organic layer was dried over

anhydrous Na_2SO_4, concentrated, and then purified by flash column chromatography on silica gel to give sulfenyl ketone **9**.

(*R*)-2-methyl-2-(phenylthio)-3,4-dihydronaphthalen-1(2*H*)-one (9a)[24]

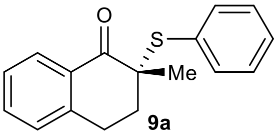

9a

The title compound was synthesised according to **General procedure**, using NaH (36.5 mg, 1.52 mmol), benzenethiol (169.0 mg, 1.52 mmol), and **2a** (98.9 mg, 0.508 mmol, 96% e.e.) in acetonitrile (4.0 mL). The crude product was purified by flash column chromatography (hexane : dichloromethane = 2 : 1 to 1 : 2) to provide the title compound as a pale yellow oil (133.9 mg, 93% yield, 95% e.e.).

mp: 102 °C; **TLC** (hexane : dichloromethane = 2 : 1): R_f = 0.15; $[\alpha]_D^{30}$ = +144.6 (*c* 1.0, $CHCl_3$); **^{1}H NMR** (500 MHz, $CDCl_3$): δ 8.10 (dd, *J* = 8.0, 1.2 Hz, 1H), 7.48 (dt, *J* = 7.5, 1.2 Hz, 1H), 7.39–7.28 (m, 6H), 7.24 (d, *J* = 7.6 Hz, 1H), 3.51–3.41 (m, 1H), 2.88 (dt, *J* = 17.2, 3.8 Hz, 1H), 2.40–2.33 (m, 2H), 1.44 (s, 3H); **^{13}C NMR** (126 MHz, $CDCl_3$): δ 193.3, 142.4, 137.4, 133.0, 131.3, 129.5, 129.4, 128.6, 128.6, 128.3, 126.8, 54.8, 36.1, 25.7, 24.0; **IR** (neat): 3060, 2925, 1678, 1603, 1473, 1454, 1440, 1300, 1230, 963, 903, 747, 690 cm^{-1}; **HRMS** (DART): $[M+H]^+$calcd. for $C_{17}H_{17}O_1S_1$, 269.1000; found, 269.1004.

The enantiomeric purity of the title compound was determined by HPLC analyses (DAICEL CHIRALPAK AD-H (0.46 cmϕ × 25 cm), hexane : 2-propanol = 100 : 1, flow rate = 0.8 mL/min, retention time; 13.1 min (major) and 15.0 min (minor)).

The absolute configuration of the major enantiomer of **9a** was determined to be *R* by X-ray crystallographic analyses (See Supplementary Table 4).

2-methyl-2-(octylthio)-3,4-dihydronaphthalen-1(2*H*)-one (9b)

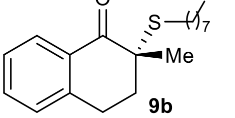

9b

The title compound was synthesised according to **General procedure**, using NaH (37.0 mg, 1.54 mmol), 1-octanethiol (225.3 mg, 1.54 mmol), and **2a** (99.6 mg, 0.512 mmol, 96% e.e.) in acetonitrile (5.1 mL), and the reaction mixture was stirred for 20 min. The crude product was purified by flash column chromatography (hexane : dichloromethane = 2 : 1) to provide the title compound as a colourless oil (149.7 mg, 96% yield, 95% e.e.).

TLC (hexane : dichloromethane = 2 : 1): R_f = 0.28; $[\alpha]_D^{20}$ = +22.9 (*c* 1.2, $CHCl_3$); **^{1}H NMR**

(500 MHz, $CDCl_3$): δ 8.11 (dd, *J* = 8.1, 1.1 Hz, 1H), 7.44 (dt, *J* = 7.5, 1.5 Hz, 1H), 7.30 (t, *J* = 7.6 Hz, 1H), 7.18 (d, *J* = 7.3 Hz, 1H), 3.34 (ddd, *J* = 17.3, 12.7, 5.2 Hz, 1H), 2.77 (qd, *J* = 17.2, 2.3 Hz, 1H), 2.58 (dt, *J* = 11.9, 7.5 Hz, 1H), 2.38–2.28 (m, 2H), 2.23 (qd, *J* = 13.8, 2.3 Hz, 1H), 1.58 (s, 3H), 1.47–1.41 (m, 2H), 1.31–1.21 (m, 10H), 0.86 (t, *J* = 7.3 Hz, 3H); **^{13}C NMR** (126 MHz, $CDCl_3$): δ 192.8, 142.5, 132.8, 130.7, 128.4, 128.3, 126.6, 50.4, 36.5, 31.7, 29.1, 29.1, 29.0, 27.4, 25.6, 23.8, 22.6, 14.0; **IR** (neat): 2958, 2924, 2854, 1677, 1604, 1453, 1376, 1299, 1229, 963, 905, 743 cm^{-1}; **HRMS** (DART): $[M+H]^+$calcd. for $C_{19}H_{29}O_1S_1$, 305.1939; found, 305.1941.

The enantiomeric purity of the title compound was determined by HPLC analyses (DAICEL CHIRALCEL OD-H (0.46 cmϕ × 25 cm), hexane : 2-propanol = 500 : 1, flow rate = 1.0 mL/min, retention time; 8.5 min (major) and 6.9 min (minor)).

2-azido-1-phenylpropan-1-one (10) [25]

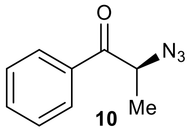

10

To a stirred solution of **2k** (78.0 mg, 0.463 mmol, 85% e.e.) in DMF (1.9 mL) was added NaN_3 (45.1 mg, 0.694 mmol) at −20 °C, and the reaction mixture was stirred at −20 °C for 7 h. Diethyl ether and water were added to the mixture, and the mixture was washed with water. The combined organic layer was dried over anhydrous Na_2SO_4, concentrated, and then purified by flash column chromatography on silica gel (hexane : ethyl acetate = 20 : 1) to give **10** as a colourless oil (75.5 mg, 93% yield, 85% e.e.).

TLC (hexane : ethyl acetate = 10 : 1): R_f = 0.33; $[\alpha]_D^{31}$ = +138.0 (*c* 0.95, $CHCl_3$); **^{1}H NMR** (500 MHz, $CDCl_3$): δ 7.94 (d, *J* = 7.6 Hz, 2H), 7.62 (t, *J* = 7.6 Hz, 1H), 7.50 (t, *J* = 7.6 Hz, 2H), 4.72 (q, *J* = 6.9 Hz, 1H), 1.57 (d, *J* = 6.9 Hz, 3H); **^{13}C NMR** (126 MHz, $CDCl_3$): δ 196.7, 134.2, 133.9, 128.9, 128.6, 58.3, 16.4; **IR** (neat): 2124, 2097, 1690, 1595, 1451, 1379, 1257, 1219, 963, 701 cm^{-1}; **HRMS** (DART): $[M+H]^+$calcd. for $C_9H_{10}N_3O_1$, 176.0824; found, 176.0824.

The enantiomeric purity of the title compound was determined by HPLC analyses (DAICEL CHIRALPAK IC-3 (0.46 cmϕ × 25 cm), hexane : 2-propanol = 500 : 1, flow rate = 1.5 mL/min, retention time; 18.6 min (major) and 21.3 min (minor)).

(*S*)-*N*-Boc-Cathinone (11)[26]

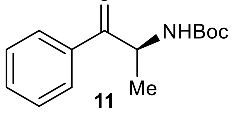

11

To a suspension of Pd/C (Palladium assay 10%, 2.3 mg, 0.022 mmol mmol) in ethyl acetate (7.2 mL) was added a solution of **10** (75.5 mg, 0.431 mmol, 85% e.e.) and di-*tert*-butyl dicarbonate (112.9 mg, 0.517 mmol) in ethyl acetate (7.2 mL) under a hydrogen atmosphere. Then, the reaction mixture was stirred at 25 °C for 15 min. The mixture was filtered, and the filtrate was concentrated, and then purified by flash column chromatography on silica gel (hexane : ethyl acetate = 5 : 1) to give **11** as a white solid (103.8 mg, 97% yield, 84% e.e.). *N*-Boc-Cathinone **11** (28.9 mg, 1.37 mmol, 84% e.e.) was dissolved in a minimum amount of pentane and recrystallized at −20 °C for 5 days. The crystal was filtered and the filtrate was concentrated to give 94% e.e. of **11** (23.7 mg, 82% yield).

mp: 79–80 °C; **TLC** (hexane : ehyl acetate = 10 : 1): $R_f = 0.25$; $[\alpha]_D^{26} = -1.8$ (*c* 1.19, $CHCl_3$); **^{1}H NMR** (500 MHz, $CDCl_3$): δ 7.98 (d, *J* = 7.3 Hz, 2H), 7.60 (t, *J* = 7.3 Hz, 1H), 7.49 (t, *J* = 7.3 Hz, 2H), 5.58 (s, 1H), 5.30 (quin, *J* = 7.3 Hz, 1H), 1.46 (s, 9H), 1.40 (d, *J* = 7.3 Hz, 3H); **^{13}C NMR** (126 MHz, $CDCl_3$): δ 199.4, 155.2, 134.2, 133.7, 128.8, 128.6, 79.7, 51.1, 28.3, 19.9: **IR** (neat): 2976, 2926, 1712, 1683, 1505, 1448, 1368, 1255, 1165, 1063, 972, 696 cm^{-1}; **HRMS** (DART): $[M+H]^+$calcd. for $C_{14}H_{20}N_1O_3$, 250.1443; found, 250.1442.

The enantiomeric purity of the title compound was determined by HPLC analyses (DAICEL CHIRALCEL OJ-H (0.46 cmϕ × 25 cm), hexane : 2-propanol = 20 : 1, flow rate = 1.0 mL/min, retention time; 7.0 min (major) and 10.4 min (minor)).

The absolute configuration of the major enantiomer of **11** was determined to be *S* by comparison with the specific rotation and the retention time on HPLC analyses with those reported in the literature.[26]

N-Boc-Cathinone **11** (28.9 mg, 1.37 mmol, 84% e.e.) was dissolved in a minimum amount of pentane and recrystallized at −20 °C for 5 days. The crystal was filtered and the filtrate was concentrated to give 94% e.e. of *N*-Boc-Cathinone (23.7 mg, 82% yield).

Recrystallization of 2a and 9a.

Single crystals of **2a** or **9a** for the X-ray crystallographic analysis prepared by the following procedure. **2a** (96% e.e.) or **9a** (95% e.e.) was dissolved in a minimum amount of diethyl ether, and five times the amount of hexane was added. Then, **2a** or **9a** was recrystallized at −20 °C for 1 or 2 days. The obtained crystal was used for the X-ray crystallographic analyses to determine absolute configuration.

Supplementary References

1. Shibatomi, K., Kitahara, K., Okimi, T., Abe, Y. & Iwasa, S. Enantioselective fluorination

of α-branched aldehydes and subsequent conversion to α-hydroxyacetals via stereospecific C–F bond cleavage. *Chem. Sci.* **7**, 1388–1392 (2016).

2. Shibatomi, K., Muto, T., Sumikawa, Y., Narayama, A. & Iwasa, S. Development of a new chiral spiro oxazolinylpyridine ligand (Spymox) for asymmetric catalysis. *Synlett* 241–244 (2009).
3. McCooey, S. H. & Connon, S. J. Urea- and thiourea-substituted cinchona alkaloid derivatives as highly efficient bifunctional organocatalysts for the asymmetric addition of malonate to nitroalkenes: inversion of configuration at C9 dramatically improves catalyst performance. *Angew. Chem. Int. Ed.* **44**, 6367–6370 (2005).
4. Park, E. J., Kim, M. H. & Kim, D. Y. Enantioselective alkylation of β-keto esters by phase-transfer catalysis using chiral quaternary ammonium salts. *J. Org. Chem.* **69**, 6897–6899 (2004).
5. Ooi, T. *et al*. Highly enantioselective construction of quaternary stereocenters on β-keto esters by phase-transfer catalytic asymmetric alkylation and Michael reaction. *Angew. Chem. Int. Ed.* **42**, 3796–3798 (2003).
6. Gu, X., Zhang, Y., Xu, Z.-J. & Che, C.-M. Iron(III)-salan complexes catalysed highly enantioselective fluorination and hydroxylation of β-keto esters and *N*-Boc oxindoles. *Chem. Commun.* **50**, 7870–7873 (2014).
7. Allais, C., Nuhant, P. & Roush, W. R. (Diisopinocampheyl)borane-mediated reductive Aldol reactions of acrylate esters: enantioselective synthesis of *anti*-aldols. *Org. Lett.* **15**, 3922–3925 (2013).
8. Holan, M., Pohl, R., Císařová, I. & Jahn, U. Polyfunctional β-dicarbonyl compounds by Michael addition reactions of ester enolates to α-benzylidene and α-alkylidene-β-dicarbonyl compounds. *Eur. J. Org. Chem.* **18**, 3459–3475 (2012).
9. Taber, D. F., Sheth, R. B. & Joshi, P. V. Simple preparation of α-diazo esters. *J. Org. Chem.* **70**, 2851–2854 (2005).
10. Yamashita, M. *et al*. Practical and highly stereoselective synthesis of trisubstituted (*E*)-α, β-unsaturated esters. *Synthesis* **47**, 3392–3402 (2015).
11. Kukula, O., Matoušek, V., Mallat, T & Baiker, A. Structural effects in the Pd-induced enantioselective deprotection–decarboxylation of β-ketoesters. *Tetrahedron: Asymmetry* **18**, 2859–2868 (2007).
12. Hirai, Y., Aida, T. & Inoue, S. Artificial photosynthesis of β-ketocarboxylic acids from carbon dioxide and ketones via enolate complexes of aluminum porphyrin. *J. Am. Chem. Soc.* **111**, 3062–3063 (1989).
13. Kurouchi, H., Sugimoto, H., Otani, Y. & Ohwada, T. Cyclization of arylacetoacetates to

indene and dihydronaphthalene derivatives in strong acids. evidence for involvement of further protonation of O,O-diprotonated β-ketoester, leading to enhancement of cyclization. *J. Am. Chem. Soc.* **132**, 807–815 (2010).

14. Zhang, Y., Shibatomi, K. & Yamamoto H. Lewis acid-mediated selective chlorinations of silyl enolate. *J. Am. Chem. Soc.* **126**, 15038–15039 (2004).
15. Bélanger, É., Houzé, C., Guimond, N., Cantin, K. & Paquin, J.-F. Unexpected effect of the fluorine atom on the optimal ligand-to-palladium ratio in the enantioselective Pd-catalyzed allylation reaction of fluorinated enol carbonates. *Chem. Commun.* 3251–3253 (2008).
16. Aitken, K. M. & Aitken, R. A. Rearrangement during halogenation of 2-hydroxy-1,2-diphenylpropan-1-one (α-methylbenzoin). *Tetrahedron* **64**, 5217–5220 (2008).
17. Lauriers, A. J.-D. & Legault, C. Y. Iodine(III)-mediated oxidative hydrolysis of haloalkenes: access to α-halo ketones by a release-and-catch mechanism. *Org. Lett.* **18**, 108–111 (2016).
18. Liu, J., Li, W., Wang, C., Li, Y. & Li, Z. Selective 1,2-dihalogenation and oxy-1,1-dihalogenation of alkynes by *N*-halosuccinimides. *Tetrahedron Lett.* **52**, 4320–4323 (2011).
19. Reddy, M. S. N., Reddy, B. K., Reddy, C. K., Kumar, M. K., Rajan, S. T. & Reddy, M. S. An efficient industrial process for the preparation of fluvastatin sodium. *Orient. J. Chem.* **24**, 167–174 (2008).
20. Ahlsten, N., Gómez, A. B. & Martín-Matute, B. Iridium-catalyzed 1,3-hydrogen shift/chlorination of allylic alcohols. *Angew. Chem. Int. Ed.* **52**, 6273–6276 (2013).
21. Jing, Y., Daniliuc, C. G. & Studer, A. Direct conversion of alcohols to α-chloro aldehydes and α-chloro ketones. *Org. Lett.* **16**, 4932–4935 (2014).
22. Claraz, A., Landelle, G., Oudeyer, S. & Levacher, V. Asymmetric organocatalytic protonation of silyl enolates catalyzed by simple and original betaines derived from cinchona alkaloids. *Eur. J. Org. Chem.* **34**, 7693–7696 (2013).
23. Vita, M. V. & Waser, J. Azidation of β-keto esters and silyl enol ethers with a benziodoxole reagent. *Org. Lett.* **15**, 3246–3249 (2013).
24. Denmark, S. E., Rossi, S., Webstar, M. P. & Wang, H. Catalytic, enantioselective sulfenylation of ketone derived enoxysilanes. *J. Am. Chem. Soc.* **136**, 13016–13028 (2014).
25. Klahn, P., Erhardt, H., Kotthaus, A. & Kirsch, S. F. The synthesis of α-azidoesters and geminal triazides. *Angew. Chem. Int. Ed.* **53**, 7913–7917 (2014).
26. Steves, J. E. & Stahl, S. S. Copper(I)/ABNO-catalyzed aerobic alcohol oxidation: alleviating steric and electronic constraints of Cu/TEMPO catalyst systems. *J. Am. Chem.*

Soc. **135**, 15742–15745 (2013).
